# A chromosome-level genome assembly of *Platycladus orientalis* and comparative genomics reveal pivotal roles of transposable elements in gene duplication and pseudogenization across gymnosperm giga-genomes

**DOI:** 10.1016/j.xplc.2026.101814

**Published:** 2026-03-10

**Authors:** Yu-Tao Bao, Ren-Gang Zhang, Hui Liu, Zhi-Chao Li, Si-Qian Jiao, Kai-Hua Jia, Shan-Shan Zhou, Shuai Nie, Xue-Mei Yan, Tian-Le Shi, Xue-Chan Tian, Shi-Wei Zhao, Lei Kong, Zhao-Yang Chen, Hai-Yao Ma, Xiao-Lei Yang, Charles Chen, Yousry Aly El-Kassaby, Ilga Porth, Xiao-Ru Wang, Jian-Feng Mao, Wei Zhao

**Affiliations:** 1 State Key Laboratory of Tree Genetics and Breeding, National Engineering Research Center of Tree Breeding and Ecological Restoration, Beijing Advanced Innovation Center for Tree Breeding by Molecular Design, National Engineering Laboratory for Tree Breeding, Key Laboratory of Genetics and Breeding in Forest Trees and Ornamental Plants, Ministry of Education, College of Biological Sciences and Technology, Beijing Forestry University, Beijing 100083, China; 2 Yunnan Key Laboratory for Integrative Conservation of Plant Species with Extremely Small Populations/State Key Laboratory of Plant Diversity and Specialty Crops, Kunming Institute of Botany, Chinese Academy of Sciences, Kunming 650201, China; 3 University of the Chinese Academy of Sciences, Beijing 100049, China; 4 Department of Ecology and Environmental Science, Umeå Plant Science Centre, Umeå University, Umeå 90187, Sweden; 5 Pingdingshan University, Henan Province Key Laboratory of Germplasm Innovation and Utilization of Eco-economic Woody Plant, Pingdingshan 467000, China; 6 Institute of Crop Germplasm Resources, Shandong Academy of Agricultural Sciences, Ji’nan 250100, China; 7 Rice Research Institute, Guangdong Academy of Agricultural Sciences & Key Laboratory of Genetics and Breeding of High-Quality Rice in Southern China (Co-construction by Ministry and Province), Ministry of Agriculture and Rural Affairs & Guangdong Key Laboratory of New Technology in Rice Breeding, Guangzhou 510640, China; 8 State Key Laboratory of Vegetable Biobreeding, Institute of Vegetables and Flowers, Chinese Academy of Agricultural Sciences, Beijing 100081, China; 9 Key Laboratory of Biology and Genetic Improvement of Horticultural Crops (North China), Institute of Forestry and Pomology, Beijing Academy of Agriculture and Forestry Sciences, Beijing 100093, China; 10 School of Life Sciences and Medicine, Shandong University of Technology, Zibo, Shandong 255000, China; 11 National Tree Breeding Station for *Platycladus orientalis* in Jiaxian, Forest Farm of Jiaxian County, Jiaxian 467100, China; 12 Department of Biochemistry and Molecular Biology, Oklahoma State University, Stillwater, OK 74078, USA; 13 Department of Forest and Conservation Sciences, Faculty of Forestry, University of British Columbia, Vancouver, BC V6T 1Z4, Canada; 14 Département des Sciences du Bois et de la Forêt, Faculté de Foresterie, de Géographie et Géomatique, Université Laval, Québec, QC G1V 0A6, Canada; 15 Umeå Plant Science Centre, Department of Plant Physiology, Umeå University, 90187 Umeå, Sweden; 16 Laboratory of Tropical Forest Ecology, Xishuangbanna Tropical Botanical Garden, Chinese Academy of Sciences, Menglun, Yunnan, 666303, China

**Keywords:** transposable elements, gymnosperms, genome expansion, gene duplication, pseudogenization, gene fragment capture

## Abstract

Gymnosperms, particularly conifers, exhibit a high abundance of transposable elements (TEs) in their giga-scale genomes. TEs interact both antagonistically and cooperatively with the host genome, promoting structural and genetic innovations across evolutionary lineages. However, how TEs shape the coding space of gymnosperm genomes remains a key unresolved question. Here, we present a high-quality genome assembly for the keystone conifer *Platycladus orientalis*, with a contig N50 of 57.54 Mb—the highest continuity reported to date—to investigate the role of TEs. Comparative genomics confirms the absence of recent whole-genome duplication and the presence of genome expansion in gymnosperms, revealing complex interactions among recurrent TE proliferation, low DNA removal rates, and DNA methylation-mediated silencing. Computational evidence indicates that TE-mediated gene duplication and pseudogenization provide a genetic basis for adaptive evolution and functional innovation, significantly shaping gene family dynamics and the emergence of species-specific genes. Additionally, TEs capture and duplicate an average of ∼400,000 coding gene fragments per gymnosperm genome, facilitating exon shuffling and triggering epigenetic conflicts between source genes and captured exon fragments. Genes from which fragments are captured (donor genes) show significantly higher levels of exon methylation than genes not captured by TEs (free genes), whereas syntenic donor genes exhibit lower levels of silencing responses than non-syntenic donor genes. This study provides valuable genomic resources and offers insights into the evolutionary patterns and principles underlying the large genome size and complexity of gymnosperms.

## Teaser

This study presents a chromosome-level genome assembly for the keystone conifer *Platycladus orientalis,* representing the highest level of continuity achieved in a gymnosperm genome assembly to date. Extensive comparative genomic analyses reveal that transposable elements (TEs) act as key drivers of giga-genome evolution in gymnosperms by mediating widespread gene duplication, pseudogenization, and coding fragment capture. These findings uncover the intricate evolutionary and epigenetic interplay between TEs and host genomes and provide important insights into the genetic basis of adaptive innovation in gymnosperms.

## Introduction

Transposable elements (TEs) are DNA sequences capable of moving, replicating, and integrating into new positions within the host genome, and they represent widespread components of plant genomes (Tenaillon et al., 2010). TEs are also important drivers of genome evolution and variation, as they can induce mutations, alter gene expression, and reorganize genome structures (Galindo-González et al., 2017; Almojil et al., 2021). Systematic identification and quantification of TE-mediated gene duplication, pseudogenization, and capture–duplication events are therefore essential for understanding the mechanistic roles of TEs in shaping genomic complexity.

One prominent consequence of TE amplification, particularly of long terminal repeat retrotransposons (LTR–RTs), is the expansion of genome size (Beric et al., 2021). TEs often proliferate at rates that exceed their removal, leading to their continuous accumulation and contributing to genome expansion, as observed in gymnosperms (Wan et al., 2022). Moreover, TE activity can result in insertions within genes (Wang et al., 2023; Tian et al., 2024), in gene-proximal regions (Butelli et al., 2012; Xu et al., 2023), or at distant genomic regions (Zhang et al., 2021, 2022b). Such insertions can generate gene mutations and phenotypic variation through various mechanisms, such as alternative splicing (Xia et al., 2024), premature translation termination (Tian et al., 2024), reconfiguration of regulatory region functions (Zhang et al., 2021), and alterations in regional methylation levels (Hollister et al., 2011). Additionally, TEs are now recognized as evolutionary forces shaping genome structure by contributing to centromere formation and intron structure (Liu et al., 2021c; Wan et al., 2022), mediating chromosomal rearrangements (Lisch, 2013), facilitating coding gene duplication or pseudogenization (Wang et al., 2022; Nie et al., 2023; Jang et al., 2024), and promoting exon shuffling through the capture, mobilization, and duplication of gene fragments (Jiang et al., 2004; Muyle et al., 2021; Ma et al., 2023). These effects point to TEs as major contributors to shaping the space for coding genes.

As one of the significant plant lineages on Earth, extant gymnosperms comprise approximately 1100 species, representing four major lineages: conifers, cycads, Ginkgo, and gnetophytes (Christenhusz et al., 2011). Conifers are classified into two clades, conifer I and conifer II, which are widely distributed across diverse habitats in both the Northern and Southern Hemispheres, occupying 39% of the global forest area and exhibiting significant ecological and economic value (Wang and Ran, 2014; Ohri, 2021). The Cupressaceae (conifer II) represents the largest group among conifers. *Platycladus orientalis*, a representative Cupressaceae species, is characterized by its broad distribution range, drought resistance, adaptability, and ability to thrive under nutrient-poor conditions, making it an important afforestation species in the rocky mountain regions of northern China (Jia et al., 2020).

Gymnosperm genomes are large (ranging from 4 to 37 Gb) and feature a high proportion of repetitive sequences, predominantly TEs, which account for up to 75% of the genome on average (Ahuja and Neale, 2005). This greatly complicates the elucidation of their genomic architecture and poses significant challenges for generating high-quality genome assemblies (Wan et al., 2022). To date, only about 30 gymnosperm genomes have been released, which remains insufficient for a comprehensive understanding of the genomic characteristics of gymnosperms. Compared with angiosperms, gymnosperms generally exhibit larger nuclear volumes, higher TE content, larger gene sizes, and expanded gene families resulting from gene duplication (Ahuja and Neale, 2005; Ohri, 2021). In addition, gymnosperms have not undergone recent whole-genome duplication (WGD) events (Liu et al., 2021b, 2022; Wan et al., 2021; Niu et al., 2022), which have played crucial roles in large-scale gene duplication and adaptive evolution in angiosperms (Van de Peer et al., 2017). These observations suggest that gymnosperms have followed an evolutionary trajectory distinct from that of angiosperms (De La Torre et al., 2020), with unique drivers shaping their large and complex genomes. However, the evolutionary basis underlying their broad distribution remains to be elucidated.

The question of whether and how transposons drive the evolution of complex genomes in gymnosperms remains unresolved. In this study, we generated a chromosome-scale reference genome assembly for *P*. *orientalis*, which exhibits the highest continuity among currently available gymnosperm genome assemblies. As the sole species of *Platycladus* (Cupressaceae), this assembly provides a valuable resource for investigating conifer genome evolution. Furthermore, we utilized a standardized approach to conduct a comparative analysis of TEs across 12 gymnosperms and other representative vascular plants, focusing on TE composition, insertion patterns, and evolutionary dynamics, as well as their potential contributions to gene duplication, gene pseudogenization, and gene fragment capture–duplication. Our study characterizes the intricate interplay between transposons and protein-coding gene space in gymnosperms, highlighting the critical roles of TEs in shaping genome evolution and functional diversity in gymnosperms.

## Results

### Genome assembly and annotation

**Figure 1:**
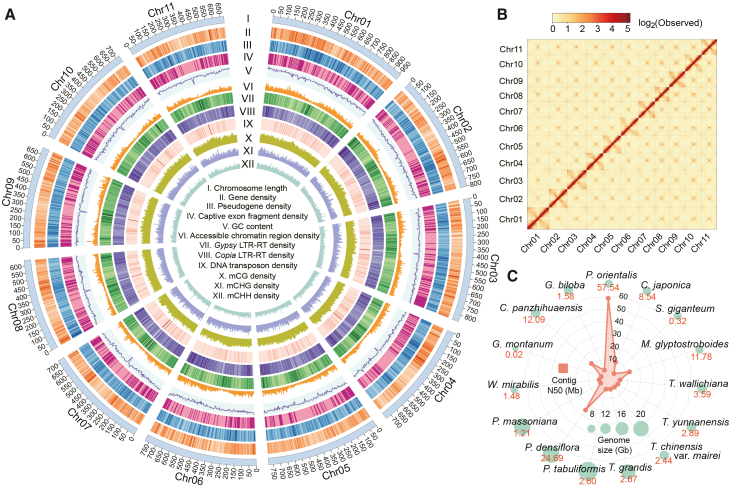
High-quality genome assembly of *P*. *orientalis*. **(A)** Genome landscapes shown using 5 Mb windows across the 11 assembled pseudochromosomes. **(B)** Genome-wide chromatin interactions based on Hi-C data. The heatmap represents contact matrices generated by aligning Hi-C data to the chromosome-scale assembly of the *P*. *orientalis* genome. Color intensity corresponds to the log_2_(observed) value, representing the base-2 logarithmic transformation of the observed contact frequency. **(C)** Comparison of contig N50 sizes among finalized assemblies of published chromosome-level gymnosperm genomes.

We assembled a chromosome-level genome of *P*. *orientalis* using *de novo* sequencing (Supplemental Note 1). Approximately 100× coverage of Oxford Nanopore Technologies (ONT) reads, ∼30× coverage of PacBio single-molecule real-time (SMRT) reads, ∼57× coverage of PCR-free Illumina paired-end reads, and ∼110× coverage of high-throughput chromosome conformation capture (Hi-C) sequencing data were generated (Supplemental Table 1). After contig assembly, comparison, correction, polishing, and scaffolding (Supplemental Figure 1), 99.71% (8.37 Gb) of the assembled sequences were anchored to 11 chromosomes, consistent with the chromosome number of the haploid genome (Figures 1A and 1B). The final assembled genome size was 8.39 Gb (Supplemental Table 2), which closely matches the estimated genome size of 8.22–8.47 Gb obtained with the 17-mer counting (Supplemental Figure 2). The assembly achieved a contig N50 of 57.54 Mb (Supplemental Table 3), representing the highest contiguity among currently available gymnosperm genome assemblies (Figure 1C). Multiple assessments confirmed the high completeness and accuracy of the reference assembly (Supplemental Note 2). For instance, the Compleasm score (a faster and more accurate reimplementation of BUSCO) (Huang and Li, 2023) reached 92.0% (Supplemental Figure 3; Supplemental Table 4), and Merqury evaluation (Rhie et al., 2020) yielded a quality value (QV) of 38.19, corresponding to single-base accuracy exceeding 99.9% (Supplemental Figure 4A). Both ONT and Illumina reads demonstrated high mapping rates of 99.54% and 99.63%, respectively, with genome coverage of at least 20-fold for 99.73% and 96.44% of the assembly (Supplemental Table 5). Additionally, strong collinearity was observed between the assembled chromosomes and the previously established genetic map (Jin et al., 2019) (Supplemental Figure 4B).

A total of 52 897 protein-coding genes were annotated using *de novo* prediction, protein homology searches, and transcriptome-based methods (Supplemental Note 3 and Supplemental Table 6). The complete BUSCO and OMArk recovery scores reached 90.90% and 93.64%, respectively, demonstrating superior performance compared with most published gymnosperm genomes (Supplemental Figure 3 and Supplemental Tables 7 and 9). In addition, 547 rRNAs, 3367 tRNAs, and 684 other non-coding RNAs were predicted (Supplemental Table 10). Using stringent quality control in the PseudoPipe pipeline (Zhang et al., 2006), 395,147 pseudogenes were annotated, including 21 250 duplicated pseudogenes (DUPs), 224,957 processed pseudogenes (PSSDs), and 148,940 pseudogenic fragments (Figure 1A and Supplemental Table 12). Through an integrated computational pipeline for whole-genome transposon annotation (Supplemental Figure 5), 5.81 Gb of TE sequences (69.28% of the genome; 13 485 169 elements) were identified in *P*. *orientalis* (Figure 1A and Supplemental Table 14). Among these, LTR–RTs were the most abundant, accounting for 49.23% of the genome, with *Ty3/Gypsy* elements (30.50%) exceeding *Ty1/Copia* elements (17.81%). Additionally, DNA transposons accounted for 24.42%, whereas uncharacterized TEs comprised only 0.34% of the genome.

### Gene family dynamics, synteny, and WGD

**Figure 2:**
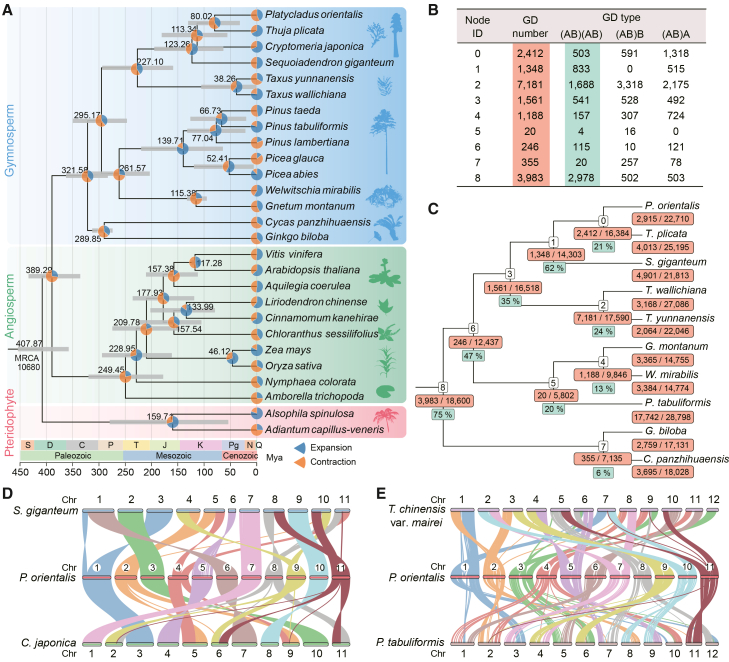
Gene families and gene synteny among representative vascular plants. **(A)** Phylogenetic tree and inference of gene family expansion and contraction across 27 representative plant genomes. The maximum-likelihood phylogeny was inferred from the alignment of concatenated nucleotide sequences of 692 single-copy orthologs. Numbers and gray bars alongside nodes represent estimated divergence times and associated 95% confidence intervals based on 4d sites. Gene family analysis began with 10 680 orthologous groups conserved in the most recent common ancestor (MRCA) of the studied vascular plants. Pie charts show the proportions of gene families that underwent expansion or contraction. S, Silurian; D, Devonian; C, Carboniferous; P, Permian; T, Triassic; J, Jurassic; K, Cretaceous; Pg, Palaeogene; N, Neogene; Q, Quaternary; mya, million years ago. Plant silhouettes were obtained from PhyloPic (http://phylopic.org). Whole-genome duplication events were detected at focal nodes using the phylogenomic Tree2GD approach. **(B)** Summary of duplication types showing the numbers of orthologous groups (OGs) at the corresponding nodes. **(C–E) (C)** Orange bars indicate the number of duplicated gene families relative to the total number of gene families. Green bars indicate the percentage of (AB) (AB) types among duplication nodes. Genome collinearity between the chromosomes of *P*. *orientalis* and those of **(D)***C*. *japonica* and *S*. *giganteum*, and **(E)***T*. *chinensis* var. *mairei* and *P*. *tabuliformis*. Lines represent homologous genomic blocks.

We identified 54 368 orthogroups (gene families) across 27 representative vascular plant species (Figure 2A and Supplemental Tables 15 and 16). Phylogenetic reconstruction and molecular dating based on 692 single-copy orthologs indicated that *P*. *orientalis* is most closely related to *Thuja plicata*, which belongs to the conifer II clade. The split between conifer I and conifer II was estimated to have occurred approximately 295.17 million years ago (mya). Significantly expanded gene families and species-specific genes in *P*. *orientalis* were strongly enriched (adjusted *p* < 0.05) in key pathways associated with plant growth and development, biosynthesis and metabolism, immunity, and defense (Supplemental Note 4 and Supplemental Tables 18 and 20).

Several WGD events have been identified in gymnosperms, although some remain controversial (Qiao et al., 2022; Wan et al., 2022). We sought to detect evidence of WGD events in *P*. *orientalis* by identifying paralogous syntenic gene blocks. However, only 13 syntenic blocks and 157 syntenic genes were detected based on BLASTP alignments using MCScanX (Wang et al., 2012b), with consistent results obtained from Whole-Genome Duplication Identification analysis (WGDI) (Sun et al., 2022) (Supplemental Table 21). The distribution of synonymous nucleotide substitution (*K*s) values for syntenic gene pairs within *P*. *orientalis* and between *P*. *orientalis* and 11 other gymnosperm genomes revealed two *K*s peaks in *P*. *orientalis* (Supplemental Figure 8), corresponding approximately to 1.5–2.0 and <1.0, respectively. We further validated these ancient signals using the Tree2GD phylogenomic method (Figures 2B and 2C). The ancestor of gymnosperms (node 8) contained 21.41% (3983) duplicated genes, with 75% classified as (AB)(AB)-type duplicates. This finding suggests a common WGD event in gymnosperms, corresponding to the *K*s peak at 1.5–2.0 (Liu et al., 2022). In general, more recent WGD events tend to leave more extensive traces in the genome. Nevertheless, the Cupressaceae ancestor (node 1) retained a lower proportion of duplicated genes (9.42%) and a smaller fraction of (AB)(AB)-type duplicates (62%) compared with node 8. More importantly, the syntenic depth between *P*. *orientalis* and newly assembled gymnosperm genomes was consistently 1:1, aligning with findings reported in a recent study (Zhang et al., 2024). Only 2%, 5%, and 7% of orthologous genes in *Taxus chinensis* var. *mairei*, *Pinus tabuliformis*, and *Cycas panzhihuaensis*, respectively, exhibited one-to-more (≥2) syntenic blocks relative to the *P*. *orientalis* genome (Supplemental Figures 9 and 10 and Supplemental Table 22). Thus, we hypothesize that the *K*s peak below 1.0 in *P*. *orientalis* does not represent an ancient WGD but more likely reflects segmental duplications (SDs). In summary, *P*. *orientalis* did not undergo recent WGDs but likely experienced the ancient WGD event shared by all gymnosperms.

Synteny analysis between *P*. *orientalis* and 10 recently assembled chromosome-level gymnosperm genomes revealed varying degrees of genome reorganization through chromosomal exchanges during gymnosperm evolution (Supplemental Figure 10). Chromosomes showed rearrangements, inversions, expansions, and contractions (Figures 2D and 2E and Supplemental Figure 11). Overall, inter-family chromosomal rearrangements occurred more frequently than intra-family rearrangements. The evolution of conifers with 11 and 12 chromosomes involved complex and diverse chromosomal rearrangements, as detailed in Supplemental Note 4.

### TE accumulation landscapes among gymnosperms

**Figure 3:**
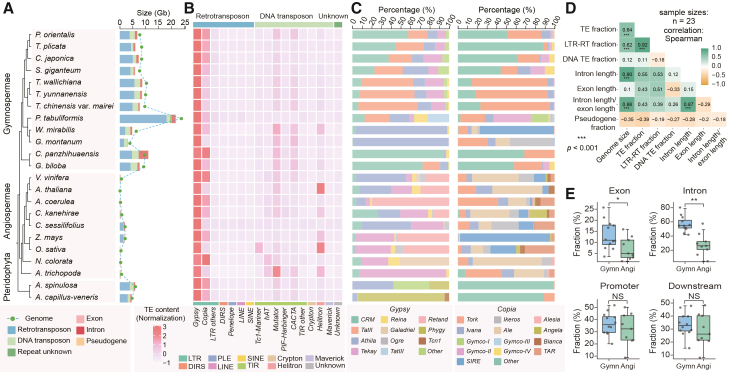
TE landscapes across 23 vascular plants. **(A)** Phylogenetic relationships among the 23 species and the composition of various genomic features. **(B)** Proportion of each TE superfamily within the genomes. **(C)** Proportional contribution (%) of each intact *Ty3/Gypsy* and *Ty1/Copia* family to the cumulative length of intact LTR–RTs in each gymnosperm species. **(D)** Correlations among genome size, TE fraction, LTR–RT fraction, DNA TE fraction, intron length, exon length, intron length/exon length ratio, and pseudogene fraction across the 23 species, without accounting for phylogenetic non-independence. **(E)** Comparison of the genomic fractions of TEs located in exon, intron, promoter (2 kb upstream of genes), and downstream (2 kb) regions between representative gymnosperms and angiosperms. Mann–Whitney U test: ^∗^*p* < 0.05, ^∗∗^*p* < 0.01, and NS, no significant difference. Gymn, Gymnospermae; Angi, Angiospermae.

Employing a common computational pipeline for genome-wide TE annotation across all studied species (Supplemental Figure 5), the TE content across 21 seed plants was determined to range from 16.23% (*Arabidopsis thaliana*) to 82.38% (*Pinus tabuliformis*) (Supplemental Table 14), with gymnosperms displaying substantially higher TE content on average (70.32%) than angiosperms (38.41%). The TE superfamily composition and proliferation patterns were relatively similar across species, with LTR–RTs predominating (Supplemental Tables 14 and 23). Certain DNA transposon superfamilies, such as *Mutator*, *CACTA*, and *Helitron*, also contributed significantly to the genomes of gymnosperms (e.g., conifers and Gnetales) and angiosperms (e.g., *A*. *thaliana* and *Oryza sativa*) (Figures 3A and 3B). Phylogenetic analysis of the superfamilies revealed clade-specific expansions of RTs in *P*. *orientalis* (Cupressaceae) and *P*. *tabuliformis* (Pinaceae) (Supplemental Figure 12 and Supplemental Table 24). Further comparisons across vascular plants suggested large differences in LTR–RT composition. Intragenus comparisons within Taxus revealed similar family proportions of *Gypsy* and *Copia*, whereas interfamilial or interordinal disparities were pronounced (Figure 3C and Supplemental Tables 25 and 26). Several lineage-specific LTR–RT families were identified. For example, the *Gypsy* families *TatII* and *TatIII* and the *Copia* families *Gymco-I* to *Gymco-IV* are unique to gymnosperms, with *Gymco-I* and *Gymco-III* restricted to conifers. In contrast, angiosperms harbor unique *Gypsy/Retand* and *Copia* families, including *Angela*, *Bianca*, and *TAR*, whereas ferns contain the *Gypsy* families *Phygy* and *Tcn1*.

In gymnosperms, TEs experienced continuous expansion and removal over the past 200 million years (Supplemental Figure 13A), in contrast to other vascular plants (Supplemental Figure 13C). Partial deletions (generating truncated LTR–RTs, *T*) and unequal or illegitimate homologous recombination events (resulting in solo-LTRs, *S)* represent the predominant mechanisms for the reduction of intact LTR–RT (*I*) copies (Hawkins et al., 2009). Comparative analyses across major gymnosperm lineages revealed higher LTR–RT generation rates (*I* + *S + T*) and lower removal rates (*S/I*, *T/I*, and [*S + T*]*/I*) in conifers, Ginkgo, and cycads, facilitating TE maintenance (Supplemental Figures 14A and 14B). *P*. *tabuliformis* exhibited particularly pronounced accumulation and lower removal rates (Supplemental Table 27), consistent with its recent TE expansion (0–10 mya; Supplemental Figure 13A). Conversely, Gnetales showed the lowest proliferation and the highest removal rates, resulting in the smallest genomes. Most studied gymnosperms experienced two LTR–RT amplification periods (∼180–200 and ∼60–80 mya) and one DNA transposon amplification period (∼120–160 mya). These periods correspond to the warm, humid Jurassic, when gymnosperms dominated terrestrial ecosystems (Vermeij, 2008), and to the environmentally turbulent Cretaceous–Paleogene (K–Pg) boundary associated with mass extinction events. Extant genomes indicate intact LTR–RT transposition events occurring 10–30 mya (Supplemental Figures 13B, 13D, and 15), coinciding with the Miocene epoch (5.33–23.03 mya), which was characterized by a global cooling trend toward ice-age conditions (Steinthorsdottir et al., 2021). Overall, these findings suggest a potential environmental influence on gymnosperm TE dynamics and genome evolution, consistent with previous reports (Lou et al., 2023).

With or without accounting for phylogenetic non-independence, TE content, LTR–RT content, intron length, and the intron length/exon length ratio were all significantly positively correlated with genome size (Figure 3D and Supplemental Figure 14C). Upon further examination of TE insertions within genic and flanking regions, we found no significant differences in TE fractions within promoter and downstream regions between representative gymnosperms and angiosperms (Figure 3E and Supplemental Table 28). Nevertheless, gymnosperms exhibited a markedly higher abundance of TEs in exonic and intronic regions compared with angiosperms. Specifically, TEs accounted for more than 40%, and occasionally up to 80%, of intronic sequences in gymnosperms. The median intron length in these plants was 6.13 kb, which was significantly larger than the 0.74 kb observed in representative angiosperms. In summary, genome expansion has occurred both in intergenic regions and within gene regions, and both patterns are closely associated with TE activity.

### Prevalence of TEs in *P*. *orientalis*

**Figure 4:**
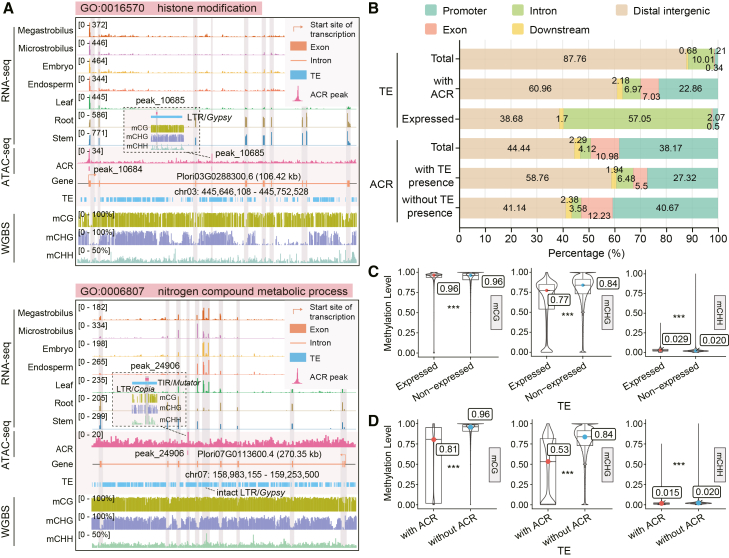
TE characterization in *P*. *orientalis*. **(A)** IGV visualization of expression, chromatin accessibility, and methylation levels in two ultra-long coding genes (>100 kb) containing TE insertions. **(B)** Subgenomic distributions of total TEs, TEs with ACRs, expressed TEs, total ACRs, ACRs containing TEs, and ACRs without TE presence. Elements are preferentially distributed in the following order: promoter (2 kb upstream) > exon > intron > downstream > distal intergenic regions. **(C)** Patterns of CG, CHG, and CHH DNA methylation in expressed and non-expressed TEs. **(D)** Patterns of CG, CHG, and CHH DNA methylation in TEs with and without ACRs. Mann–Whitney–Wilcoxon test: ^∗∗∗^*p* < 0.001.

A total of 1 643 119 TE copies (615.83 Mb), representing 12.18% of all TE copies and 10.59% of the total TE length, were identified in the genic and flanking regions of *P*. *orientalis*, with the majority (84.19% of copies) located within introns (Supplemental Figure 16A). Additionally, TE insertions were detected in 44 924 (84.93%) and 44 060 (83.29%) coding genes within the promoter and downstream regions, respectively (Supplemental Figure 16B). Strikingly, among the 51 839 coding genes with TE insertions in either genic or flanking regions, up to 69.40% showed detectable expression (transcripts per million [TPM] > 0) in at least one of the seven tissues examined (megastrobilus, microstrobilus, embryo, megagametophyte, leaf, root, and stem). This phenomenon is likely attributable to the stringent epigenetic regulation of these TEs. Whole-genome bisulfite sequencing (WGBS) revealed average methylation levels of 88.78%, 74.57%, and 2.21% in the CG (cytosine-guanine), CHG (cytosine-H-guanine), and CHH (cytosine-H-H) contexts (where H represents any nucleotide except guanine), respectively, across the *P*. *orientalis* genome (Figure 1A, Supplemental Figure 17, and Supplemental Table 30). The mCG and mCHG methylation levels were higher than those reported in most plant studies and comparable to those observed in other large (>10 Gb) and TE-rich (>60%) genomes (Ausin et al., 2016; Niederhuth et al., 2016; Xiao et al., 2023). The mean methylation levels of TEs in the CG, CHG, and CHH contexts were 92.95%, 81.14%, and 2.32%, respectively. Among the 3489 ultra-long coding genes (> 100 kb) containing TE insertions, 88.02% showed detectable expression signals. In addition, certain TEs were found to be expressed or to generate accessible chromatin regions (ACRs) enriched for *cis*-regulatory elements (Figure 4A and Supplemental Figure 18).

Further analysis revealed that, of the 1 350 627 (10.02%) TEs with transcriptional evidence, 52 269 (0.39%) showed counts per million (CPM) > 1, predominantly located in intronic (57.05%) and distal intergenic regions (38.68%) (Figure 4B). Among these, 48.92% showed high tissue-preferential expression (tau > 0.9), indicating greater tissue specificity than that observed for coding genes (Supplemental Figure 19A). Reproductive organs were the primary tissues exhibiting TE tissue-specific expression (tau = 1), with 30.99% of the tissue-specific TE expression detected in the megastrobilus and 26.39% in the microstrobilus (Supplemental Figure 19B). The methylation levels within the bodies of expressed TEs were significantly reduced in the mCHG context (Figure 4C). Across the four tissues examined—megastrobilus, microstrobilus, embryo, and leaf—approximately 17.37% (16.38%–19.48%) of the annotated ACRs overlapped with TEs, and about 8.92% (7.20%–11.37%) of ACR peaks overlapped with TEs (Supplemental Figure 20). TEs contributed to ACR formation in promoter regions (27.32%) and predominantly in distal intergenic regions (58.76%) (Figure 4B), consistent with findings in mammals and rice, where TE-derived ACRs function as potential enhancers (Ye et al., 2020; Zhang and Zhang, 2022). Moreover, numerous potential transcription factor binding sites (e.g., Dof, ERF, and MIKC_MADS) were identified within TE-associated ACRs (Supplemental Table 31). The methylation levels of ACR-associated TEs were markedly lower than those of non-ACR TEs (Figure 4D).

### TE-mediated gene duplication

Most genes across the 12 gymnosperms (69.30% in *Ginkgo biloba* to 85.15% in *Taxus wallichiana*) occurred as duplicates within gene families (Figure 6A; Supplemental Table 32). The relative contributions of five duplication modes (Supplemental Figure 21)—WGD or SD, tandem duplication (TD), proximal duplication (PD), transposed duplication (TRD), and dispersed duplication (DSD)—varied across genomes. Overall, however, DSDs and TRDs contributed substantially to gene duplication in these gymnosperms (Figure 6A and Supplemental Figure 22). Interestingly, TDs contributed profoundly in *Cryptomeria japonica and Sequoiadendron giganteum*, generating the largest numbers of duplicate genes in these genomes. *K*s analysis revealed peak values around 0.1–0.2 (Figure 6B and Supplemental Figure 23), indicating the prevalence of recent small-scale duplications during gymnosperm evolution.

**Figure 5:**
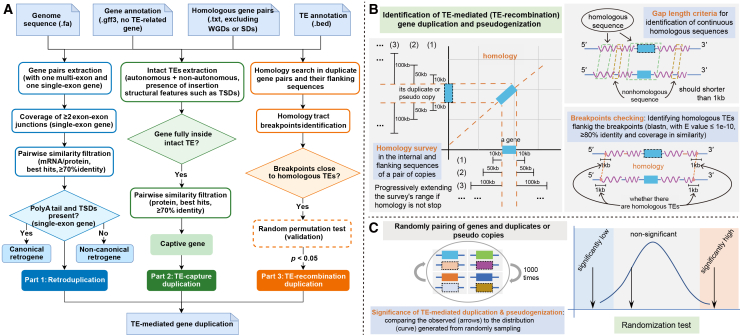
Identification pipeline and validation of TE-mediated gene duplication and pseudogenization. **(A)** Schematic overview of the pipeline used to detect and classify TE-mediated gene duplications into three mechanisms: retroduplication, TE-capture duplication, and TE-recombination duplication. **(B)** Schematic diagram illustrating the genomic features and identification process of TE-mediated (TE-recombination) gene duplication and pseudogenization. **(C)** Statistical validation of TE-recombination-mediated duplications using a randomization test.

Evidence of TE-mediated gene duplication (Figure 5A, see materials and methods) was observed across the four small-scale duplication modes (TD, PD, TRD, and DSD) in the 12 investigated gymnosperms. Approximately 19.59% (range: 7.48%–42.44%) of small-scale duplicated genes were derived from TE-mediated duplication events (Supplemental Table 32). Substantial heterogeneity was detected in the generation of retrogenes and captive genes through retroduplication and TE-capture duplication, respectively (Supplemental Table 33). A significant number of retrogenes were found to span at least two exon–exon junctions of their parental loci. These cases were predominantly classified as non-canonical forms, characterized by the absence of poly(A) tails and/or target site duplications (TSDs). Notably, TRDs and DSDs were consistently identified as the predominant categories in both mechanisms; conversely, minor contributions were made to TDs and PDs, highlighting the dispersive nature of TE-mediated expansion.

**Figure 6:**
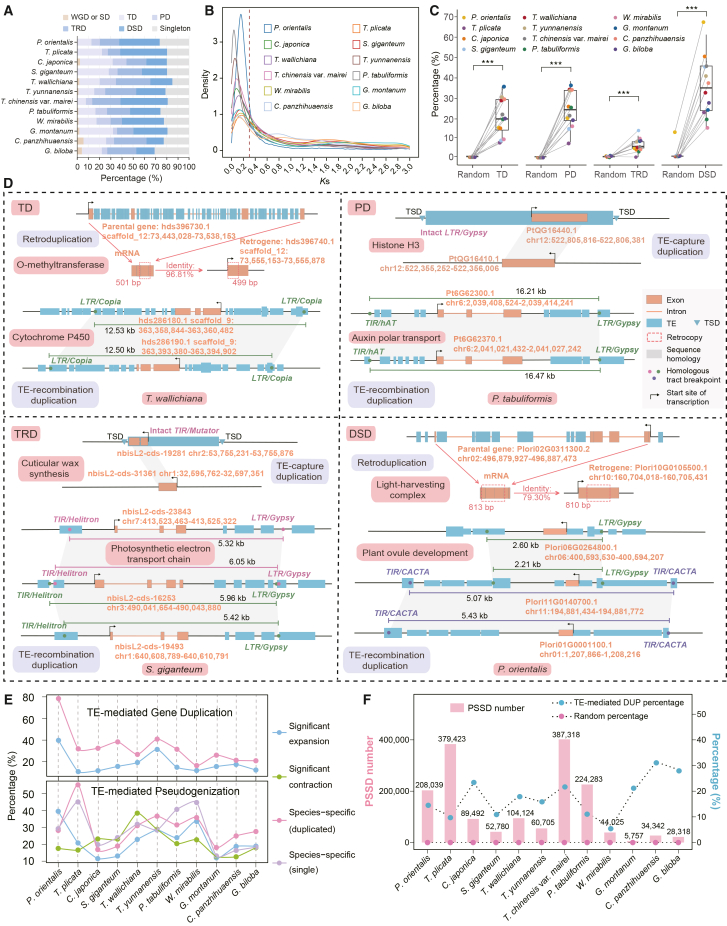
TE-mediated gene duplication and pseudogenization. **(A)** Relative abundance of six types of genes across 12 gymnosperms. Each gene duplication event was assigned to only one category to avoid redundancy, following the hierarchy: whole-genome duplication (WGD) or segmental duplication (SD) > tandem duplication (TD) > proximal duplication (PD) > transposed duplication (TRD) > dispersed duplication (DSD). **(B)***K*s distribution of duplicated gene pairs from 12 gymnosperms. **(C)** Mann–Whitney U test comparing the proportions of TE-recombination-mediated gene duplications across four duplication modes (TD, PD, TRD, and DSD) with those of randomly selected gene pairs. TE-recombination events were identified by strong TE homology at or immediately adjacent to the breakpoints of homologous tracts. ^∗∗∗^*p* < 0.001. **(D)** Examples of TE-mediated gene duplications from 4 conifer genomes. **(E)** Percentages of genes derived from TE-mediated gene duplication and pseudogenization events contributing to significantly expanded gene families, significantly contracted gene families, species-specific duplicated genes, and species-specific singleton genes. **(F)** TE-mediated pseudogenization: relative abundance of processed pseudogenes (PSSDs) and TE-mediated duplicated pseudogenes (DUPs) across different gymnosperm genomes.

Regarding homologous TE sequence-mediated recombination duplications (Figure 5B), a high proportion (68.56%, 55.82%, and 47.42%, respectively) of relatively recent DSD pairs (*K*s <0.3) in *P*. *orientalis*, *C*. *japonica*, and *T*. *chinensis* var. *mairei* contained homologous TE sequences at or immediately adjacent to the homologous tract breakpoints (Supplemental Table 34). Remarkable proportions of other TE-mediated duplication types were also detected, including TDs in *Taxus yunnanensis* (33.16%), PDs in *Cycas panzhihuaensis* (41.87%), and TRDs in *Welwitschia mirabilis* (14.49%). Considering the widespread presence of TEs across gymnosperms, a randomization test was performed 1000 times for each small-scale gene duplication mode in every species to validate these findings while accounting for the potential confounding effects of high genomic TE density (Figure 5C). The test was conducted under the explicitly defined null hypothesis (*H*_0_) that the proportion of gene pairs harboring homologous TEs at homologous recombination breakpoint regions would not differ significantly from that expected by chance (i.e., in randomly paired gene sets). However, the mean proportion of homologous TEs observed in randomly selected gene pairs was significantly lower than that observed in the actual gene duplicate pairs (*p* < 0.001) (Figure 6C and Supplemental Table 34). Consequently, the null hypothesis was rejected, confirming a non-random association driven by TE-mediated mechanisms and underscoring the pivotal role of TEs in gymnosperm gene duplication processes. Most TE families participated in gene duplication events (Supplemental Figure 24), although some families showed particularly strong associations with specific duplication types. For instance, *Mutator* DNA transposons were prominently involved in DSDs in *P*. *orientalis*, whereas *Copia* RTs and *Tc1-Mariner* DNA transposons contributed considerably to TRDs in *S*. *giganteum* and *W*. *mirabilis*, respectively (Supplemental Figure 25).

TE-mediated duplicate gene pairs exhibited higher *K*a/*K*s ratios, indicating either relaxed purifying selection or stronger positive selection (Supplemental Figure 26). Functional enrichment analysis revealed that these TE-mediated duplicated genes vary across species, involving diverse biological processes, including environmental stress responses (e.g., biotic and abiotic stimuli), secondary metabolite biosynthesis (e.g., terpenoids and flavonoids), and specific developmental regulation (e.g., auxin and reproduction). These findings highlight the pivotal role of TEs in driving adaptive evolution and functional innovation across gymnosperms (Supplemental Table 35). Representative examples of TE-mediated gene duplications are illustrated (Figure 6D and Supplemental Figure 27). For instance, TE-mediated recombination was found to facilitate the duplication of functional genes, including cytochrome P450 (TD) in *T*. *wallichiana*, auxin polar transport genes (PD) in *P*. *tabuliformis*, three copies of photosynthetic electron transport chain genes (TRDs) in *S*. *giganteum*, and three copies of ovule development genes (DSDs) in *P*. *orientalis*. Furthermore, alternative mechanisms were evident, including TE-capture-mediated duplication of histone H3 (PD) and cuticular wax synthesis genes (TRD), as well as retroduplication of O-methyltransferase (TD) and light-harvesting complex genes (DSD). Across gymnosperms, a considerable proportion of significantly expanded genes (18.26% on average) and species-specific duplicated genes (33.19% on average) were associated with TE-mediated duplication (Figure 6E and Supplemental Table 36). In *P*. *orientalis*, in particular, the contribution of TE-mediated gene duplication reached 39.78% and 78.41%, respectively (Supplemental Figure 28).

### TE-mediated pseudogenization

Pseudogenes were identified across 23 vascular plant species using a common procedure (see materials and methods), and abundant evidence of TE-mediated pseudogenization was uncovered. PSSDs, generated through RT-mediated reverse transcription of mRNA into cDNA, were abundant across the genomes (Figure 6F). Moreover, gymnosperms possessed a considerable fraction of repetitive genes (Figure 5B and Supplemental Figure 29), which underwent TE-mediated gene duplication followed by mutations in coding or regulatory regions, ultimately leading to functional impairment and the formation of 6.46%–32.62% DUPs (Supplemental Table 12). Randomization tests further revealed a significantly reduced occurrence of both homologous tracts and homologous TE sequences at breakpoints in randomly selected pseudo-source gene pairs across all species compared with that observed in true pseudo-source gene pairs (*p* < 0.001) (Figure 6F and Supplemental Table 12).

TE-mediated pseudogenization occurred in 12.58%–38.36% of genes from significantly contracted families across gymnosperms, curbing their gene family sizes (Figure 6E and Supplemental Table 36). Additionally, 11.51%–39.43% of significantly expanded genes and 16.88%–55.04% of species-specific duplicated genes may also have undergone TE-mediated pseudogenization. A range of 13.13%–44.97% of species-specific single-copy genes were found to harbor pseudogenes generated through TE-mediated mechanisms, indicating that TE activity may enable species-specific duplicated gene families to be reduced to single-copy genes. TE-mediated pseudogenization events significantly impacted various plant growth and developmental processes, including DNA replication, metabolic processes, ovule development, meiosis, hormone regulation, and post-transcriptional regulation (Supplemental Figure 30 and Supplemental Table 37). In *P*. *orientalis*, transcriptional evidence (CPM > 1) was detected for 25.95% (59 240/228 312) of TE-mediated DUPs and PSSDs. However, because only uniquely mapped reads were used, this value is likely an underestimate. Numerous transcribed pseudogenes under positive selection originated from genes involved in reproductive organ development (Supplemental Figure 31). While transcriptional evidence and signals of positive selection do not definitively establish function, they can nonetheless still highlight pseudogenes with potential regulatory capabilities.

### Significant gene fragment capture by TEs

**Figure 7:**
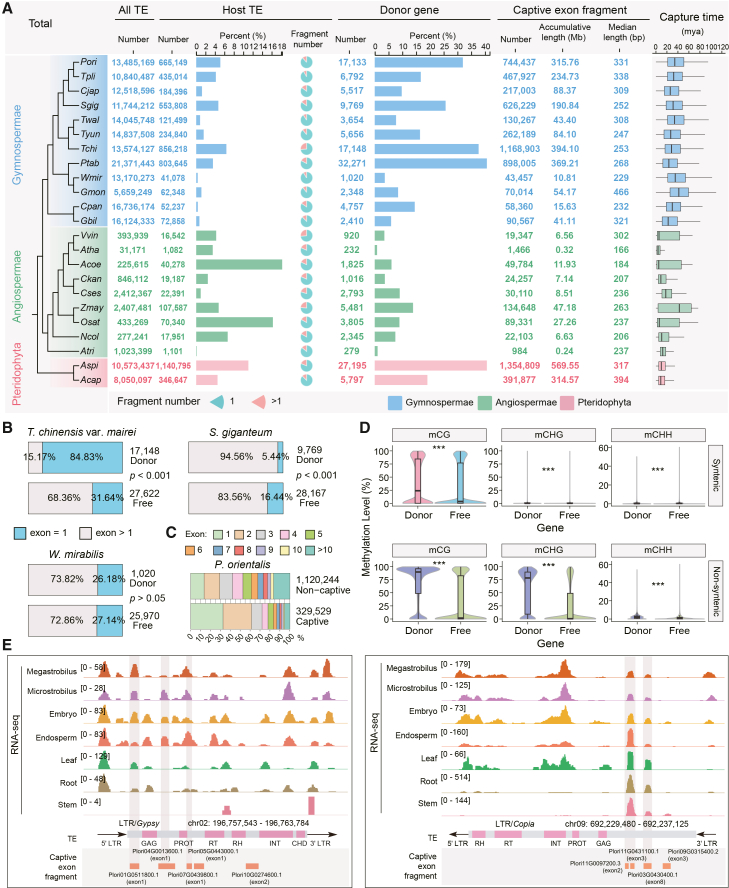
Significant gene capture by TEs. **(A)** Summary of total TEs, host TEs containing captured gene fragments, donor genes involved in gene capture, captured exon fragments, and estimated capture times across 23 representative vascular plants. **(B)** Chi-square test analysis of the proportions of single-exon and multi-exon genes among donor and free genes in *T*. *chinensis* var. *mairei*, *W*. *mirabilis*, and *S*. *giganteum*. **(C)** Chi-square test analysis of the proportions of exon types among captured exons relative to all exon types within multi-exon donor genes in *P*. *orientalis*. **(D)** Distribution of mCG, mCHG, and mCHH methylation levels in the exonic regions of syntenic donor, syntenic free, non-syntenic donor, and non-syntenic free genes. Mann–Whitney U test: ^∗∗∗^*p* < 0.001; NS, not significant. **(E)** Examples of gene capture by *Ty3/Gypsy* and *Ty1/Copia* LTR–RTs in *P*. *orientalis.* LTR, long terminal repeat; GAG, group-specific antigen; PROT, protease; RT, reverse transcriptase; RH, ribonuclease H; INT, integrase; CHD, chromodomain.

Our extensive survey of 23 vascular plant species indicates that gene fragment capture and duplication by TEs (Supplemental Figure 21) have been widespread across vascular plants. Compared with representative angiosperms and pteridophytes, gymnosperms exhibited more ancient gene capture activity (Figure 7A). The incidence of TEs implicated in capture events varied across the phylogeny, with TE content ranging from 0.11% to 17.85% (Supplemental Table 38). Gene fragment capture was implicated in 0.85%–40.09% of coding genes. Such disparities underscore the variable contributions of TEs to the evolution of coding sequences (exons) among different plant lineages. Major TE superfamilies were all found to capture exons (Supplemental Tables 38 and 39). Analysis of host TE content revealed that only a minor fraction (13.68%, on average) of TEs contained two or more captured fragments (Figure 7E), whereas the majority harbored only a single gene fragment (Figure 7A). In conifer genomes, an average of 481 821 TEs (ranging from 121 499 in *T*. *wallichiana* to 856 218 in *T*. *chinensis* var. *mairei*) were found to have captured exonic sequences from an average of 12 243 coding genes (from 3654 genes in *T*. *wallichiana* to 32 271 genes in *P*. *tabuliformis*). In angiosperms, an average of 32 940 TEs captured exonic sequences from an average of 2077 coding genes. However, the intensity of exon capture events—defined as the ratio of the average number of TEs to the average number of genes—was significantly higher in conifers than in angiosperms (39.35 vs. 15.86).

We found interspecific variability in preferences for the capture of donor genes with a single exon versus those with multiple exons (Figure 7B and Supplemental Figure 32). For instance, *T*. *chinensis* var. *mairei* showed a bias toward the capture of single-exon genes, whereas *S*. *giganteum* displayed a preference for multi-exon genes, and *W*. *mirabilis* showed no significant difference. Nonetheless, a uniform preference for the capture of the first and second exons within multi-exon coding genes was evident across all species analyzed (Figure 7C and Supplemental Figure 33). Inter-species Gene Ontology (GO) enrichment analysis of donor genes revealed remarkable functional divergence. This suggests that TEs may exhibit species-specific preferential targeting of functionally distinct genes, thereby facilitating the expansion of genomic coding sequences (Supplemental Figure 34). Echoing the involvement of TEs in gene duplication and pseudogenization, the pronounced overrepresentation of donor genes linked to reproductive organ development GO terms in *P*. *orientalis* suggests a strong association between TE activity and reproductive developmental processes.

Exon methylation characteristics of “donor” genes (i.e., genes from which fragments have been captured) and “free” genes (i.e., genes without any capture events by TEs) were examined in *P*. *orientalis*, revealing a stark contrast: donor genes showed significantly higher methylation levels than free genes, particularly in the mCG and mCHG contexts (Supplemental Figure 35; Mann–Whitney U test, *p* < 0.001). Upon further categorization of donor and free genes into syntenic and non-syntenic subgroups, the distribution patterns of exon methylation levels in syntenic donor genes were largely congruent with those of syntenic free genes, albeit with marginally higher methylation levels in the former (Figure 7D). In contrast, non-syntenic donor genes exhibited a greater proportion of highly methylated exons compared with non-syntenic free genes, with median CG methylation of 89.66% vs. 1.72%, CHG methylation of 78.26% vs. 0.52%, and CHH methylation of 1.94% vs. 0.60% (Mann–Whitney U test, *p* < 0.001 for each comparison). Overall, donor genes generally displayed higher methylation levels than free genes, a pattern that was particularly pronounced in non-syntenic donor genes, where methylation levels were markedly elevated.

Utilizing transcriptomic data across seven tissues of *P*. *orientalis* (Supplemental Figure 36), we quantified donor and free gene expression. No substantial reduction in the expression of syntenic donor genes was observed across tissue types compared with non-syntenic donor genes. However, non-syntenic donor genes exhibited considerably lower expression levels than non-syntenic free genes, consistent with epigenetic regulation associated with TE silencing. Further analysis of TEs revealed no overall reduction in methylation levels in TEs carrying captured gene fragments compared with other TEs in *P*. *orientalis* (Supplemental Figure 37). Notably, 19.81% of gene fragments within TEs showed transcriptional activity (Figure 7E), which was positively correlated with donor gene expression (Spearman rank correlation test; R = 0.34, *p* < 0.001).

## Discussion

### TE-dominated genome expansion among gymnosperms: low removal rates, high methylation levels, and biased accumulation in exons and introns

Plant giga-genomes typically arise through two primary mechanisms: TE proliferation and WGD (Yuan et al., 2022). Size-expanded genomes lacking recent lineage-specific WGDs have been reported in various species and are associated with TE accumulation (Fang et al., 2022; Yuan et al., 2022; Jayakodi et al., 2023). Here, our comparative genomic analysis provides a comprehensive view indicating that genome size expansion in gymnosperms is largely attributable to collective and persistent bursts of transposon activity, particularly the accumulation of RTs (Supplemental Figure 13A). Furthermore, the present study revealed higher rates of LTR–RT removal (*S/I*, *T/I*, and [*S + T*]*/I*) in Gnetales compared with the relatively larger genomes of conifers, cycads, and Ginkgo (Supplemental Figures 14A and 14B), indicating that a relatively faster removal mechanism may counteract TE accumulation in Gnetales than in other gymnosperm lineages. In addition, the high levels of DNA methylation observed in TEs indicate that TE expression is subject to strong suppression. However, the lower levels of methylation observed in expressed TEs and in those associated with accessible chromatin regions also suggest that DNA methylation may not fully suppress TE activity (Figure 4C and 4D). Consequently, genome expansion across gymnosperms may be dynamically influenced by a complex interplay among TE proliferation, TE elimination, and the silencing effects of DNA methylation.

The dynamic activity of TEs, in conjunction with DNA methylation, also significantly influences the exon–intron structure of genes. The present study revealed no significant difference between gymnosperm and angiosperm genomes in the TE fraction within promoter and downstream regions (Figure 3E and Supplemental Table 28). In contrast, the TE fraction in exon and intron regions was markedly higher in gymnosperms, with TEs comprising up to 80% of intronic sequences in conifers. The prevalence or eventual decay of TEs across various genomic regions is influenced by selective pressures and host control, which may vary over extended evolutionary timescales (Krasileva, 2019). As TEs decay over time, insertions near or within genes can continue to influence gene regulation even after the original TE identity has been lost, owing to the conservation of fundamental promoter motifs associated with the original TE (Galindo-González et al., 2017). Subsequently, events that incorporate TE sequences as promoter regulatory elements or as components of gene function may be subject to adaptive selection (Rebollo et al., 2012; Drongitis et al., 2019). Consequently, the co-option of TE-derived sequences has been extensively documented in both plants and animals (Lockton and Gaut, 2009; Huang et al., 2023; Han et al., 2024; Xia et al., 2024), highlighting the evolutionary potential of TE-mediated processes. Furthermore, we found that 88.02% of ultra-long coding genes (>100 kb) containing TE insertions exhibit detectable expression signals in *P*. *orientalis*. Studies on the giga-genomes of Chinese pine (Niu et al., 2022) and lungfish (Wang et al., 2021) have also shown that extraordinarily long introns do not exert detrimental effects on transcription and splicing; rather, larger genes tend to exhibit higher expression levels.

### TE-driven gene duplication and pseudogenization across gymnosperms

Gene duplication provides raw genetic material for evolution and adaptation and is regarded as a primary mechanism facilitating the origination of new genes and driving phenotypic evolution (Wang et al., 2012a). The evolutionary fate of most duplicates is pseudogenization or gene loss, whereas only a small proportion is retained over extended evolutionary periods (Innan and Kondrashov, 2010). Various mechanisms have been proposed to explain the generation of gene duplications (Panchy et al., 2016; Qiao et al., 2019). Polyploidization is a primary source of large-scale gene duplication; however, most gymnosperm genomes (Wan et al., 2022), including *P*. *orientalis*, lack recent whole-genome duplications.

In our analysis of 12 representative gymnosperms, 69.30%–85.15% of coding genes were derived from small-scale duplication events (TD, PD, TRD, and DSD). *K*s analysis revealed a widespread recent burst of small-scale gene duplications in the evolutionary history of gymnosperms (Figure 6B and Supplemental Figure 23). Further investigation indicated that a substantial proportion (approximately 19.59%) of small-scale duplicated genes was derived from TE-mediated duplication events, including retroduplication, TE-capture duplication, and TE-recombination duplication (Supplemental Table 32). A similar role of TEs in duplicate gene formation has also been reported in the TE-rich genomes of *Pinus densiflora*, the Triticeae tribe, and *Paeonia ludlowii* (Wang et al., 2022; Xiao et al., 2023; Jang et al., 2024). We also found that, across the examined gymnosperms, TEs mediated the duplication of 9.41%–39.98% of coding genes, generated an average of 149 229 PSSDs, and accounted for approximately one-fifth of all DUPs (Figure 6F and Supplemental Table 12).

TE-mediated gene duplication and pseudogenization have significantly impacted the dynamics of gene family expansion and contraction, as well as the generation of species-specific genes. The functions of genes involved in these two processes were significantly enriched in various processes related to plant growth and development, as well as responses to biotic and abiotic stresses (Supplemental Tables 34 and 36). For instance, analysis of *P*. *orientalis* revealed that TE-mediated gene duplication events were significantly enriched not only in reproductive development pathways but also in processes related to micronutrient utilization and response (e.g., GO:0019740, GO:0060359, and GO:0010043), cuticular wax biosynthesis and stomatal regulation (e.g., GO:0010025 and GO:0010119), and responses to biotic and abiotic stresses (e.g., GO:0009582, GO:0009611, and GO:0009407). This enrichment pattern suggests that TEs likely contribute to the enhanced drought tolerance, broad adaptability, and ability of *P*. *orientalis* to thrive under nutrient-deficient conditions (Supplemental Table 35). In summary, recent small-scale gene duplications in gymnosperms may have played a role in adaptive evolution comparable to that of WGD, with transposons contributing substantially to gene duplication and pseudogenization within this adaptive evolutionary framework.

The mechanisms by which TEs contribute to the duplication and pseudogenization of entire genes or gene fragments remain incompletely understood. Through an extensive literature survey, we categorized 12 molecular mechanisms of TE-mediated gene duplication into a hierarchical two-domain, four-category framework (Supplemental Figure 38, Supplemental Tables 41–43, and Supplemental Note 5). Domain A includes seven mechanisms directly catalyzed by TE-encoded enzymes (e.g., reverse transcriptases, transposases, and Rep/Hel proteins), whereas domain B includes five mechanisms in which TEs act as recombination substrates or triggers for host cellular machinery (Bailey et al., 2003; Yang et al., 2008; Krasileva, 2019; Qiao et al., 2019; Gozashti et al., 2025). This distinction between TE catalysis and TE facilitation provides a conceptual framework for understanding TE-driven gene duplication on a global scale. Furthermore, end bypass (Tan et al., 2016; Catoni et al., 2019), template switching (Wicker et al., 2010), recurrent transposition, and microhomology guidance (Ma et al., 2023) represent conserved features shared among these diverse mechanisms. The distinction between theoretical comprehensiveness and computational practicality is therefore crucial for interpreting our quantitative findings. We acknowledge that for certain mechanisms (e.g., FoSTeS/MMBIR, fork stalling and template switching/microhomology-mediated break-induced replication), TE-mediated signals cannot be reliably identified based on the features currently used, largely because microhomology is ubiquitous throughout the genome and obscures the distinction between authentic TE-mediated events and stochastic sequence overlaps. Conservative estimates based on detectable mechanisms suggest that the actual contribution of TEs to gene duplication and pseudogenization may exceed the reported values when all theoretical mechanisms are considered. For TE-mediated duplications via recombination substrates, homologous TEs located at homologous tract breakpoints may have undergone sequence divergence during evolution, thereby reducing homology and rendering them undetectable, consistent with previous reports (Zhao et al., 2016; Wang et al., 2022). Moreover, some TEs positioned adjacent to genes may negatively influence gene regulation or expression and may gradually degrade after mediating such events under selective pressures during genome evolution, ultimately losing recognizable transposon characteristics. These factors may lead to an underestimation of the contribution of TEs to gene duplication and pseudogenization.

### Remarkable gene capture by TEs and related epigenetic conflict in gymnosperms

TEs can capture gene fragments, reflecting a significant potential to generate fragment duplications or facilitate the emergence of new genes. This phenomenon has been studied in some animals and plants (Jiang et al., 2004; Zhao et al., 2018; Muyle et al., 2021; Ma et al., 2023) but has not yet been characterized in gymnosperms, which possess abundant TEs. Here, we found that exon capture events in conifers are significantly more frequent than in angiosperms. Capture can occur through RNA-mediated and DNA-mediated processes (Ma et al., 2023), whereby a single transposon may encompass fragments from multiple host genes derived from non-contiguous genomic locations (Figure 7E and Supplemental Table 38). In the present study, our investigation of 23 species revealed that TEs preferentially capture the first and second exons of coding genes (Supplemental Figure 33). The precise processes underlying this specialized mode of capture remain poorly understood and require further in-depth investigation.

Most transposons are typically suppressed by the host’s epigenetic silencing mechanisms under normal conditions, rendering them inactive. In plants, these mechanisms are mediated by small interfering RNAs (siRNAs) that direct RNA interference (RNAi) and RNA-directed DNA methylation (RdDM), targeting homologous sequences at the RNA and DNA levels, respectively (Matzke and Mosher, 2014; Cuerda-Gil and Slotkin, 2016). Therefore, when the host silences TEs containing exon fragments, siRNAs may also target the corresponding donor genes, leading to epigenetic conflicts. In this study, donor genes exhibited significantly higher levels of exon methylation in the mCG and mCHG contexts compared with free genes (Supplemental Figure 35). However, natural selection may reduce the targeting of certain essential genes to maintain their function by moderating the silencing response. For instance, we found that the distribution pattern of exon methylation levels in syntenic donor genes closely parallels that of syntenic free genes, and their expression levels did not exhibit a significant reduction (Figure 7D). These findings align with observations in maize, where epigenetic conflict arises from gene capture by TEs (Muyle et al., 2021).In the present study, methylation levels of TEs harboring captured fragments in *P*. *orientalis* did not show a significant decrease (Supplemental Figure 37), indicating that they did not gain a methylation advantage from capture events. In contrast, TEs harboring captured fragments in maize display a slight advantage (Muyle et al., 2021). On the one hand, epigenetic responses targeting TEs within giga-genomes may persist following capture events, preventing TEs from gaining the advantage of reduced methylation levels. The analyses in the present study were limited to methylation data derived from leaf tissue (vegetative tissue). Future investigations incorporating reproductive tissues may reveal divergent regulatory outcomes due to their distinctive epigenetic patterning. On the other hand, it will be important to investigate the effects of epigenetic conflict arising from TE-mediated gene capture across a broader range of species, both on host genes and on transposons.

Our comparative analysis across multiple species systematically elucidates the intricate dynamics between transposons and coding gene space in gymnosperms, revealing patterns and principles underlying the large size and complexity of gymnosperm genomes. These findings provide important reference cases for studying the role of transposons in plant genome evolution. The high-quality chromosome-level genome of the cypress *P*. *orientalis* further enhances our understanding of the genomic characteristics of conifers and provides valuable resources for genomic research and genetic improvement of these species.

## Materials and methods

### Plant materials and data collection

To perform whole-genome assembly, a mature, healthy, approximately 30-year-old clonally propagated *P*. *orientalis* elite tree (located at block 2, plot 2, row 54, column 22, with elite clone number 153) was selected from the seed orchard at the National Tree Breeding Station for *P*. *orientalis* in Jiaxian County, Henan Province, China (33°48′00″–34°11′50″N, 113°01′45″–113°24′50″E). A high-density genetic map had previously been generated from a megagametophyte population derived from the same individual sequenced in this study (Jin et al., 2019).

To obtain the whole-genome sequence, fresh leaves were harvested for sequencing on multiple platforms. To assist gene annotation, leaf samples from the same individual were collected for ONT full-length transcriptome sequencing (Supplemental Table 1). Additionally, Illumina RNA sequencing (RNA-seq) data from different tissues and treatments of other *P*. *orientalis* individuals were obtained from the NCBI SRA database (Supplemental Table 6).

To quantify the expression of genomic elements, embryo and megagametophyte tissues from seeds produced by the sequenced individual, as well as leaf, megastrobilus, and microstrobilus tissues collected from natural populations, were subjected to transcriptome sequencing, with three biological replicates for each tissue type. In all cases, fresh tissues were harvested and immediately frozen in liquid nitrogen. In addition, six samples of young root and young stem tissues of *P*. *orientalis* were obtained from the SRA database to complement the gene and transposon expression data (Supplemental Table 8). Seed embryo, seed megagametophyte, leaf from one-year-old seedlings, and microstrobilus tissues were also subjected to assay for transposase-accessible chromatin sequencing (ATAC-seq). Furthermore, leaves from the genome-sequenced individual were used for bisulfite sequencing for genome-wide DNA methylation profiling.

### Library construction and sequencing

Libraries with ∼20-kb inserts were prepared and sequenced on the ONT GridION and PacBio Sequel II SMRT platforms. For Illumina short-read sequencing, PCR-free libraries with 150-bp paired-end inserts were constructed and sequenced on the Illumina HiSeq platform. DNA fragments were end-repaired, adaptor-ligated, PCR-amplified, and converted into paired-end sequencing libraries for sequencing on the Illumina HiSeq platform.

Total RNA was used for cDNA library construction following the ONT protocol and sequenced on the PromethION platform to obtain ONT full-length transcriptome data. For Illumina RNA sequencing, libraries were prepared using the NEBNext Ultra RNA Library Prep Kit and sequenced on the Illumina HiSeq platform in 150-bp paired-end mode. For ATAC-seq, transposition reaction products were purified and sequenced on the Illumina HiSeq platform. For WGBS, extracted DNA was treated with the EZ DNA Methylation-Gold Kit (Zymo Research) prior to library preparation and subsequently sequenced on the Illumina NovaSeq platform.

More detailed information on sequencing procedures is provided in Supplemental Note 1.

### Genome assembly and quality assessment

To determine the genome characteristics of *P*. *orientalis*, *k*-mer analysis was performed using Jellyfish v2.2.8 (Marçais and Kingsford, 2011) and GCE v1.0.2 (https://github.com/fanagislab/GCE). Canu v1.7 (Koren et al., 2017), NextDenovo v2.3.0 (Hu et al., 2024), and SmartDenovo (Liu et al., 2021a) were employed for the correction and initial assembly of the ONT long reads, followed by three rounds of polishing using the Arrow algorithm (Chin et al., 2013) based on PacBio reads. High-quality Illumina short reads were then aligned to the corrected Nanopore contigs, and NextPolish v1.0.0 (Hu et al., 2020) was used to improve single-base accuracy in the contigs through four rounds of polishing. The Hi-C data were aligned to the primary assembly to generate a normalized contact matrix using Juicer v1.7.6 (https://github.com/aidenlab/juicer). The Hi-C contact matrix was then used for scaffolding with the 3D-DNA pipeline. The resulting scaffolds underwent manual refinement to correct errors in insertions, junctions, order, and mis-joins. Subsequently, gaps in the scaffolds were filled through one iteration using LR_Gapcloser v1.0 (https://github.com/CAFS-bioinformatics/LR_Gapcloser) based on Nanopore reads, followed by four rounds of polishing with NextPolish based on Illumina reads. Redundant unanchored contigs were removed using Redundans v0.13c (identity > 0.98) (Pryszcz and Gabaldon, 2016). More details regarding genome assembly can be found in Supplemental Note 2.

The quality of the genome assembly was evaluated using complementary methods. First, the assembly quality of finalized gymnosperm genome assemblies was assessed using a custom Python script (https://github.com/baoyutao1/asssembly_statistic/blob/main/asssembly_statistic.py), with comparative analysis based on contig N50 metrics. Genome completeness and continuity were then evaluated by identifying conserved plant genes using Compleasm v0.2.2 (Huang and Li, 2023). To estimate consensus error rates and mapping rates, preprocessed reads from ONT, PacBio, Illumina, RNA-seq, and Hi-C datasets were aligned to the final assembly using BWA v0.7 (https://github.com/lh3/bwa), Minimap2 v2.17 (Li, 2018), and HISAT2 v2.2.1 (Kim et al., 2015). Merqury v1.3 (Rhie et al., 2020) was also used to evaluate genome quality. Furthermore, genetic map markers (Jin et al., 2019) generated from the megagametophyte population derived from the genome-sequenced individual were mapped to the assembled genome using Chromonomer v1.07 (https://github.com/jleluyer/chromonomer_workflow).

### Gene prediction and functional annotation

To obtain protein homology evidence for the structural annotation of coding genes, an integrated dataset of 146 741 non-redundant protein sequences from four related gymnosperms was compiled using CD-HIT v4.8.1 (Fu et al., 2012). For transcriptome-based evidence, *de novo* and reference genome–based transcripts were constructed using both ONT full-length and Illumina transcriptome sequencing reads in conjunction with HISAT2 v2.2.1 (Kim et al., 2015), Trinity v2.13.2 (Grabherr et al., 2011), StringTie v2.2.0 (Pertea et al., 2015), and Minimap2 v2.17 (Li, 2018). Subsequently, these transcripts were integrated with previously published data (Hu et al., 2016). The resulting collection was merged and subjected to redundancy removal using CD-HIT v4.8.1, yielding a final set of 414 319 unique transcript sequences.

The gene structure was first annotated using the PASA v2.5.0 pipeline (Haas et al., 2003), relying on transcriptional evidence. High-quality open reading frame (ORF) structures produced by PASA v2.5.0 were used to train the *ab initio* parameter model in AUGUSTUS v3.4.0 (Stanke et al., 2008). Subsequently, the MAKER2 pipeline (Holt and Yandell, 2011) was applied to the repeat-masked assembly generated with RepeatModeler (Flynn et al., 2020) and RepeatMasker (http://www.repeatmasker.org), implementing a combined strategy of *ab initio*, transcriptome-, and homology-based gene prediction. BUSCO v4.1.2 (Simão et al., 2015) was also executed. To enhance annotation accuracy, coding gene predictions derived from the PASA, MAKER2, and BUSCO pipelines were integrated and updated using EVidenceModeler (EVM) v1.1.1 (Haas et al., 2008). Subsequently, transcripts and associated genes with TE domain coverage exceeding 30% (identified using TEsorter with parameters: -cov 30 -eval 1e−10) (Zhang et al., 2022a), as well as genes with excessively short annotations (≤50 amino acids), were filtered out, ultimately yielding a high-confidence gene set. The final coding gene annotation retained only the longest transcript isoform for each gene and was assessed for completeness using BUSCO v4.1.2 with embryophyta_odb10 in protein mode and the OMArk v0.3.0 (Nevers et al., 2024) webserver (https://omark.omabrowser.org/; OMAmer database v2.0.0) with the ancestral clade Spermatophyta.

In addition, rRNA, tRNA, and other noncoding RNAs were annotated using Barrnap (https://github.com/tseemann/barrnap), tRNAscan-SE v2.0 (Lowe and Eddy, 1997), and RfamScan v9.1 (http://eggnogdb.embl.de), respectively.

Three strategies were employed for the functional annotation of protein-coding genes in this study. First, eggNOG-mapper (Huerta-Cepas et al., 2017) (--target_taxa Viridiplantae) was used to map gene sequences. Second, homology and similarity searches were performed against orthologous gene databases using BLASTP with an E-value cutoff of 1e−05 and a minimum identity of 30%. Third, conserved amino acid sequences, motifs, and domains were identified through domain similarity prediction using InterProScan v5.14–53.0 (http://www.ebi.ac.uk/InterProScan).

Further information on gene prediction and functional annotation can be found in Supplemental Note 3.

### Gene family, phylogenomic, and genomic synteny analyses

Representative vascular species (Supplemental Table 16) were selected to construct orthogroups using OrthoFinder v2.3.1 (Emms and Kelly, 2019). The longest transcript was selected for each coding gene. Phylogenetic reconstruction was performed based on 692 low-copy gene families. Each selected gene family met the following criterion: at least 80% of the species contained a single-copy ortholog, whereas the remaining species either lacked the gene or possessed low-copy orthologs. For cases involving low-copy orthologs, the gene copy with the shortest branch length was selected as the representative sequence for tree construction. This strategy prioritizes slowly evolving orthologs under strong purifying selection to minimize phylogenetic artifacts (e.g., long-branch attraction) and ensure an accurate representation of the species tree (Kapli et al., 2020). Nucleotide sequences from the 692 single-copy orthologs were aligned using MUSCLE v3.8.3.1 (Edgar, 2004). The alignments were concatenated and trimmed using trimAl v1.2 (Capella-Gutiérrez et al., 2009), and a maximum likelihood phylogenetic species tree was constructed using IQ-TREE v1.6.7 (Nguyen et al., 2015) with 1000 bootstrap replicates and the best-fit model JTT+F+R6. Concatenation-based approaches inferred species tree topologies with high bootstrap support. Four-fold degenerate (4d) sites were extracted from the gene sequences retained in the previous step. Divergence times among individual species were estimated using the MCMCTree module in PAML (Yang, 2007) based on the 4d sites, whole coding sequences (CDS), and the species tree. Calibration points were obtained from the TimeTree database (http://timetree.org/). The divergence time estimates derived from the 4d sites and the whole coding sequences were highly consistent (Spearman rank correlation test, R = 0.98, *p* < 0.001) (Supplemental Figure 7). Using CAFÉ v5.0 (Mendes et al., 2021), we further inferred the expansion, contraction, and species specificity of gene families across extant species and their last common ancestors based on most recent common ancestor (MRCA) analysis. Tree visualization was performed using tvBOT (Xie et al., 2023).

To investigate potential WGD events in *P*. *orientalis*, we assessed the distribution of synonymous substitutions per site (*K*s) among paralogs. Protein sequences from all species were aligned pairwise using DIAMOND v2.0.2 (Buchfink et al., 2015) with the BLASTP algorithm. Collinear paralog pairs in syntenic regions were identified using MCScanX (Wang et al., 2012b). ParaAT v2.0 (Zhang et al., 2012) was used to generate multiple protein-coding DNA alignments, and KaKs_Calculator v2.0 (Wang et al., 2010) was used to calculate *K*s values for each paralog pair. Additionally, WGDI (Sun et al., 2022) was employed to sequentially execute the parameters -icl, -ks, -bi, -c, -bk, and -kp to identify syntenic blocks and syntenic genes. Tree2GD v1.0.43 (Chen et al., 2022) was also used to confirm the occurrence of WGD events in gymnosperms. Syntenic gene pairs between *P*. *orientalis* and ten gymnosperm species were identified using JCVI v0.84, the Python version of MCScan (Tang et al., 2008) (https://github.com/tanghaibao/jcvi/wiki/MCscan-(Python-version)). Additional details are provided in Supplemental Note 4.

### TE annotation and comparison

Species-specific TE libraries for *P*. *orientalis* and 22 other representative vascular plants were systematically constructed by performing extensive *de novo* TE annotation using the EDTA v1.9.4 (Ou et al., 2019) and DeepTE (Yan et al., 2020) workflows (Supplemental Figure 5), facilitating genome-wide TE annotation (Supplemental Table 14). Specifically, LTR_Finder v1.07 (Xu and Wang, 2007), LTRharvest v1.5.10 (Ellinghaus et al., 2008), and LTR_retriever v2.9.0 (Ou and Jiang, 2018) were used to identify LTR retrotransposons. Terminal inverted repeat (TIR) transposons were detected using Generic Repeat Finder v1.0 (Shi and Liang, 2019) and TIR-Learner v1.23 (Su et al., 2019), while HelitronScanner v1.0 (Xiong et al., 2014) was used to identify Helitron elements. After obtaining a raw library through initial filtering, the Pine Interspersed Element Resource database (PIER2.0) (Wegrzyn et al., 2014) was incorporated specifically for gymnosperm species, and RepeatModeler (Flynn et al., 2020) was utilized to identify TEs (such as short interspersed nuclear elements [SINEs] and Long Interspersed Nuclear Elements [LINEs]) potentially missed by structure-based identification methods. CDS sequences were then filtered out, and the results were merged to generate a TE library. This library was then used as input for DeepTE with the plant model to categorize previously unclassified repetitive elements, ultimately generating the final TE library. RepeatMasker was used to annotate fragmented TEs by detecting homology to structurally characterized transposons. The locations of solo LTRs were extracted from the homologous annotation results using the find_LTR.pl and solo_finder.pl scripts (https://github.com/oushujun/PopTEvo/blob/main/TE_annotation/bin). After calculating the lengths of intact (*I*), solo (*S*; solo-LTRs, remnants of transposons deleted via unequal or illegitimate homologous recombination), and truncated (*T*; generated through partial deletion) LTR–RTs, the summed total of *I* + *S + T* was used as an approximate proxy to estimate the proliferation levels of LTR–RTs (Lyu et al., 2018). Furthermore, the ratios of solo-LTRs and truncated elements to intact LTR–RTs (*S/I*, *T/I*, and [*S + T*]*/I*) were evaluated to assess LTR–RT removal rates.

LTR–RT candidates were also predicted *de novo* using LTRharvest (Ellinghaus et al., 2008) and LTRdigest (Steinbiss et al., 2009) based on the following criteria (Zhou et al., 2021): a pair of LTR sequences originating from a candidate LTR–RT must be positioned within an interval of less than 15 kb and exhibit a similarity of at least 80%, and the length of each individual LTR sequence must range from 100 to 3000 bp. Subsequently, the internal sequences of all candidate LTR–RTs were annotated by aligning their Gag–Pol protein sequences against the 80 plant-specific TE protein domain reference dataset (REXdb, Viridiplantae v3.0) (Neumann et al., 2019). Alignment was conducted using LAST v983 (Kiełbasa et al., 2011) with the specified parameters: “-L 10 -m 70 -P BL80 -e 80.” LTR–RTs harboring alignments with the domains “GAG” (capsid protein), “AP” (aspartic proteinase), “INT” (integrase), “RT” (reverse transcriptase), and “RH” (RNase H) were defined as intact LTR–RTs (*I*).

The TE identification results from EDTA were ultimately combined with the intact LTR–RT (*I*) annotations, and redundancies were removed to generate genome-wide TE coordinate annotations for all 23 species (Supplemental Figure 5 and Supplemental Table 14). An alternative annotation of *Helitron* elements was generated using HELIANO (Li et al., 2024), a recently released method. The pic function in the R package ape (Paradis and Schliep, 2019) was used to compute phylogenetic independent contrasts (PICs), followed by linear regression analysis of the calculated PIC values.

Protein-coding sequences annotated from 23 species (Supplemental Table 14) were aligned against REXdb (Viridiplantae v3.0) (Neumann et al., 2019) using BLASTP (-evalue 1e−5 -max_target_seqs 5). Genes with high sequence similarity to TE protein domains (identity ≥30% and sequence coverage ≥80%) were defined as putative TE-associated coding genes (Supplemental Table 40). These TE-associated genes were excluded from subsequent analyses of TE-mediated gene duplication and pseudogenization.

### Kimura distance-based TE age distribution and phylogenetic analysis

The insertion time of intact LTR–RTs was estimated based on the divergence between the 5′ and 3′ LTRs of the same element. The LTRs were aligned using MAFFT v7.221 (Katoh and Standley, 2013), and insertion time was calculated using the equation T = *K*/2r, where r is the mutation rate per site per year. The Kimura two-parameter method (Kimura, 1980) was used to estimate the divergence (*K*) between paired LTRs for each intact LTR–RT.

Furthermore, extensive analyses were conducted to examine the amplification histories of all TEs in detail. The Kimura genetic distance between each annotated TE copy and the consensus sequence of its respective TE family was calculated using the specialized helper script calcDivergenceFromAlign.pl provided in the RepeatMasker 4.0.5 package, which accounts for both transitional and transversional substitution rates. TE age was then estimated using the equation T = *K*/r.

Different transposon nucleotide substitution rates were applied for different plant groups in this study: 2.2 × 10⁻⁹ substitutions per site per year for gymnosperms (Nystedt et al., 2013), 1.3 × 10⁻⁸ for monocots (Ma et al., 2004), 6.5 × 10⁻⁹ for basal angiosperms (Amborella et al., 2013), 1.5 × 10⁻⁸ for eudicots (Koch et al., 2000), 6.04 × 10⁻⁹ for magnoliids (Cui et al., 2006), 3.8 × 10⁻⁹ for Chloranthales (Ma et al., 2021), and 4.79 × 10⁻⁹ for *Alsophila spinulosa* and *Adiantum capillus-veneris* (Baniaga and Barker, 2019).

To further investigate the evolution of gymnosperm LTR–RTs, phylogenetic analyses were conducted on the amino acid sequences of reverse transcriptase domains from intact *Ty3/Gypsy* and *Ty1/Copia* retrotransposons across 8 representative species. Two hundred sequences were randomly sampled from each species, with the random seed set to 100. Multiple sequence alignment was performed using MUSCLE v3.8.3.1 (Edgar, 2004), after which a neighbor-joining phylogenetic tree was constructed using IQ-TREE v1.6.7 (Nguyen et al., 2015). The results were visualized using ChiPlot (https://www.chiplot.online/).

### Gene duplication

DupGen_finder (Qiao et al., 2019) (https://github.com/qiao-xin/DupGen_finder) was executed with default parameters to identify duplicated genes. An all-versus-all BLAST search of protein sequences was performed using DIAMOND, after which gene coordinates (from the GFF3 file) were integrated to determine the duplication modes of paralogous gene pairs. The identified duplication modes included WGD or SD, TD, PD, TRD, and DSD. KaKs_Calculator v2.0 was then used to estimate *K*s (synonymous substitutions per site), *K*a (nonsynonymous substitutions per site), and *K*a/*K*s ratios for each duplicated pair. Only gene pairs with *K*s ≤ 3 were retained for downstream analyses.

Although numerous mechanisms by which TEs contribute to gene duplication and pseudogenization have been identified across organisms, establishing a comprehensive theoretical framework and developing practical computational approaches for large-scale genomic analyses remain major challenges. Through an extensive literature survey, we identified 12 molecular mechanisms underlying TE-mediated gene duplication across diverse species and organized them into a hierarchical framework comprising two domains and four categories (Supplemental Figure 38 and Supplemental Note 5). Domain A (direct TE-catalyzed mechanisms) includes seven pathways driven by TE-encoded enzymes. It is subdivided into category 1 (RNA-mediated duplication), encompassing retrogenesis (Mech. 1), RT transduction (Mech. 2), and RT template switching (Mech. 3), and category 2 (direct DNA transposon-mediated capture), which includes Pack-type transposons (Mech. 4), Helitrons (Mech. 5), composite transposons (Mech. 6), and terminal bypass events in TIR transposons (Mech. 7). Domain B (TE-facilitated mechanisms) comprises five mechanisms in which TEs act as homologous substrates or structural triggers for host cellular machinery. This domain is further categorized into category 3 (homology-directed recombination/repair), featuring non-allelic homologous recombination (NAHR; Mech. 8), LTR unequal recombination (Mech. 9), and synthesis-dependent strand annealing (SDSA; Mech. 10), and category 4 (replication-based mechanisms), including FoSTeS/MMBIR (Mech. 11) and FoSTeS + transposition (FoSTeST; Mech. 12). Additional details are provided in Supplemental Note 5.

To ensure computational feasibility for large-scale genomic analyses, we organized the identification of TE-mediated gene duplications into three components by synthesizing common and detectable genomic signatures shared across diverse mechanisms (Figure 5A). Part 1 (retroduplication, encompassing Mechs. 1–3) is characterized by intronless gene copies derived from reverse transcription. Part 2 (TE-capture duplication, linked to Mechs. 2–7 and 12, and partially to Mech. 11) involves instances in which TEs contain captured gene fragments. Part 3 (TE-recombination duplication, corresponding to Mechs. 8–10) is identified by homologous TE sequences located at recombination breakpoints. These categories represent the computationally tractable subset of the broader theoretical framework. Our identification pipeline used genome sequences, gene annotations (excluding TE-related genes), TE annotations, and homologous gene pairs (excluding those derived from WGDs or SDs) as input data.

Retroduplication events (Part 1) were identified by screening for characteristic intron-loss signatures. Homologous gene pairs consisting of a multi-exon parental gene and a single-exon derivative were first extracted, with candidate single-exon genes required to span at least two exon–exon junctions of the parental locus. Subsequently, a stringent pairwise similarity filter was applied based on mRNA/protein best bitscore hits (identity ≥70%) to obtain the most likely parental gene. Finally, flanking sequences were examined for hallmarks of retrotransposition; candidates exhibiting both poly(A) tails and TSDs were classified as canonical retrogenes, whereas those lacking these structural features were categorized as non-canonical retrogenes.

TE-capture duplications (Part 2) focused on genes physically harbored within TEs. First, structurally intact TEs, including both autonomous and non-autonomous elements, were identified by validating insertion-related structural features such as TSDs. Subsequently, the spatial relationships between genes and these TEs were evaluated using bedtools intersect (-f 1). Genes located entirely within the boundaries of an intact TE were designated as “captive genes.” Finally, these candidates were subjected to pairwise similarity filtering (protein level; best bitscore hits; ≥70% identity) to retain the most likely paralogous pairs.

To identify duplications resulting from TE-mediated recombination (Part 3), homology searches were progressively extended across the genic regions and upstream and downstream flanking regions of duplicate gene pairs categorized into the four small-scale duplication modes (TD, PD, TRD, and DSD) using MUMmer v4.0 (nucmer –maxmatch; delta-filter −1) (Marçais et al., 2018). Searches were terminated once homology was no longer detectable, thereby defining the breakpoints of homologous tracts (Figure 5B). Non-homologous spacers ≤1 kb between contiguous homologous sequences were permitted. At each identified breakpoint, 1 kb flanking regions were screened for homologous TE sequence pairs with ≥80% identity and ≥80% coverage using BLASTN (-evalue 1e−10). Duplicate gene pairs exhibiting homologous TE sequences at or immediately adjacent to the breakpoints of homologous tracts were classified as TE-mediated recombination gene duplications. To account for potential confounding effects arising from the high TE density of the genome (>60% TE content), a randomization test was conducted to determine whether the observed enrichment of homologous TE sequences near the breakpoints of homologous tracts in duplicate gene pairs differed significantly from the genomic background, as assessed using randomly selected gene pairs (Figure 5C). In this analysis, duplicated genes belonging to the same duplication category were randomly paired and searched for homologous TE sequences using the same criteria described above.

The gene duplication events identified through the integrated application of the three strategies were classified as TE-mediated gene duplications. Additionally, the detected duplicated genes were categorized as significantly expanded, significantly contracted, or species-specific genes to evaluate the contributions of TE-mediated gene duplications to each category.

### Identification of gene capture by TEs and estimation of capture time

TE-mediated capture of coding gene fragments was identified using the approach described in a previous study (Muyle et al., 2021). All physical overlaps between coding genes and TEs were first excluded, followed by a BLASTN homology search between the exons of the longest transcript of each gene and the TE regions identified in each genome studied. A stringent E-value cutoff of 1e−40 was applied to minimize false positives. In cases where TE overlap occurred between exons of multiple genes, the highest BLASTN bit score was used to determine the true donor gene. If multiple genes exhibited identical bit scores, they were considered true donors and retained. The 80 plant TE protein domain reference dataset from RepeatExplorer (http://repeatexplorer.org/?page_id=918) (Viridiplantae v3.0) (Neumann et al., 2019) was further used via BLASTX (-evalue 1e−10 -max_target_seqs 5) to remove captured fragments aligning to TE-derived domains, thereby reducing false positives. The final TE-captured gene fragment datasets across 23 species obtained using the same pipeline were then compared and analyzed.

*K*s values between donor genes and the captured fragments within TEs were calculated using MACSE v2, which provides an automated solution for aligning protein-coding datasets containing non-functional sequences without disrupting codon structure (Ranwez et al., 2018). In cases where stop codons were detected in fragments, they were replaced with “NNN” using the yn00 program in the PAML package (Yang, 2007). To estimate capture age, *K*s values were divided by 2r. The nucleotide substitution rates (r) used for each species were consistent with those applied in the TE age distribution calculations described above.

### Pseudogene annotation

Pseudogene identification and annotation of chromosomal locations, sequences, and source coding genes were performed on the genome with TEs masked using the PseudoPipe (Zhang et al., 2006) pipeline under default parameters. Stringent quality control criteria were applied to pseudogene identification. For DUPs, PSSDs, and pseudogenic fragments, the following cutoffs had to be met: greater than 40% amino acid sequence identity between the pseudogene and its source gene, an E-value ≤ 1e−10, and coverage of at least 70% of the pseudogene sequence by the source gene for the first two pseudogene types. Additional filtering was performed to remove candidate regions overlapping with captured gene fragments, resulting in the final pseudogene dataset.

To identify TE-mediated pseudogenization events among DUPs (where the source gene did not include TE-associated genes), we employed an approach similar to that used for identifying TE-mediated recombination gene duplications (Figure 5B). Homology surveys were progressively extended across the genic, upstream, and downstream flanking regions of the source–pseudogene gene pairs using MUMmer (nucmer --maxmatch, delta-filter −1). After identifying homologous tracts, 1 kb regions flanking the breakpoints were screened using BLASTN (-evalue 1e−10) to identify homologous TE sequence pairs with ≥80% identity and ≥80% coverage. Pseudogene–source gene pairs exhibiting homologous TE sequences at or immediately adjacent to the breakpoints of homologous tracts were classified as products of TE-mediated pseudogenization (Supplemental Figure 29). Furthermore, a randomization test was performed to assess whether the occurrence of homologous TE sequences within 1 kb of breakpoints flanking homologous tracts in pseudogene–source gene pairs differed significantly from the genomic background, using randomly matched gene–pseudogene pairs as controls (Figure 5C). Source genes and DUPs were randomly paired 1000 times to detect the presence of homologous TE sequences between any pair. In addition, source genes were categorized into significantly expanded, significantly contracted, and species-specific groups, and we examined whether these three groups exhibited different patterns of TE-mediated pseudogenization.

### RNA-seq, ATAC-seq, and WGBS data analyses

Raw RNA-seq reads were filtered using Fastp v0.21.0 (Chen et al., 2018) (Supplemental Table 8). Clean reads were aligned to the reference genome using STAR v2.7.8a (Dobin et al., 2013) with the parameters “--outFilterMultimapNmax 10 000 --winAnchorMultimapNmax 10 000.” Reads mapping to TEs in *P*. *orientalis* were counted using the featureCounts function in the Rsubread (Liao et al., 2019) R package with the parameter “countMultiMappingReads = TRUE,” following a previous study (Teissandier et al., 2019). Custom R scripts were used to calculate CPM values for TEs and TPM values for genes, representing the expression levels of each element. Given the high degree of sequence homology between pseudogenes and their cognate coding genes, only uniquely mapped reads were used in the quantification process for calculating CPM values, thereby minimizing potential bias. The tissue-specific expression of elements across 7 tissues was evaluated by calculating the tau index using TBtools (Chen et al., 2023).

Raw reads from the ATAC-seq assay were filtered using FastQC v0.11.9 (https://www.bioinformatics.babraham.ac.uk/projects/fastqc/) and Trimmomatic v0.39 (Bolger et al., 2014). Clean reads were aligned to the genome using BWA v0.7 (https://github.com/lh3/bwa) with the parameters “-M -R” (Supplemental Table 8). PCR duplicates were removed using Picard v2.23.9 (https://github.com/broadinstitute/picard), and low-quality reads (unmatched reads and those with mapping quality score [MAPQ] <20) were filtered using SAMtools v1.10 (Li et al., 2009). The BAM files were then converted to BigWig files using deepTools v3.1.3 (Ramírez et al., 2014). The distribution of reads from the transcriptome and ATAC-seq data across the *P*. *orientalis* genome was visualized using IGV v2.4.13 (Thorvaldsdóttir et al., 2013). High-quality clean reads were then analyzed using MACS2 v2.2.7.1 (Zhang et al., 2008) with the parameters “--nomodel --shift -100 --extsize 200 -q 0.01.” Accessible chromatin regions (ACRs) were obtained by merging all ACRs detected in the four tissues according to genomic coordinates using the merge function in BEDTools (Figure 1A). Overall, 38.17% of ACRs were located in promoter regions, 44.44% in distal intergenic regions, 10.98% in exonic regions (including 5′ UTRs and 3′ UTRs), 4.12% in intronic regions, and 2.29% in downstream regions (Figure 4B). The overlap between peaks and TEs was examined using the intersect subcommand (-f 0.1). Using the PlantRegMap database (Tian et al., 2020), we consolidated transcription factor binding motif information from *A. thaliana*, *Picea abies*, and *Pseudotsuga menziesii*. This information, together with the FIMO tool (Grant et al., 2011) from the MEME Suite, was used to predict transcription factor binding sites within TE-containing ACR regions in *P*. *orientalis*, with results filtered based on a *q* value <0.05.

Raw reads from WGBS were mapped to the *P*. *orientalis* genome using Bismark v0.20.0 (Krueger and Andrews, 2011). Across the 11 chromosomes, the coverage and average depth were approximately 98.77% and 42×, respectively (Supplemental Table 29). Methylated cytosines (Cs) were called from uniquely mapped reads using BatMeth2 (Lim et al., 2012). Methylation ratios for cytosines covered by ≥4 reads were calculated as the number of Cs divided by the sum of Cs and Ts and were used for subsequent analyses of gene and TE methylation patterns.

### Gene functional enrichment

Hypergeometric tests were conducted to determine whether specific GO functional categories were significantly overrepresented among the target gene sets. Functional enrichment analysis was performed using the clusterProfiler R package v4.6.2 (Wu et al., 2021). First, protein sequences of the target species were uploaded to the EggNOG–Mapper web server (http://eggnog-mapper.embl.de/) for annotation. The R script emapperx.R (http://git.genek.cn:3333/zhxd2/emcp) was then used, together with the annotation results, to construct an organism-specific OrgDB package. GO enrichment analysis was performed using the enrichGO function (ont = “BP”, pAdjustMethod = “BH”). Redundant GO terms were removed using the simplify function (cutoff, by = “pvalue”, select_fun = min) or by clustering terms with the aPEAR package (Kerseviciute and Gordevicius, 2023).

## Data and code availability

The raw sequencing reads for the genome, transcriptome, ATAC-seq, and methylome data generated in this study have been deposited in the Genome Sequence Archive (GSA) at the National Genomics Data Center (NGDC), China National Center for Bioinformation/Beijing Institute of Genomics, Chinese Academy of Sciences (accession number: CRA018817), and are publicly accessible at https://ngdc.cncb.ac.cn/gsa. The whole-genome sequence data for *P*. *orientalis* have also been deposited in the Genome Warehouse (GWH) at NGDC under accession number GWHFIIO00000000.1 and are publicly accessible at https://ngdc.cncb.ac.cn/gwh. The genome assembly and annotation are additionally available on Figshare (https://doi.org/10.6084/m9.figshare.28005944) and Zenodo (https://zenodo.org/records/13734625). Furthermore, the TE annotations, pseudogene annotations, and custom scripts used to identify TE-mediated gene duplications in this study are available on Zenodo (https://zenodo.org/records/13741567).

## Funding

This study was supported by the 10.13039/501100012166National Key Research and Development Program of China (2022YFD2200103-02), the 10.13039/501100001809National Natural Science Foundation of China (32171816), the 10.13039/501100017700Henan Province Science and Technology Research Project (232102110193), and T4F Sweden.

## Acknowledgments

The authors declare no competing interests. This work was partially supported by the Wallenberg Initiatives in Forest Research (WIFORCE) funded by the Knut and Alice Wallenberg Foundation.

## Author contributions

J.-F.M. and W.Z. conceived and designed the study. Y.-T.B., Z.-C.L., S.-Q.J., H.L., K.-H.J., S.-S.Z., S.N., X.-M.Y., T.-L.S., X.-C.T., S.-W.Z., Z.-Y.C., H.-Y.M., R.-G.Z., and X.-L.Y. prepared the materials, conducted the experiments, analyzed the data, and compiled the results. Y.-T.B. drafted the manuscript. C.C., Y.A.E.-K., I.P., X.-R.W., J.-F.M., and W.Z. contributed to data interpretation and finalization of the manuscript draft. All authors reviewed and approved the final version of the manuscript.

## Supplemental information

Document S1. Supplemental Figures 1–38 and Supplemental Notes 1–5

Document S2. Supplemental Tables 1–43

Document S3. Article plus supplemental information

## References

[bib1] Ahuja M.R., Neale D.B. (2005). Evolution of genome size in conifers. Silvae Genet..

[bib2] Almojil D., Bourgeois Y., Falis M., Hariyani I., Wilcox J., Boissinot S. (2021). The structural, functional and evolutionary impact of transposable elements in eukaryotes. Genes.

[bib3] Amborella G.P., Albert V.A., Barbazuk W.B., dePamphilis C.W., Der J.P., Leebens-Mack J., Ma H., Palmer J.D., Rounsley S., Sankoff D. (2013). The *Amborella* genome and the evolution of flowering plants. Science.

[bib4] Ausin I., Feng S., Yu C., Liu W., Kuo H.Y., Jacobsen E.L., Zhai J., Gallego-Bartolome J., Wang L., Egertsdotter U. (2016). DNA methylome of the 20-gigabase Norway spruce genome. Proc. Natl. Acad. Sci. USA.

[bib5] Bailey J.A., Liu G., Eichler E.E. (2003). An *Alu* transposition model for the origin and expansion of human segmental duplications. Am. J. Hum. Genet..

[bib6] Baniaga A.E., Barker M.S. (2019). Nuclear genome size is positively correlated with median LTR-RT insertion time in fern and lycophyte genomes. Am. Fern J..

[bib7] Beric A., Mabry M.E., Harkess A.E., Brose J., Schranz M.E., Conant G.C., Edger P.P., Meyers B.C., Pires J.C. (2021). Comparative phylogenetics of repetitive elements in a diverse order of flowering plants (Brassicales). G3 (Bethesda)..

[bib8] Bolger A.M., Lohse M., Usadel B. (2014). Trimmomatic: a flexible trimmer for Illumina sequence data. Bioinformatics.

[bib9] Buchfink B., Xie C., Huson D.H. (2015). Fast and sensitive protein alignment using DIAMOND. Nat. Methods.

[bib10] Butelli E., Licciardello C., Zhang Y., Liu J., Mackay S., Bailey P., Reforgiato-Recupero G., Martin C. (2012). Retrotransposons control fruit-specific, cold-dependent accumulation of anthocyanins in blood oranges. Plant Cell.

[bib11] Capella-Gutiérrez S., Silla-Martínez J.M., Gabaldón T. (2009). trimAl: a tool for automated alignment trimming in large-scale phylogenetic analyses. Bioinformatics.

[bib12] Catoni M., Jonesman T., Cerruti E., Paszkowski J. (2019). Mobilization of Pack-CACTA transposons in *Arabidopsis* suggests the mechanism of gene shuffling. Nucleic Acids Res..

[bib13] Chen C., Wu Y., Li J., Wang X., Zeng Z., Xu J., Liu Y., Feng J., Chen H., He Y. (2023). TBtools-II: A “one for all, all for one” bioinformatics platform for biological big-data mining. Mol. Plant.

[bib14] Chen D., Zhang T., Chen Y., Ma H., Qi J. (2022). Tree2GD: a phylogenomic method to detect large-scale gene duplication events. Bioinformatics.

[bib15] Chen S., Zhou Y., Chen Y., Gu J. (2018). fastp: an ultra-fast all-in-one FASTQ preprocessor. Bioinformatics.

[bib16] Chin C.S., Alexander D.H., Marks P., Klammer A.A., Drake J., Heiner C., Clum A., Copeland A., Huddleston J., Eichler E.E. (2013). Nonhybrid, finished microbial genome assemblies from long-read SMRT sequencing data. Nat. Methods.

[bib17] Christenhusz M.J.M., Reveal J.L., Farjon A., Gardner M.F., Mill R.R., Chase M.W. (2011). A new classification and linear sequence of extant gymnosperms. Phytotaxa.

[bib18] Cuerda-Gil D., Slotkin R.K. (2016). Non-canonical RNA-directed DNA methylation. Nat. Plants.

[bib19] Cui L., Wall P.K., Leebens-Mack J.H., Lindsay B.G., Soltis D.E., Doyle J.J., Soltis P.S., Carlson J.E., Arumuganathan K., Barakat A. (2006). Widespread genome duplications throughout the history of flowering plants. Genome Res..

[bib20] De La Torre A.R., Piot A., Liu B., Wilhite B., Weiss M., Porth I. (2020). Functional and morphological evolution in gymnosperms: A portrait of implicated gene families. Evol. Appl..

[bib21] Dobin A., Davis C.A., Schlesinger F., Drenkow J., Zaleski C., Jha S., Batut P., Chaisson M., Gingeras T.R. (2013). STAR: ultrafast universal RNA-seq aligner. Bioinformatics.

[bib22] Drongitis D., Aniello F., Fucci L., Donizetti A. (2019). Roles of transposable elements in the different layers of gene expression regulation. Int. J. Mol. Sci..

[bib23] Edgar R.C. (2004). MUSCLE: multiple sequence alignment with high accuracy and high throughput. Nucleic Acids Res..

[bib24] Ellinghaus D., Kurtz S., Willhoeft U. (2008). LTRharvest, an efficient and flexible software for *de novo* detection of LTR retrotransposons. BMC Bioinf..

[bib25] Emms D.M., Kelly S. (2019). OrthoFinder: phylogenetic orthology inference for comparative genomics. Genome Biol..

[bib26] Fang Y., Qin X., Liao Q., Du R., Luo X., Zhou Q., Li Z., Chen H., Jin W., Yuan Y. (2022). The genome of homosporous maidenhair fern sheds light on the euphyllophyte evolution and defences. Nat. Plants.

[bib27] Flynn J.M., Hubley R., Goubert C., Rosen J., Clark A.G., Feschotte C., Smit A.F. (2020). RepeatModeler2 for automated genomic discovery of transposable element families. Proc. Natl. Acad. Sci. USA.

[bib28] Fu L., Niu B., Zhu Z., Wu S., Li W. (2012). CD-HIT: accelerated for clustering the next-generation sequencing data. Bioinformatics.

[bib29] Galindo-González L., Mhiri C., Deyholos M.K., Grandbastien M.-A. (2017). LTR-retrotransposons in plants: Engines of evolution. Gene.

[bib30] Gozashti L., Harringmeyer O.S., Hoekstra H.E. (2025). How repeats rearrange chromosomes: the molecular basis of chromosomal inversions in deer mice. Cell Rep..

[bib31] Grabherr M.G., Haas B.J., Yassour M., Levin J.Z., Thompson D.A., Amit I., Adiconis X., Fan L., Raychowdhury R., Zeng Q. (2011). Full-length transcriptome assembly from RNA-Seq data without a reference genome. Nat. Biotechnol..

[bib32] Grant C.E., Bailey T.L., Noble W.S. (2011). FIMO: scanning for occurrences of a given motif. Bioinformatics.

[bib33] Haas B.J., Salzberg S.L., Zhu W., Pertea M., Allen J.E., Orvis J., White O., Buell C.R., Wortman J.R. (2008). Automated eukaryotic gene structure annotation using EVidenceModeler and the Program to Assemble Spliced Alignments. Genome Biol..

[bib34] Haas B.J., Delcher A.L., Mount S.M., Wortman J.R., Smith R.K., Hannick L.I., Maiti R., Ronning C.M., Rusch D.B., Town C.D. (2003). Improving the *Arabidopsis* genome annotation using maximal transcript alignment assemblies. Nucleic Acids Res..

[bib35] Han Z., Wang Z., Rittschof D., Huang Z., Chen L., Hao H., Yao S., Su P., Huang M., Zhang Y.Y. (2024). New genes helped acorn barnacles adapt to a sessile lifestyle. Nat. Genet..

[bib36] Hawkins J.S., Proulx S.R., Rapp R.A., Wendel J.F. (2009). Rapid DNA loss as a counterbalance to genome expansion through retrotransposon proliferation in plants. Proc. Natl. Acad. Sci. USA.

[bib37] Hollister J.D., Smith L.M., Guo Y.-L., Ott F., Weigel D., Gaut B.S. (2011). Transposable elements and small RNAs contribute to gene expression divergence between *Arabidopsis thaliana* and *Arabidopsis lyrata*. Proc. Natl. Acad. Sci. USA.

[bib38] Holt C., Yandell M. (2011). MAKER2: an annotation pipeline and genome-database management tool for second-generation genome projects. BMC Bioinf..

[bib39] Hu J., Fan J., Sun Z., Liu S. (2020). NextPolish: a fast and efficient genome polishing tool for long-read assembly. Bioinformatics.

[bib40] Hu J., Wang Z., Sun Z., Hu B., Ayoola A.O., Liang F., Li J., Sandoval J.R., Cooper D.N., Ye K. (2024). NextDenovo: an efficient error correction and accurate assembly tool for noisy long reads. Genome Biol..

[bib41] Hu X.G., Liu H., Jin Y., Sun Y.Q., Li Y., Zhao W., El-Kassaby Y.A., Wang X.R., Mao J.F. (2016). *De novo* transcriptome assembly and characterization for the widespread and stress-tolerant conifer *Platycladus orientalis*. PLoS One.

[bib42] Huang N., Li H. (2023). compleasm: a faster and more accurate reimplementation of BUSCO. Bioinformatics.

[bib43] Huang Y., He J., Xu Y., Zheng W., Wang S., Chen P., Zeng B., Yang S., Jiang X., Liu Z. (2023). Pangenome analysis provides insight into the evolution of the orange subfamily and a key gene for citric acid accumulation in citrus fruits. Nat. Genet..

[bib44] Huerta-Cepas J., Forslund K., Coelho L.P., Szklarczyk D., Jensen L.J., von Mering C., Bork P. (2017). Fast genome-wide functional annotation through orthology assignment by eggNOG-Mapper. Mol. Biol. Evol..

[bib45] Innan H., Kondrashov F. (2010). The evolution of gene duplications: classifying and distinguishing between models. Nat. Rev. Genet..

[bib46] Jang M.-J., Cho H.J., Park Y.-S., Lee H.-Y., Bae E.-K., Jung S., Jin H., Woo J., Park E., Kim S.-J. (2024). Haplotype-resolved genome assembly and resequencing analysis provide insights into genome evolution and allelic imbalance in *Pinus densiflora*. Nat. Genet..

[bib47] Jayakodi M., Golicz A.A., Kreplak J., Fechete L.I., Angra D., Bednář P., Bornhofen E., Zhang H., Boussageon R., Kaur S. (2023). The giant diploid faba genome unlocks variation in a global protein crop. Nature.

[bib48] Jia K.H., Zhao W., Maier P.A., Hu X.G., Jin Y., Zhou S.S., Jiao S.Q., El-Kassaby Y.A., Wang T., Wang X.R. (2020). Landscape genomics predicts climate change-related genetic offset for the widespread *Platycladus orientalis* (Cupressaceae). Evol. Appl..

[bib49] Jiang N., Bao Z., Zhang X., Eddy S.R., Wessler S.R. (2004). Pack-MULE transposable elements mediate gene evolution in plants. Nature.

[bib50] Jin Y., Zhao W., Nie S., Liu S.S., El-Kassaby Y.A., Wang X.R., Mao J.F. (2019). Genome-wide variant identification and high-density genetic map construction using RADseq for *Platycladus orientalis* (Cupressaceae). G3 (Bethesda)..

[bib51] Kapli P., Yang Z., Telford M.J. (2020). Phylogenetic tree building in the genomic age. Nat. Rev. Genet..

[bib52] Katoh K., Standley D.M. (2013). MAFFT multiple sequence alignment software version 7: improvements in performance and usability. Mol. Biol. Evol..

[bib53] Kerseviciute I., Gordevicius J. (2023). aPEAR: an R package for autonomous visualization of pathway enrichment networks. Bioinformatics.

[bib54] Kiełbasa S.M., Wan R., Sato K., Horton P., Frith M.C. (2011). Adaptive seeds tame genomic sequence comparison. Genome Res..

[bib55] Kim D., Langmead B., Salzberg S.L. (2015). HISAT: a fast spliced aligner with low memory requirements. Nat. Methods.

[bib56] Kimura M. (1980). A simple method for estimating evolutionary rates of base substitutions through comparative studies of nucleotide sequences. J. Mol. Evol..

[bib57] Koch M.A., Haubold B., Mitchell-Olds T. (2000). Comparative evolutionary analysis of chalcone synthase and alcohol dehydrogenase loci in *Arabidopsis, Arabis*, and related genera (Brassicaceae). Mol. Biol. Evol..

[bib58] Koren S., Walenz B.P., Berlin K., Miller J.R., Bergman N.H., Phillippy A.M. (2017). Canu: scalable and accurate long-read assembly via adaptive k-mer weighting and repeat separation. Genome Res..

[bib59] Krasileva K.V. (2019). The role of transposable elements and DNA damage repair mechanisms in gene duplications and gene fusions in plant genomes. Curr. Opin. Plant Biol..

[bib60] Krueger F., Andrews S.R. (2011). Bismark: a flexible aligner and methylation caller for Bisulfite-Seq applications. Bioinformatics.

[bib61] Li H. (2018). Minimap2: pairwise alignment for nucleotide sequences. Bioinformatics.

[bib62] Li H., Handsaker B., Wysoker A., Fennell T., Ruan J., Homer N., Marth G., Abecasis G., Durbin R., 1000 Genome Project Data Processing Subgroup (2009). The sequence alignment/map format and SAMtools. Bioinformatics.

[bib63] Li Z., Gilbert C., Peng H., Pollet N. (2024). Discovery of numerous novel *Helitron*-like elements in eukaryote genomes using HELIANO. Nucleic Acids Res..

[bib64] Liao Y., Smyth G.K., Shi W. (2019). The R package Rsubread is easier, faster, cheaper and better for alignment and quantification of RNA sequencing reads. Nucleic Acids Res..

[bib65] Lim J.Q., Tennakoon C., Li G., Wong E., Ruan Y., Wei C.L., Sung W.K. (2012). BatMeth: improved mapper for bisulfite sequencing reads on DNA methylation. Genome Biol..

[bib66] Lisch D. (2013). How important are transposons for plant evolution?. Nat. Rev. Genet..

[bib67] Liu H., Wu S., Li A., Ruan J. (2021). SMARTdenovo: a *de novo* assembler using long noisy reads. GigaByte.

[bib68] Liu H., Wang X., Wang G., Cui P., Wu S., Ai C., Hu N., Li A., He B., Shao X. (2021). The nearly complete genome of *Ginkgo biloba* illuminates gymnosperm evolution. Nat. Plants.

[bib69] Liu H., Yan X.M., Wang X.R., Zhang D.X., Zhou Q., Shi T.L., Jia K.H., Tian X.C., Zhou S.S., Zhang R.G. (2021). Centromere-specific retrotransposons and very-long-chain fatty acid biosynthesis in the genome of yellowhorn (*Xanthoceras sorbifolium*, Sapindaceae), an oil-producing tree with significant drought resistance. Front. Plant Sci..

[bib70] Liu Y., Wang S., Li L., Yang T., Dong S., Wei T., Wu S., Liu Y., Gong Y., Feng X. (2022). The *Cycas* genome and the early evolution of seed plants. Nat. Plants.

[bib71] Lockton S., Gaut B.S. (2009). The contribution of transposable elements to expressed coding sequence in *Arabidopsis thaliana*. J. Mol. Evol..

[bib72] Lou H., Song L., Li X., Zi H., Chen W., Gao Y., Zheng S., Fei Z., Sun X., Wu J. (2023). The *Torreya grandis* genome illuminates the origin and evolution of gymnosperm-specific sciadonic acid biosynthesis. Nat. Commun..

[bib73] Lowe T.M., Eddy S.R. (1997). tRNAscan-SE: a program for improved detection of transfer RNA genes in genomic sequence. Nucleic Acids Res..

[bib74] Lyu H., He Z., Wu C.I., Shi S. (2018). Convergent adaptive evolution in marginal environments: unloading transposable elements as a common strategy among mangrove genomes. New Phytol..

[bib75] Ma H., Wang M., Zhang Y.E., Tan S. (2023). The power of “controllers”: Transposon-mediated duplicated genes evolve towards neofunctionalization. J. Genet. Genomics.

[bib76] Ma J., Devos K.M., Bennetzen J.L. (2004). Analyses of LTR-Retrotransposon Structures Reveal Recent and Rapid Genomic DNA Loss in Rice. Genome Res..

[bib77] Ma J., Sun P., Wang D., Wang Z., Yang J., Li Y., Mu W., Xu R., Wu Y., Dong C. (2021). The *Chloranthus sessilifolius* genome provides insight into early diversification of angiosperms. Nat. Commun..

[bib78] Marçais G., Kingsford C. (2011). A fast, lock-free approach for efficient parallel counting of occurrences of *k*-mers. Bioinformatics.

[bib79] Marçais G., Delcher A.L., Phillippy A.M., Coston R., Salzberg S.L., Zimin A. (2018). MUMmer4: A fast and versatile genome alignment system. PLoS Comput. Biol..

[bib80] Matzke M.A., Mosher R.A. (2014). RNA-directed DNA methylation: an epigenetic pathway of increasing complexity. Nat. Rev. Genet..

[bib81] Mendes F.K., Vanderpool D., Fulton B., Hahn M.W. (2021). CAFE 5 models variation in evolutionary rates among gene families. Bioinformatics.

[bib82] Muyle A., Seymour D., Darzentas N., Primetis E., Gaut B.S., Bousios A. (2021). Gene capture by transposable elements leads to epigenetic conflict in maize. Mol. Plant.

[bib83] Neumann P., Novák P., Hoštáková N., Macas J. (2019). Systematic survey of plant LTR-retrotransposons elucidates phylogenetic relationships of their polyprotein domains and provides a reference for element classification. Mob. DNA.

[bib84] Nevers Y., Warwick Vesztrocy A., Rossier V., Train C.-M., Altenhoff A., Dessimoz C., Glover N.M. (2025). Quality assessment of gene repertoire annotations with OMArk. Nat. Biotechnol..

[bib85] Nguyen L.T., Schmidt H.A., von Haeseler A., Minh B.Q. (2015). IQ-TREE: a fast and effective stochastic algorithm for estimating maximum-likelihood phylogenies. Mol. Biol. Evol..

[bib86] Nie S., Zhao S.-W., Shi T.-L., Zhao W., Zhang R.-G., Tian X.-C., Guo J.-F., Yan X.-M., Bao Y.-T., Li Z.-C. (2023). Gapless genome assembly of azalea and multi-omics investigation into divergence between two species with distinct flower color. Hortic. Res..

[bib87] Niederhuth C.E., Bewick A.J., Ji L., Alabady M.S., Kim K.D., Li Q., Rohr N.A., Rambani A., Burke J.M., Udall J.A. (2016). Widespread natural variation of DNA methylation within angiosperms. Genome Biol..

[bib88] Niu S., Li J., Bo W., Yang W., Zuccolo A., Giacomello S., Chen X., Han F., Yang J., Song Y. (2022). The Chinese pine genome and methylome unveil key features of conifer evolution. Cell.

[bib89] Nystedt B., Street N.R., Wetterbom A., Zuccolo A., Lin Y.C., Scofield D.G., Vezzi F., Delhomme N., Giacomello S., Alexeyenko A. (2013). The Norway spruce genome sequence and conifer genome evolution. Nature.

[bib90] Ohri D. (2021). Variation and evolution of genome size in gymnosperms. Silvae Genet..

[bib91] Ou S., Jiang N. (2018). LTR_retriever: a highly accurate and sensitive program for identification of long terminal repeat retrotransposons. Plant Physiol..

[bib92] Ou S., Su W., Liao Y., Chougule K., Agda J.R.A., Hellinga A.J., Lugo C.S.B., Elliott T.A., Ware D., Peterson T. (2019). Benchmarking transposable element annotation methods for creation of a streamlined, comprehensive pipeline. Genome Biol..

[bib93] Panchy N., Lehti-Shiu M., Shiu S.H. (2016). Evolution of gene duplication in plants. Plant Physiol..

[bib94] Paradis E., Schliep K. (2019). ape 5.0: an environment for modern phylogenetics and evolutionary analyses in R. Bioinformatics.

[bib95] Pertea M., Pertea G.M., Antonescu C.M., Chang T.C., Mendell J.T., Salzberg S.L. (2015). StringTie enables improved reconstruction of a transcriptome from RNA-seq reads. Nat. Biotechnol..

[bib96] Pryszcz L.P., Gabaldón T. (2016). Redundans: an assembly pipeline for highly heterozygous genomes. Nucleic Acids Res..

[bib97] Qiao X., Zhang S., Paterson A.H. (2022). Pervasive genome duplications across the plant tree of life and their links to major evolutionary innovations and transitions. Comput. Struct. Biotechnol. J..

[bib98] Qiao X., Li Q., Yin H., Qi K., Li L., Wang R., Zhang S., Paterson A.H. (2019). Gene duplication and evolution in recurring polyploidization–diploidization cycles in plants. Genome Biol..

[bib99] Ramírez F., Dündar F., Diehl S., Grüning B.A., Manke T. (2014). deepTools: a flexible platform for exploring deep-sequencing data. Nucleic Acids Res..

[bib100] Ranwez V., Douzery E.J.P., Cambon C., Chantret N., Delsuc F. (2018). MACSE v2: toolkit for the alignment of coding sequences accounting for frameshifts and stop codons. Mol. Biol. Evol..

[bib101] Rebollo R., Romanish M.T., Mager D.L. (2012). Transposable elements: an abundant and natural source of regulatory sequences for host genes. Annu. Rev. Genet..

[bib102] Rhie A., Walenz B.P., Koren S., Phillippy A.M. (2020). Merqury: reference-free quality, completeness, and phasing assessment for genome assemblies. Genome Biol..

[bib103] Shi J., Liang C. (2019). Generic repeat finder: a high-sensitivity tool for genome-wide *de novo* repeat detection. Plant Physiol..

[bib104] Simão F.A., Waterhouse R.M., Ioannidis P., Kriventseva E.V., Zdobnov E.M. (2015). BUSCO: assessing genome assembly and annotation completeness with single-copy orthologs. Bioinformatics.

[bib105] Stanke M., Diekhans M., Baertsch R., Haussler D. (2008). Using native and syntenically mapped cDNA alignments to improve *de novo* gene finding. Bioinformatics.

[bib106] Steinbiss S., Willhoeft U., Gremme G., Kurtz S. (2009). Fine-grained annotation and classification of *de novo* predicted LTR retrotransposons. Nucleic Acids Res..

[bib107] Steinthorsdottir M., Coxall H.K., de Boer A.M., Huber M., Barbolini N., Bradshaw C.D., Burls N.J., Feakins S.J., Gasson E., Henderiks J. (2021). The Miocene: The future of the past. Paleoceanogr. Paleoclimatol..

[bib108] Su W., Gu X., Peterson T. (2019). TIR-Learner, a new ensemble method for TIR transposable element annotation, provides evidence for abundant new transposable elements in the maize genome. Mol. Plant.

[bib109] Sun P., Jiao B., Yang Y., Shan L., Li T., Li X., Xi Z., Wang X., Liu J. (2022). WGDI: A user-friendly toolkit for evolutionary analyses of whole-genome duplications and ancestral karyotypes. Mol. Plant.

[bib110] Tan S., Cardoso-Moreira M., Shi W., Zhang D., Huang J., Mao Y., Jia H., Zhang Y., Chen C., Shao Y. (2016). LTR-mediated retroposition as a mechanism of RNA-based duplication in metazoans. Genome Res..

[bib111] Tang H., Bowers J.E., Wang X., Ming R., Alam M., Paterson A.H. (2008). Synteny and collinearity in plant genomes. Science.

[bib112] Teissandier A., Servant N., Barillot E., Bourc'his D. (2019). Tools and best practices for retrotransposon analysis using high-throughput sequencing data. Mob. DNA.

[bib113] Tenaillon M.I., Hollister J.D., Gaut B.S. (2010). A triptych of the evolution of plant transposable elements. Trends Plant Sci..

[bib114] Thorvaldsdóttir H., Robinson J.T., Mesirov J.P. (2013). Integrative Genomics Viewer (IGV): high-performance genomics data visualization and exploration. Briefings Bioinf..

[bib115] Tian F., Yang D.-C., Meng Y.-Q., Jin J., Gao G. (2020). PlantRegMap: charting functional regulatory maps in plants. Nucleic Acids Res..

[bib116] Tian J., Wang C., Chen F., Qin W., Yang H., Zhao S., Xia J., Du X., Zhu Y., Wu L. (2024). Maize smart-canopy architecture enhances yield at high densities. Nature.

[bib117] Van de Peer Y., Mizrachi E., Marchal K. (2017). The evolutionary significance of polyploidy. Nat. Rev. Genet..

[bib118] Vermeij G.J. (2008). Escalation and its role in Jurassic biotic history. Palaeogeogr. Palaeoclimatol. Palaeoecol..

[bib119] Wan T., Gong Y., Liu Z., Zhou Y., Dai C., Wang Q. (2022). Evolution of complex genome architecture in gymnosperms. GigaScience.

[bib120] Wan T., Liu Z., Leitch I.J., Xin H., Maggs-Kölling G., Gong Y., Li Z., Marais E., Liao Y., Dai C. (2021). The *Welwitschia* genome reveals a unique biology underpinning extreme longevity in deserts. Nat. Commun..

[bib121] Wang D., Zhang Y., Zhang Z., Zhu J., Yu J. (2010). KaKs_Calculator 2.0: a toolkit incorporating gamma-series methods and sliding window strategies. Genom. Proteom. Bioinform..

[bib122] Wang K., Wang J., Zhu C., Yang L., Ren Y., Ruan J., Fan G., Hu J., Xu W., Bi X. (2021). African lungfish genome sheds light on the vertebrate water-to-land transition. Cell.

[bib123] Wang T., Duan S., Xu C., Wang Y., Zhang X., Xu X., Chen L., Han Z., Wu T. (2023). Pan-genome analysis of 13 *Malus* accessions reveals structural and sequence variations associated with fruit traits. Nat. Commun..

[bib124] Wang X., Yan X., Hu Y., Qin L., Wang D., Jia J., Jiao Y. (2022). A recent burst of gene duplications in Triticeae. Plant Commun..

[bib125] Wang X.Q., Ran J.H. (2014). Evolution and biogeography of gymnosperms. Mol. Phylogenet. Evol..

[bib126] Wang Y., Wang X., Paterson A.H. (2012). Genome and gene duplications and gene expression divergence: a view from plants. Ann. N. Y. Acad. Sci..

[bib127] Wang Y., Tang H., Debarry J.D., Tan X., Li J., Wang X., Lee T.H., Jin H., Marler B., Guo H. (2012). MCScanX: a toolkit for detection and evolutionary analysis of gene synteny and collinearity. Nucleic Acids Res..

[bib128] Wegrzyn J.L., Liechty J.D., Stevens K.A., Wu L.S., Loopstra C.A., Vasquez-Gross H.A., Dougherty W.M., Lin B.Y., Zieve J.J., Martínez-García P.J. (2014). Unique features of the loblolly pine (*Pinus taeda* L.) megagenome revealed through sequence annotation. Genetics.

[bib129] Wicker T., Buchmann J.P., Keller B. (2010). Patching gaps in plant genomes results in gene movement and erosion of colinearity. Genome Res..

[bib130] Wu T., Hu E., Xu S., Chen M., Guo P., Dai Z., Feng T., Zhou L., Tang W., Zhan L. (2021). clusterProfiler 4.0: A universal enrichment tool for interpreting omics data. Innovation.

[bib131] Xia B., Zhang W., Zhao G., Zhang X., Bai J., Brosh R., Wudzinska A., Huang E., Ashe H., Ellis G. (2024). On the genetic basis of tail-loss evolution in humans and apes. Nature.

[bib132] Xiao P.X., Li Y., Lu J., Zuo H., Pingcuo G., Ying H., Zhao F., Xu Q., Zeng X., Jiao W.B. (2023). High-quality assembly and methylome of a Tibetan wild tree peony genome (*Paeonia ludlowii*) reveal the evolution of giant genome architecture. Hortic. Res..

[bib133] Xie J., Chen Y., Cai G., Cai R., Hu Z., Wang H. (2023). Tree Visualization By One Table (tvBOT): a web application for visualizing, modifying and annotating phylogenetic trees. Nucleic Acids Res..

[bib134] Xiong W., He L., Lai J., Dooner H.K., Du C. (2014). HelitronScanner uncovers a large overlooked cache of *Helitron* transposons in many plant genomes. Proc. Natl. Acad. Sci. USA.

[bib135] Xu C., Xu Y., Wang Z., Zhang X., Wu Y., Lu X., Sun H., Wang L., Zhang Q., Zhang Q. (2023). Spontaneous movement of a retrotransposon generated genic dominant male sterility providing a useful tool for rice breeding. Natl. Sci. Rev..

[bib136] Xu Z., Wang H. (2007). LTR_FINDER: an efficient tool for the prediction of full-length LTR retrotransposons. Nucleic Acids Res..

[bib137] Yan H., Bombarely A., Li S. (2020). DeepTE: a computational method for *de novo* classification of transposons with convolutional neural network. Bioinformatics.

[bib138] Yang S., Arguello J.R., Li X., Ding Y., Zhou Q., Chen Y., Zhang Y., Zhao R., Brunet F., Peng L. (2008). Repetitive element-mediated recombination as a mechanism for new gene origination in *Drosophila*. PLoS Genet..

[bib139] Yang Z. (2007). PAML 4: phylogenetic analysis by maximum likelihood. Mol. Biol. Evol..

[bib140] Ye M., Goudot C., Hoyler T., Lemoine B., Amigorena S., Zueva E. (2020). Specific subfamilies of transposable elements contribute to different domains of T lymphocyte enhancers. Proc. Natl. Acad. Sci. USA.

[bib141] Yuan J., Jiang S., Jian J., Liu M., Yue Z., Xu J., Li J., Xu C., Lin L., Jing Y. (2022). Genomic basis of the giga-chromosomes and giga-genome of tree peony *Paeonia ostii*. Nat. Commun..

[bib142] Zhang A., Zhang W. (2022). Characterization of transposon-derived accessible chromatin regions in rice (*Oryza sativa*). Int. J. Mol. Sci..

[bib143] Zhang R.G., Li G.Y., Wang X.L., Dainat J., Wang Z.X., Ou S., Ma Y. (2022). TEsorter: an accurate and fast method to classify LTR-retrotransposons in plant genomes. Hortic. Res..

[bib144] Zhang Y., Li Z., Liu J., Zhang Y., Ye L., Peng Y., Wang H., Diao H., Ma Y., Wang M. (2022). Transposable elements orchestrate subgenome-convergent and-divergent transcription in common wheat. Nat. Commun..

[bib145] Zhang R.G., Liu H., Shang H.Y., Shu H., Liu D.T., Yang H., Jia K.H., Wang X.Q., Sun W.B., Zhao W. (2025). Convergent patterns of karyotype evolution underlying karyotype uniformity in conifers. Adv. Sci..

[bib146] Zhang Y., Liu T., Meyer C.A., Eeckhoute J., Johnson D.S., Bernstein B.E., Nusbaum C., Myers R.M., Brown M., Li W., Liu X.S. (2008). Model-based analysis of ChIP-Seq (MACS). Genome Biol..

[bib147] Zhang Y., Li Z., Zhang Y., Lin K., Peng Y., Ye L., Zhuang Y., Wang M., Xie Y., Guo J. (2021). Evolutionary rewiring of the wheat transcriptional regulatory network by lineage-specific transposable elements. Genome Res..

[bib148] Zhang Z., Carriero N., Zheng D., Karro J., Harrison P.M., Gerstein M. (2006). PseudoPipe: an automated pseudogene identification pipeline. Bioinformatics.

[bib149] Zhang Z., Xiao J., Wu J., Zhang H., Liu G., Wang X., Dai L. (2012). ParaAT: a parallel tool for constructing multiple protein-coding DNA alignments. Biochem. Biophys. Res. Commun..

[bib150] Zhao D., Ferguson A.A., Jiang N. (2016). What makes up plant genomes: The vanishing line between transposable elements and genes. Biochim. Biophys. Acta.

[bib151] Zhao D., Hamilton J.P., Vaillancourt B., Zhang W., Eizenga G.C., Cui Y., Jiang J., Buell C.R., Jiang N. (2018). The unique epigenetic features of Pack-MULEs and their impact on chromosomal base composition and expression spectrum. Nucleic Acids Res..

[bib152] Zhou S.S., Yan X.M., Zhang K.F., Liu H., Xu J., Nie S., Jia K.H., Jiao S.Q., Zhao W., Zhao Y.J. (2021). A comprehensive annotation dataset of intact LTR retrotransposons of 300 plant genomes. Sci. Data.

