# Supplementary material for: A chromosome-level genome assembly of Platycladus orientalis and comparative genomics reveal pivotal roles of transposable elements in gene duplication and pseudogenization across gymnosperm giga-genomes
Source: Plant Commun. 2026 Mar 10;7(8):101814. doi: 10.1016/j.xplc.2026.101814 (PMC13477047; doi:10.1016/j.xplc.2026.101814)
Supplement: Supplementary file 1 [file mmc1.pdf]

Plant Communications, Volume *7*

Supplemental information

A chromosome-level genome assembly of *Platycladus orientalis* and comparative genomics reveal pivotal roles of transposable elements in gene duplication and pseudogenization across gymnosperm giga-genomes

Yu-Tao Bao, Ren-Gang Zhang, Hui Liu, Zhi-Chao Li, Si-Qian Jiao, Kai-Hua Jia, Shan-Shan Zhou, Shuai Nie, Xue-Mei Yan, Tian-Le Shi, Xue-Chan Tian, Shi-Wei Zhao, Lei Kong, Zhao-Yang Chen, Hai-Yao Ma, Xiao-Lei Yang, Charles Chen, Yousry Aly El-Kassaby, Ilga Porth, Xiao-Ru Wang, Jian-Feng Mao, and Wei Zhao

A chromosome-level genome assembly of *Platycladus orientalis* and comparative genomics reveal pivotal roles of transposable elements in gene duplication and pseudogenization across gymnosperm giga-genomes

Supplemental Figures

Supplemental Figure 1. Schematic workflow of genome assembly and gene annotation for *P. orientalis*.
Supplemental Figure 2. *K*-mer frequency distribution estimated from Illumina sequences after filtering and correction at *k*-mer size of 17.
Supplemental Figure 3. BUSCO (Benchmarking Universal Single-Copy Orthologs) analysis of *P. orientalis* and the available representative gymnosperm genomes.
Supplemental Figure 4. Evaluation of the *P. orientalis* genome assembly.
Supplemental Figure 5. Schematic workflow for the genome-wide transposable element (TE) annotation in gymnosperms.
Supplemental Figure 6. Network and clustering of the significantly expanded, contracted and species-specific genes GO terms obtained through enrichment analysis.
Supplemental Figure 7. The distribution of *K*s values of the syntenic gene pairs within and among species of *P. orientalis* and eleven gymnosperm species.
Supplemental Figure 8. Detection of focal nodes labeling whole-genome duplications using phylogenomic Tree2GD approach.
Supplemental Figure 9. Synteny depths analysis between *P. orientalis* and ten gymnosperm species.
Supplemental Figure 10. Intergenomic dot plot of synteny between *P. orientalis* and ten gymnosperm species.
Supplemental Figure 11. Collinearity between the chromosomes of *P. orientalis* and ten gymnosperm species.
Supplemental Figure 12. Phylogenetic trees of *Ty3*/*Gypsy* and *Ty1*/*Copia* LTR-RT reverse transcriptase domains.
Supplemental Figure 13. Insertion time of (A) all TE types, (B) intact LTR-RT as detected in 12 gymnosperms, (C) all TE types and (D) intact LTR-RT in 11 angiosperms and pteridophytes.
Supplemental Figure 14. Correlations between genome size and genomic features.
Supplemental Figure 15. Evolutionary dynamics of intact LTR-RT families in (A) 12 gymnosperms and (B) 11 angiosperms and pteridophytes.
Supplemental Figure 16. UpSet plot of TE insertions relative to gene positions in *P. orientalis*.
Supplemental Figure 17. Genome-wide DNA methylation level of *P. orientalis* and 6 other plant genomes with high TE content (>60%) and genome size (>10G).
Supplemental Figure 18. The IGV presentation of expression, chromatin accessibility, and methylation levels in one ultra-long coding gene, exceeding 100 kb and harboring TE insertions.
Supplemental Figure 19. The tissue-specific expression of TE in *P. orientalis*.
Supplemental Figure 20. The association between TEs and accessible chromatin regions

(ACRs) in *P. orientalis*.

Supplemental Figure 21. Schematic illustration of evolutionary variations in the coding gene space.

Supplemental Figure 22. The number of genes (excluding TE-associated genes)derived from different duplication modes in 12 gymnosperms.

Supplemental Figure 23. *K*s distributions of duplicate gene pairs derived from 5 duplication modes in 12 gymnosperm species.

Supplemental Figure 24. Number of TE superfamilies mediating TE-recombination gene duplication.

Supplemental Figure 25. Proportion of different TE superfamilies mediating TE-recombination gene duplication.

Supplemental Figure 26. Comparison of the *K*a/*K*s values between TE-mediated gene pairs and those of other gene pairs across 12 different species.

Supplemental Figure 27. Functional enrichment of genes from TE-mediated four classes of small-scale gene duplication and two classes of pseudogenization in *P. orientalis* (adjusted *p*-value < 0.01).

Supplemental Figure 28. Contributions of TE-mediated gene duplication and pseudogenization events to significantly expansion, contraction, and species-specific dynamics of gene families in *P. orientalis*.

Supplemental Figure 29. Schematic representation of the identification of TE-mediated generation of duplicated pseudogenes.

Supplemental Figure 30. GO enrichment analysis of source genes of TE-mediated pseudogenization in 12 gymnosperm species.

Supplemental Figure 31. Network and clustering of GO terms derived from source genes of transcribed pseudogenes under positive selection, identified through enrichment analysis in *P. orientalis*.

Supplemental Figure 32. Chi-square test analyses of the proportion of single- exon and multi-exon genes within donor and free genes across 23 species.

Supplemental Figure 33. Chi-square test analyses of the proportion of captive exon types to non-captive exon types within multi-exon donor genes across 23 species.

Supplemental Figure 34. Comparative GO enrichment analysis of genes with captured fragments across 23 species (adjusted *p*-value < 0.05).

Supplemental Figure 35. Distribution of mCG, mCHG, and mCHH methylation levels in the exonic regions of the donor and free genes.

Supplemental Figure 36. Comparison of expression levels of four gene types in seven tissues.

Supplemental Figure 37. Distribution of mCG, mCHG, and mCHH methylation levels in three types of TEs.

Supplemental Figure 38. Hierarchical classification of 12 TE-mediated gene duplication mechanisms.

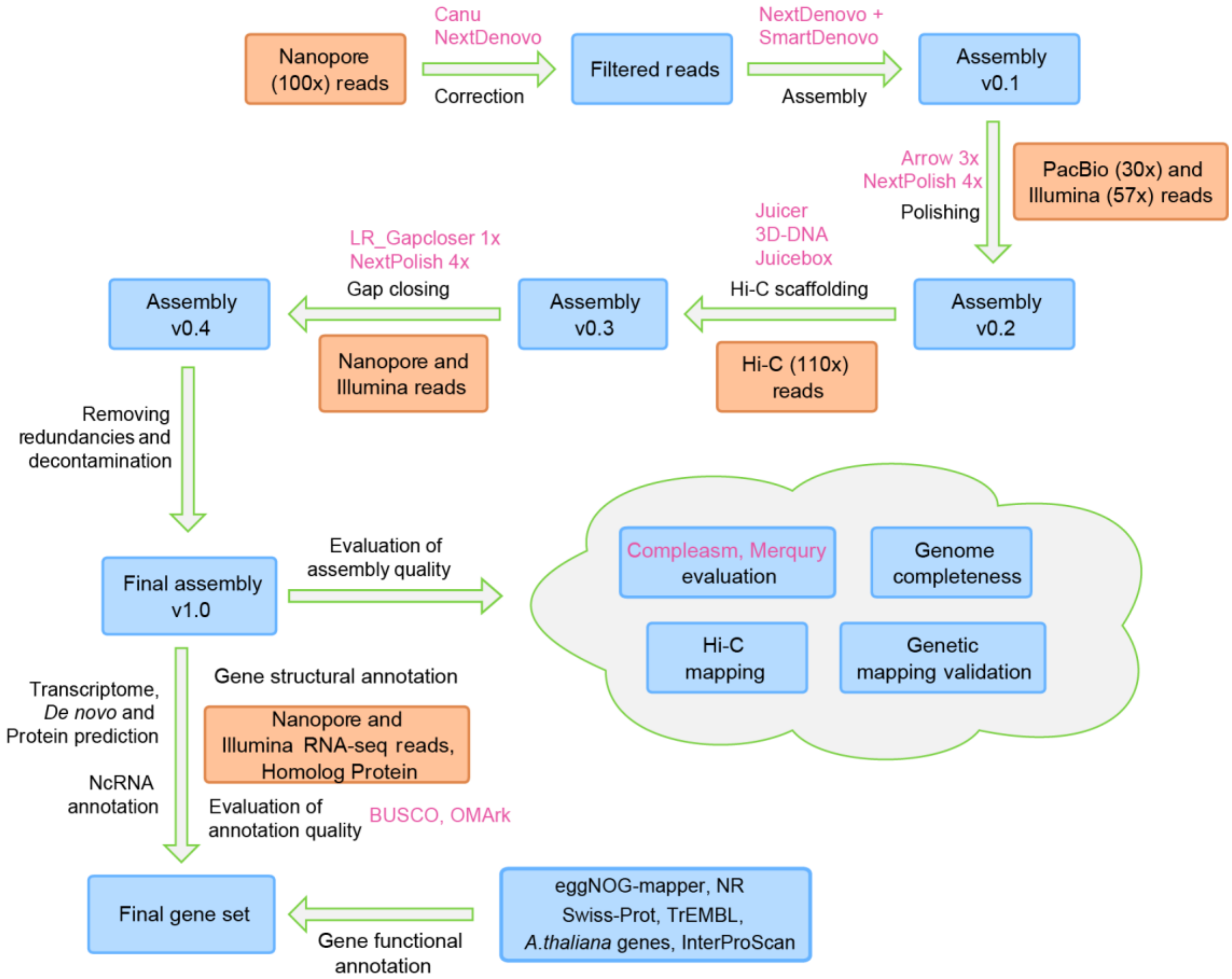

Supplemental Figure 1. Schematic workflow of genome assembly and gene annotation for *P. orientalis*.

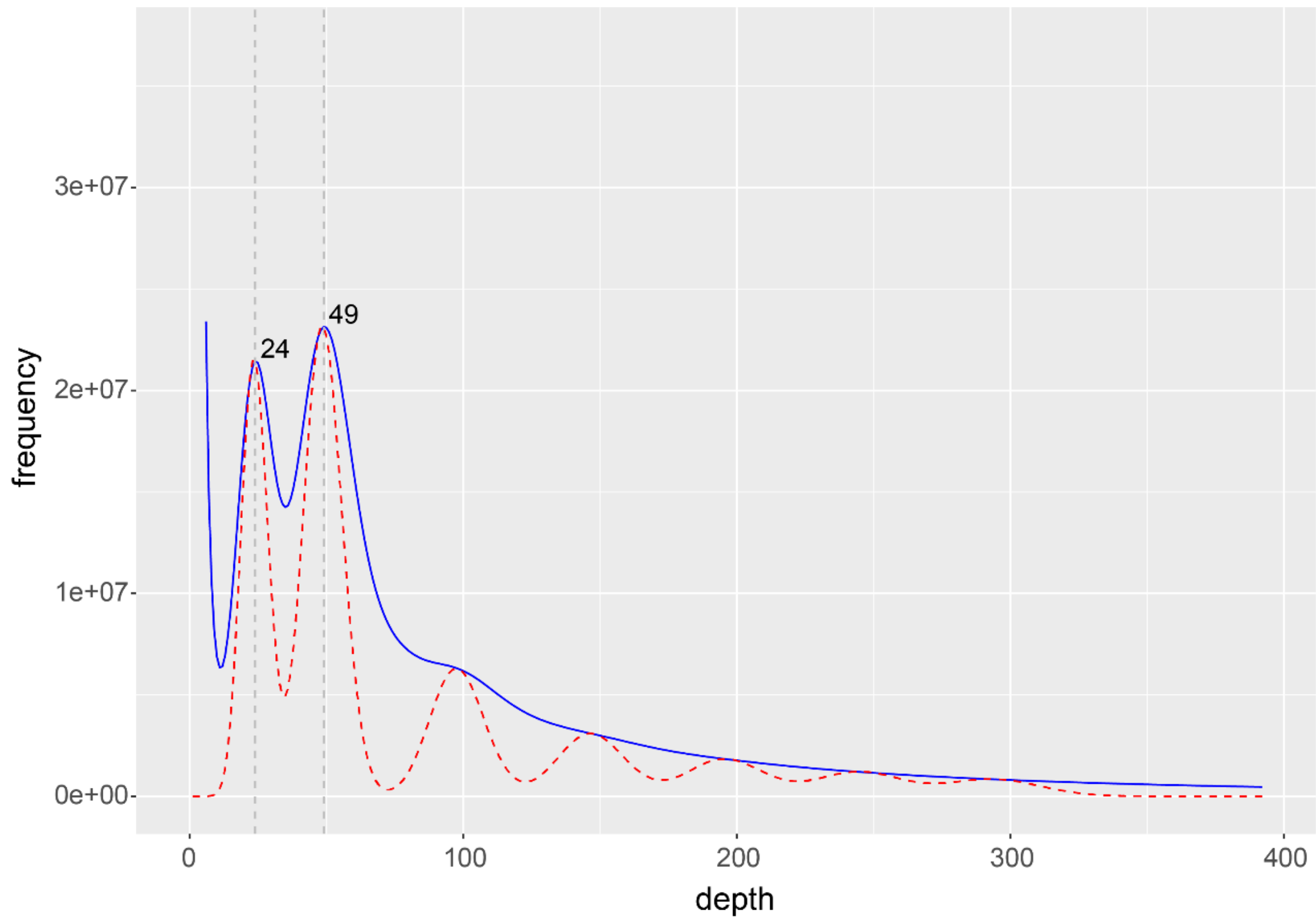

Supplemental Figure 2. *K*-mer frequency distribution estimated from Illumina sequences after filtering and correction at *k*-mer size of 17. The blue solid line represents the observed *k*-mer frequency distribution, while the red dashed line indicates the fitted model of the *k*-mer frequency distribution.

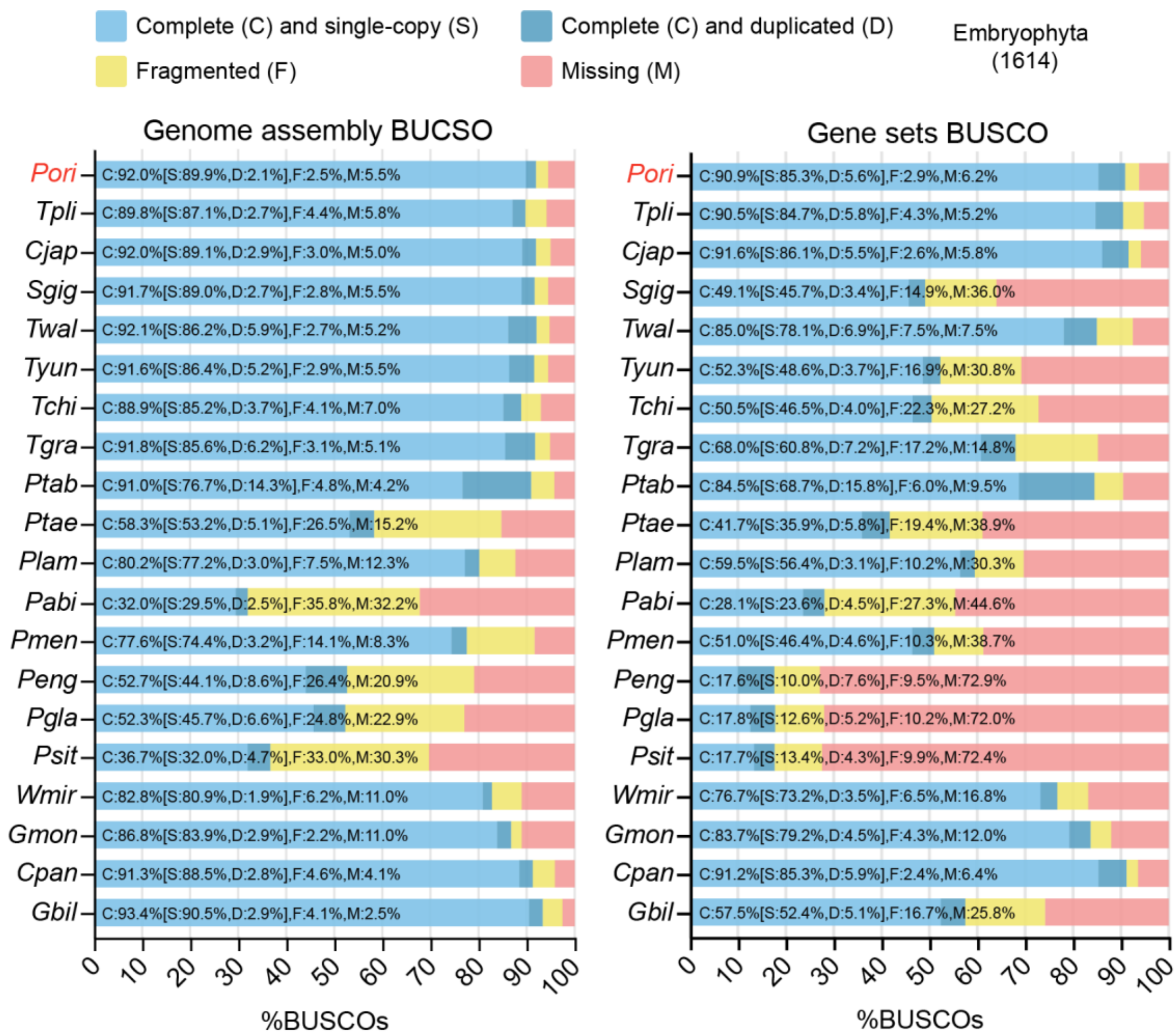

Supplemental Figure 3. BUSCO (Benchmarking Universal Single-Copy Orthologs) analysis of *P. orientalis* and the available representative gymnosperm genomes. *Pori*: *Platycladus orientalis*; *Tpli*: *Thuja plicata*; *Cjap*: *Cryptomeria japonica*; *Sgig*: *Sequoiadendron giganteum*; *Twal*: *Taxus wallichiana*; *Tyun*: *Taxus yunnanensis*; *Tchi*: *Taxus chinensis* var. *mairei*; *Tgra*: *Torreya grandis*; *Ptab*: *Pinus tabuliformis*; *Ptae*: *Pinus taeda*; *Plam*: *Pinus lambertiana*; *Pabi*: *Picea abies*; *Pmen*: *Pseudotsuga menziesii*; *Peng*: *Picea engelmannii*; *Pgla*: *Picea glauca*; *Psit*: *Picea sitchensis*; *Wmir*: *Welwitschia mirabilis*; *Gmon*: *Gnetum montanum*; *Cpan*: *Cycas panzhihuaensis*; *Gbil*: *Ginkgo biloba*.

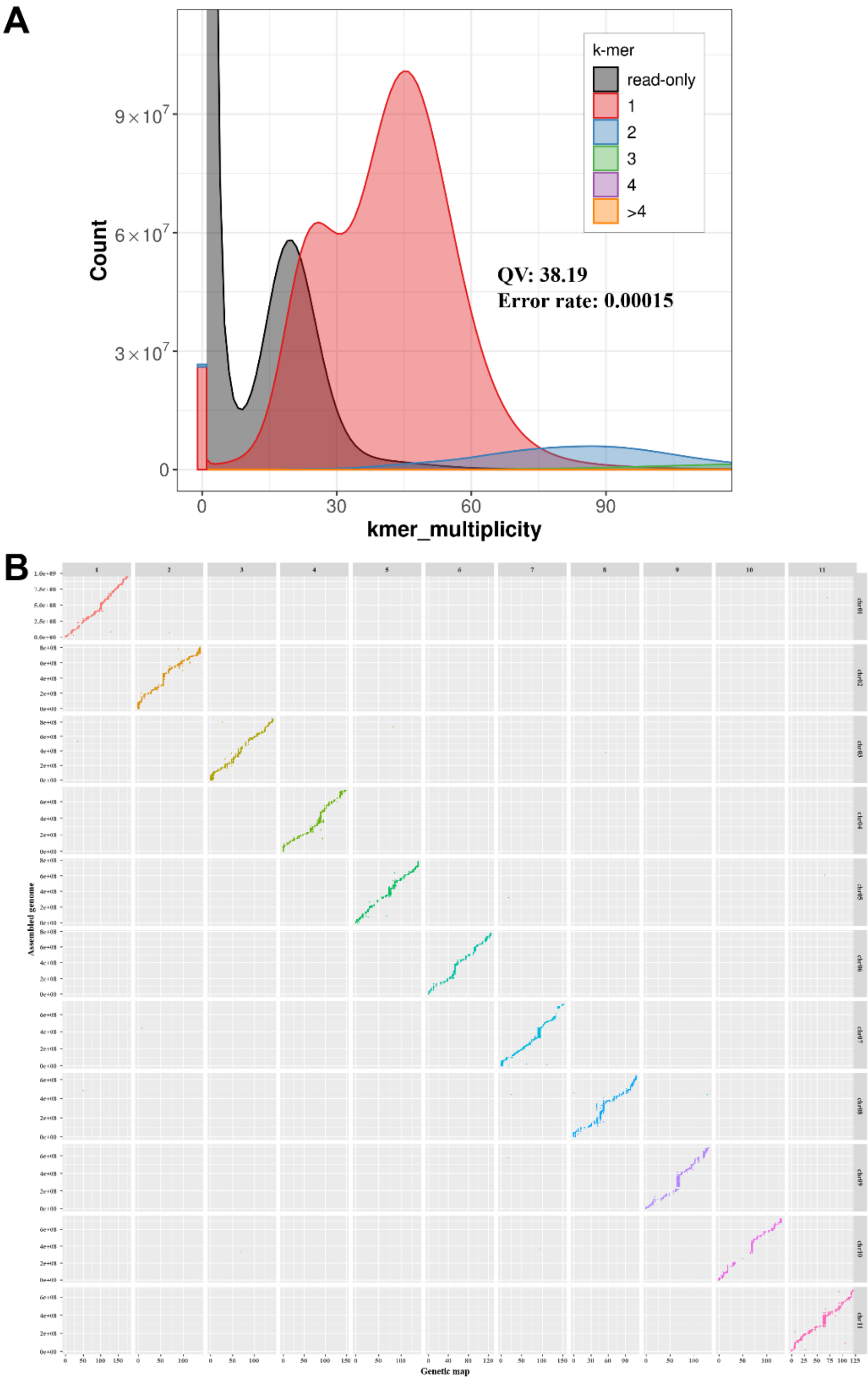

Supplemental Figure 4. Evaluation of the *P. orientalis* genome assembly. (A) Assessment of genome assembly using Merqury program. The plot displays the copy number spectrum with its quality value (QV) and base error rate. The *K*-mer size was set to 21. **(B)** The collinearity between genetic map and *P. orientalis* genome assembly.

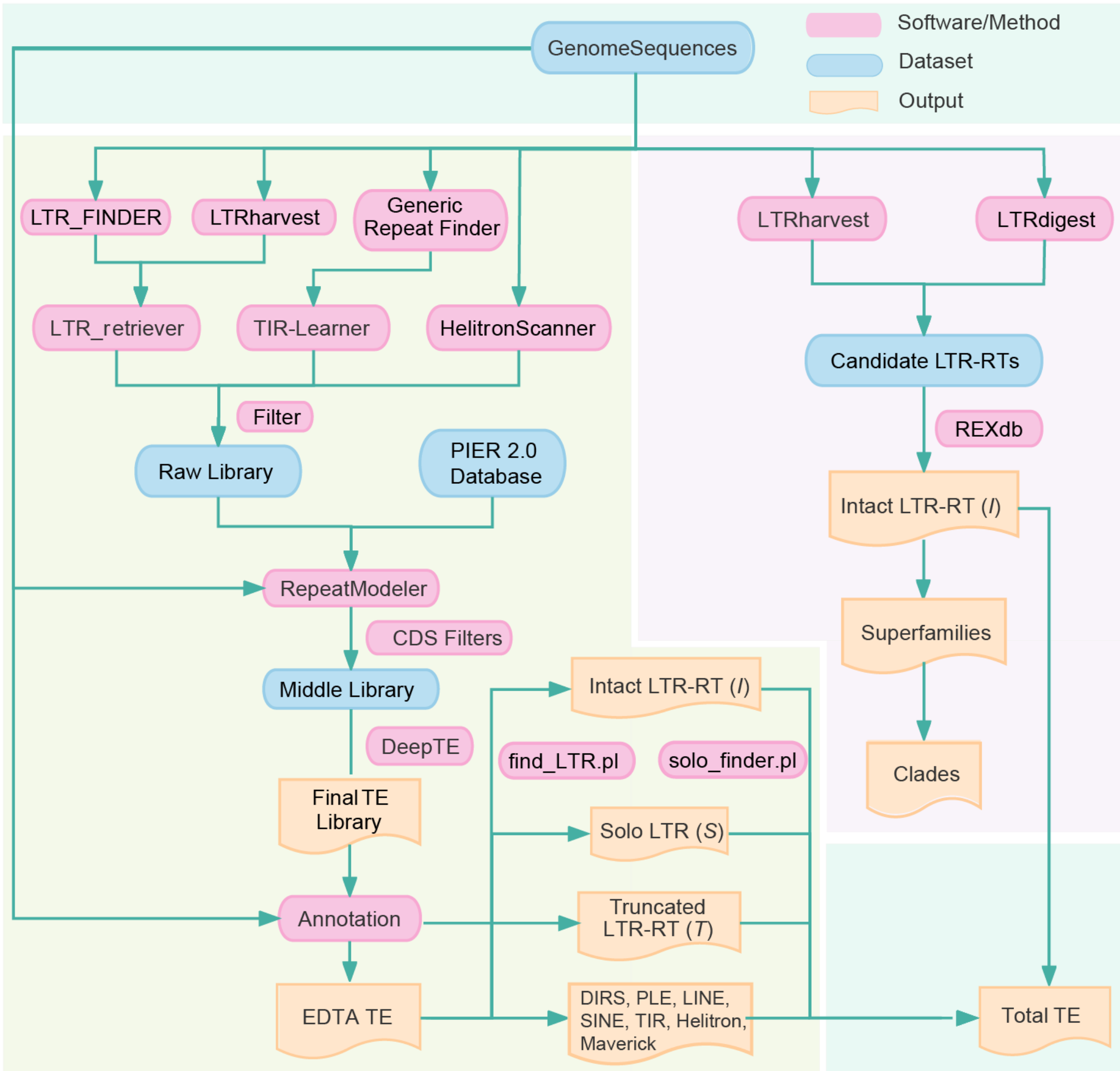

Supplemental Figure 5. Schematic workflow for the genome-wide transposable element (TE) annotation in gymnosperms.

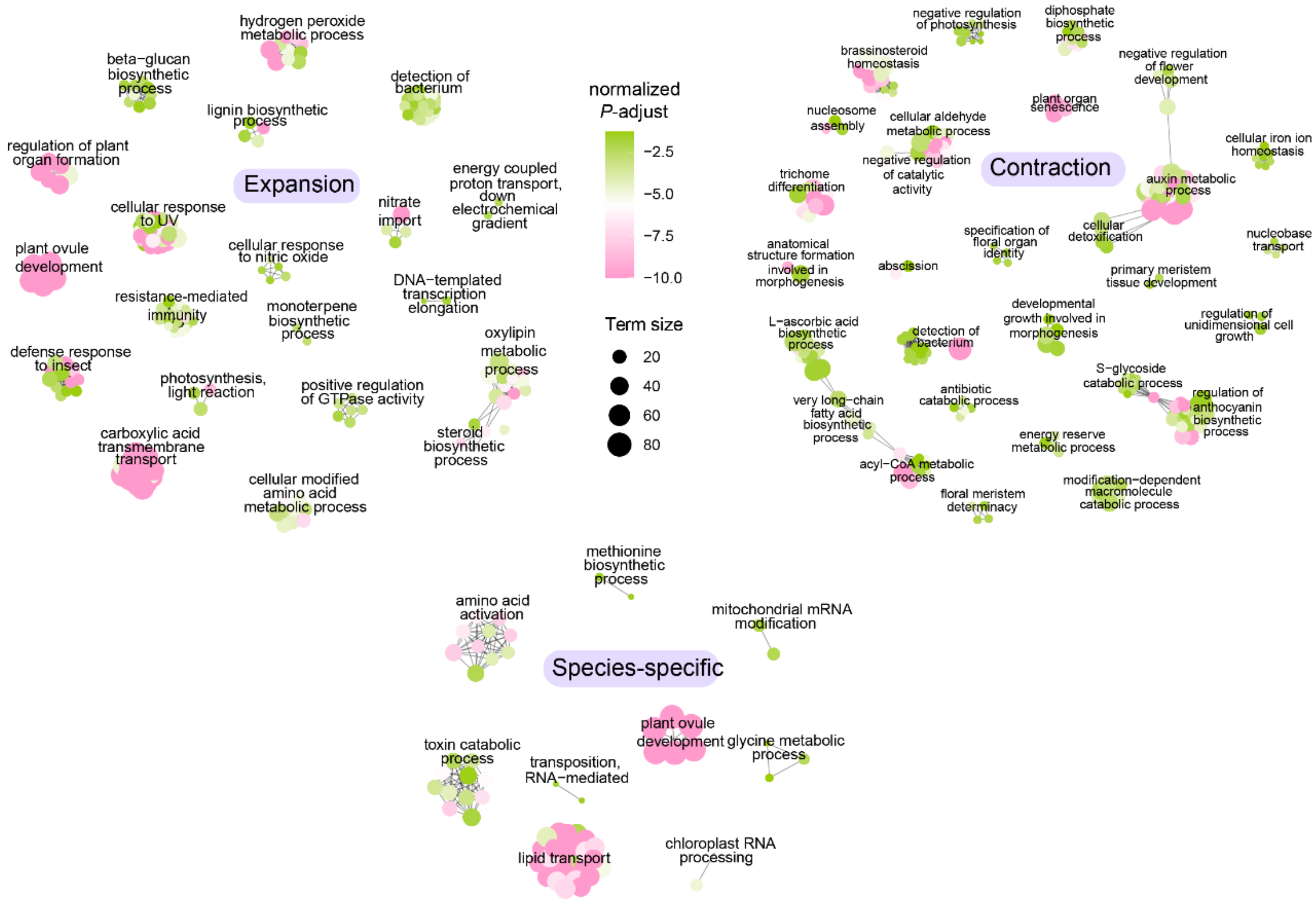

Supplemental Figure 6. Network and clustering of the significantly expanded, contracted and species-specific genes GO terms obtained through enrichment analysis.

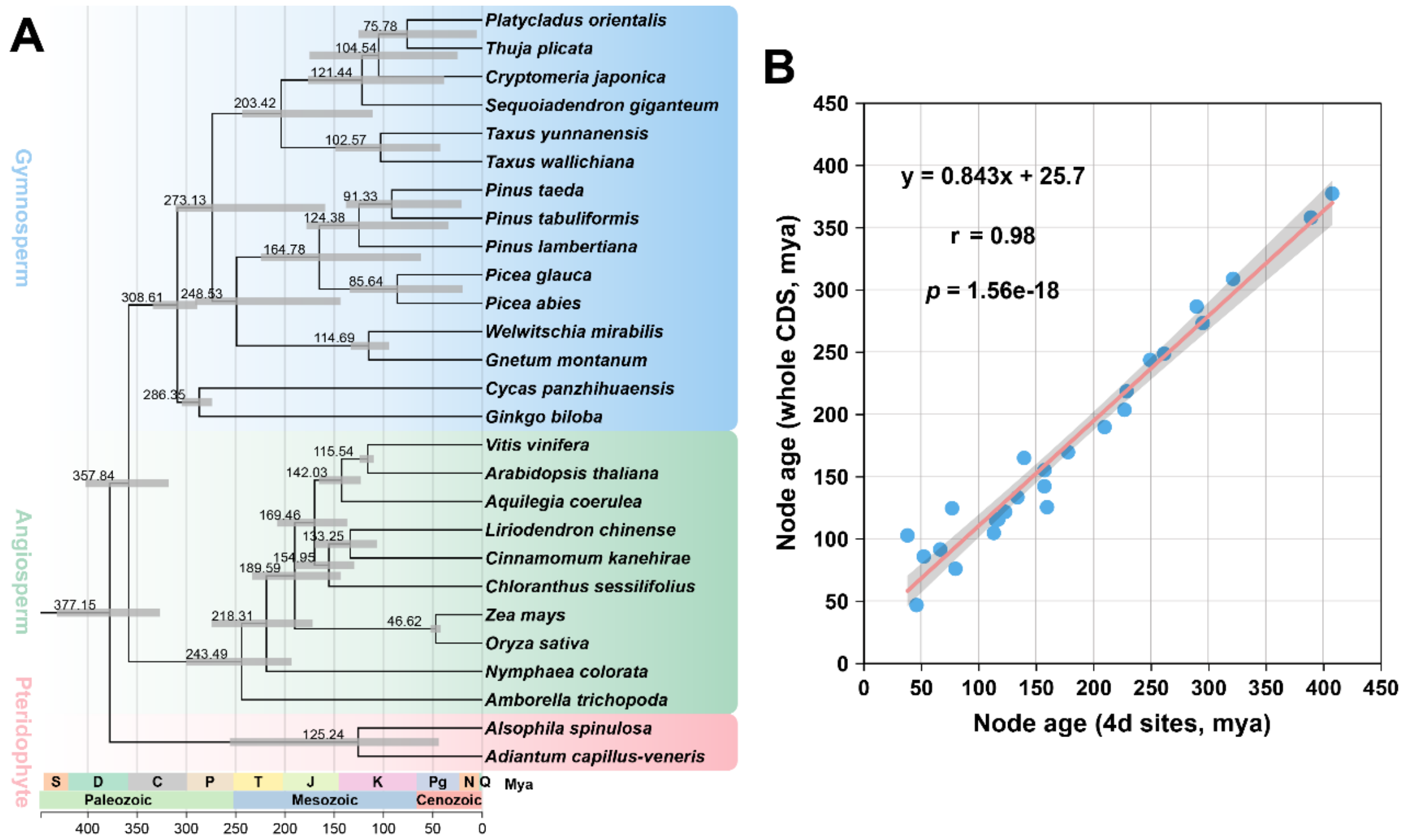

Supplemental Figure 7. Divergence time estimation among 27 representative plant species. (A) Divergence time tree obtained from the whole CDS dataset among 27 plants. The numbers and gray bars alongside the nodes represent the dated divergence times and associated 95% confidence intervals. S, Silurian; D, Devonian; C, Carboniferous; P, Permian; T, Triassic; J, Jurassic K, Cretaceous; Pg, Palaeogene; N, Neogene; Q, Quaternary; Mya, million years ago. **(B)** Correlation plot illustrating the node ages derived from the analyses of 4d sites and whole coding sequences (CDS).

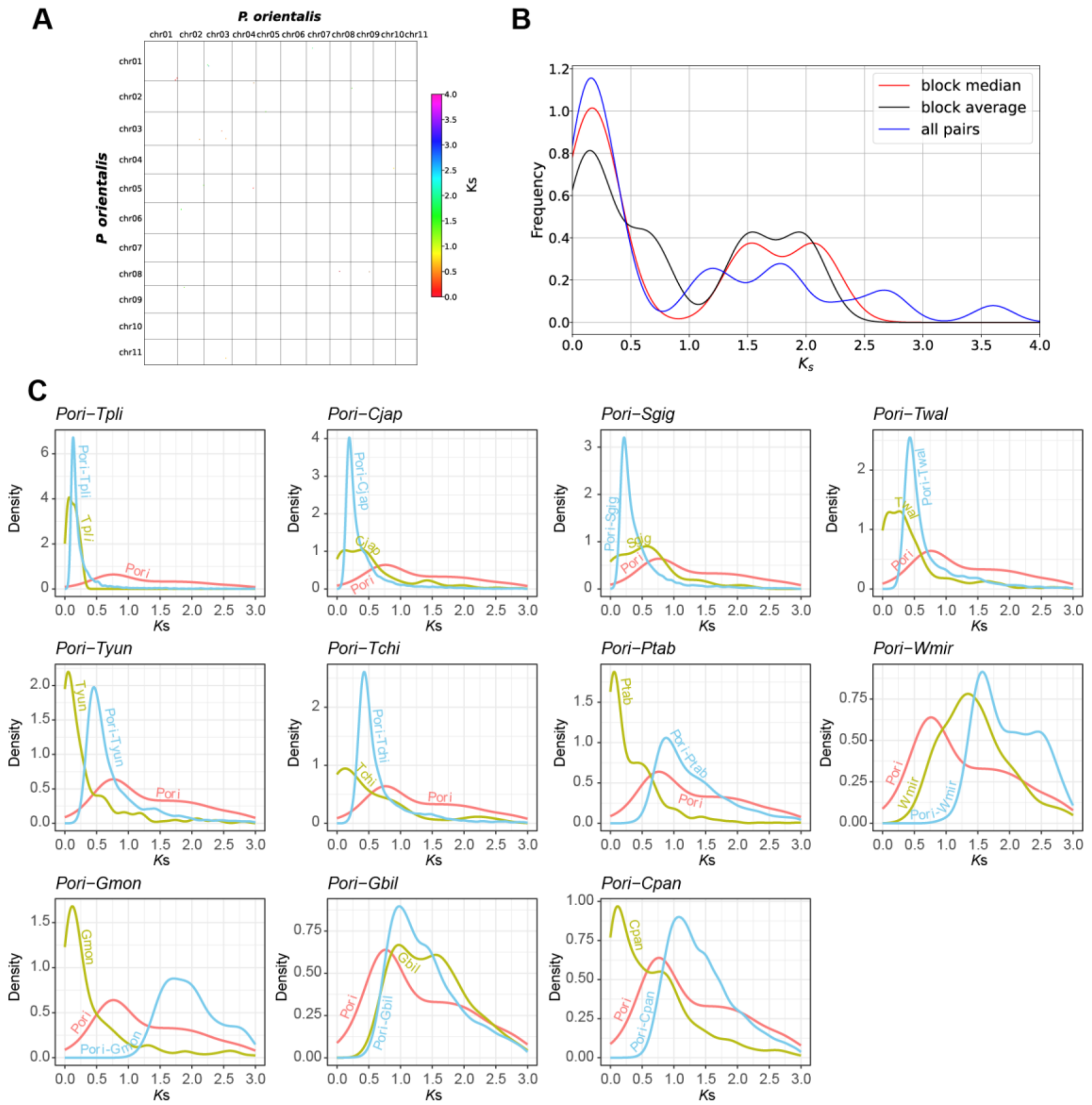

Supplemental Figure 8. Synteny and synonymous substitution rates (*K*s) analyses in *P. orientalis* determined by WGDI and MCScanX. (A) Dot plot of intra-genomic synteny analysis performed using WGDI. Syntenic blocks with at least five anchor pairs are displayed, with colors representing *K*s values. **(B)** Distribution of *K*s values for syntenic gene pairs and blocks derived from WGDI. **(C) Distribution of *K*s values for syntenic gene pairs within *P. orientalis* and between *P. orientalis* and 11 other gymnosperm species identified using MCScanX.** *Pori*: *Platycladus orientalis*; *Cjap*: *Cryptomeria japonica*; *Sgig*: *Sequoiadendron giganteum*; *Twal*: *Taxus wallichiana*; *Tyun*: *Taxus yunnanensis*; *Tchi*: *Taxus chinensis* var. *mairei*; *Ptab*: *Pinus tabuliformis*; *Wmir*: *Welwitschia mirabilis*; *Gmon*: *Gnetum montanum*; *Gbil*: *Ginkgo biloba*; *Cpan*: *Cycas panzhihuaensis*.

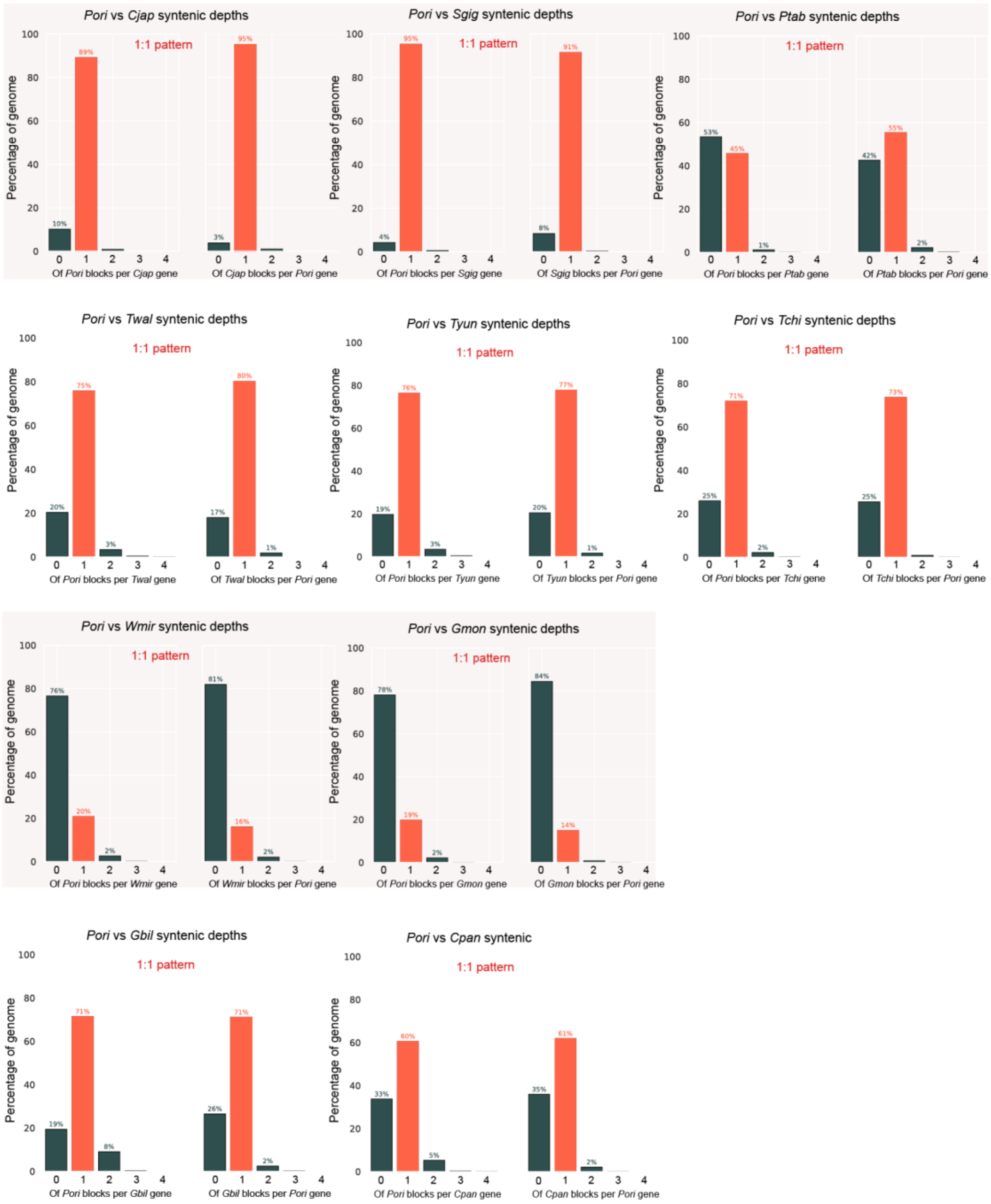

Supplemental Figure 9. Synteny depths analysis between *P. orientalis* and ten gymnosperm species. The distribution of one-to-n homologs (n = 0, 1, 2, 3, 4) from one species to other species was shown. The bar chart shows the ratio of the number of homologous genes in one species to the number of homologous genes in another species. The x-axis shows the number of corresponding homologous genes, and the y-axis shows the proportion of the genes to all genes. The red columns indicate the most abundant categories of synteny proportions. *Pori*: *Platycladus orientalis*; *Cjap*: *Cryptomeria japonica*; *Sgig*: *Sequoiadendron giganteum*; *Twal*: *Taxus wallichiana*; *Tyun*: *Taxus yunnanensis*; *Tchi*: *Taxus chinensis* var. *mairei*; *Ptab*: *Pinus tabuliformis*; *Wmir*: *Welwitschia mirabilis*; *Gmon*: *Gnetum montanum*; *Gbil*: *Ginkgo biloba*; *Cpan*: *Cycas panzhihuaensis*.

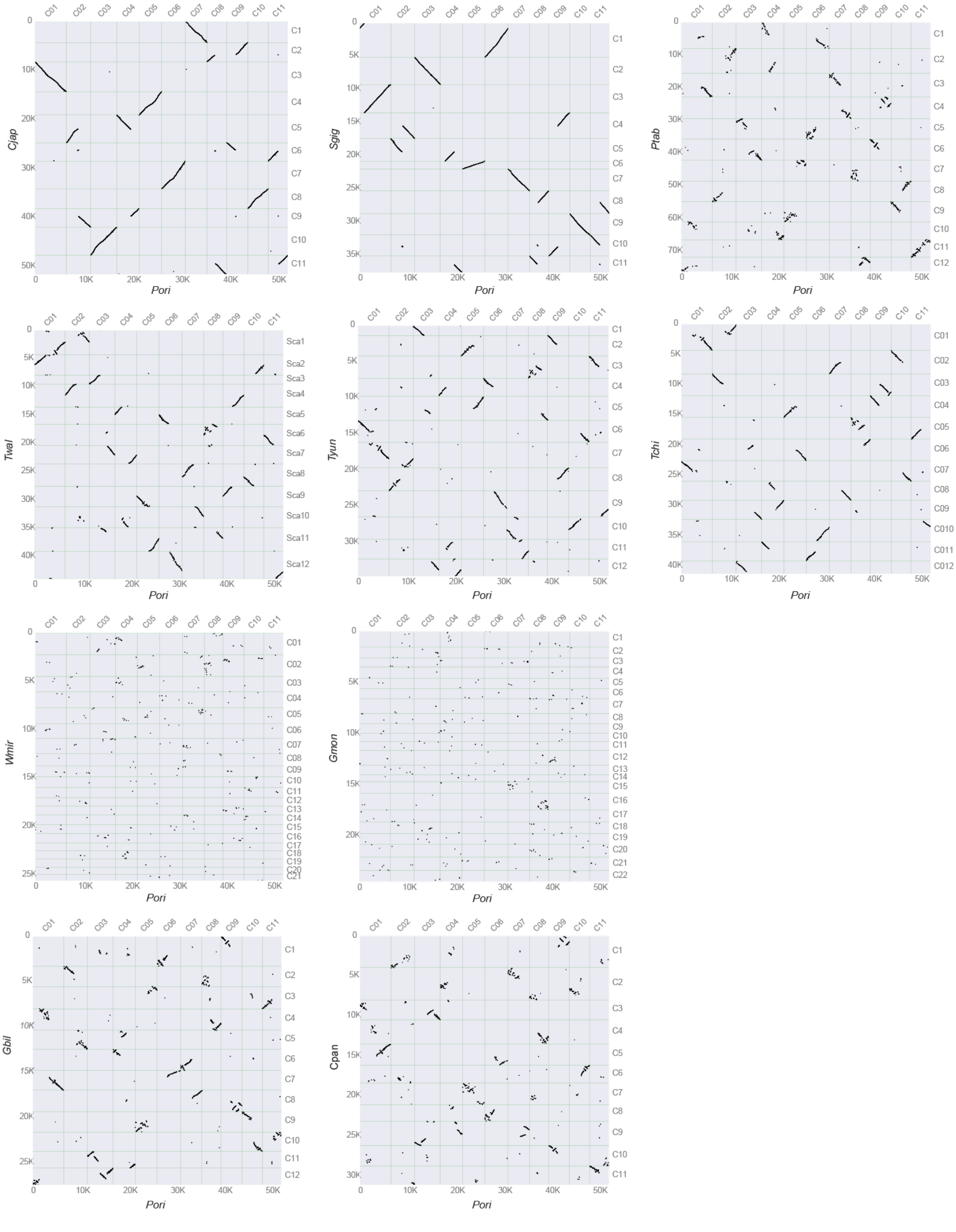

Supplemental Figure 10. Intergenomic dot plot of synteny between *P. orientalis* and ten gymnosperm species. *Pori*: *Platycladus orientalis*; *Cjap*: *Cryptomeria japonica*; *Sgig*: *Sequoiadendron giganteum*; *Twal*: *Taxus wallichiana*; *Tyun*: *Taxus yunnanensis*; *Tchi*: *Taxus chinensis* var. *mairei*; *Ptab*: *Pinus tabuliformis*; *Wmir*: *Welwitschia mirabilis*; *Gmon*: *Gnetum montanum*; *Gbil*: *Ginkgo biloba*; *Cpan*: *Cycas panzhihuaensis*.

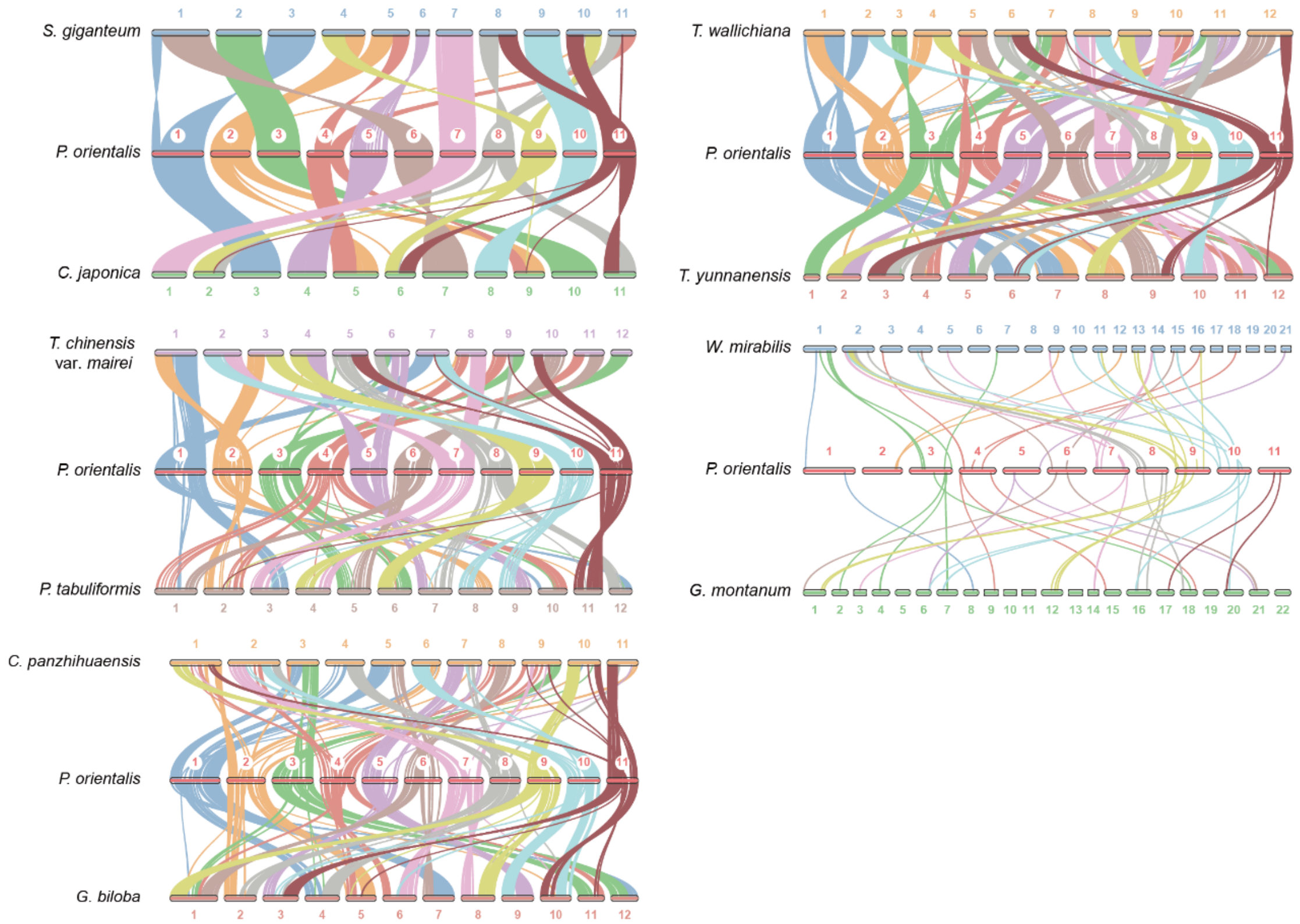

Supplemental Figure 11. Collinearity between the chromosomes of *P. orientalis* and ten gymnosperm species. Lines depict homologous genome blocks.

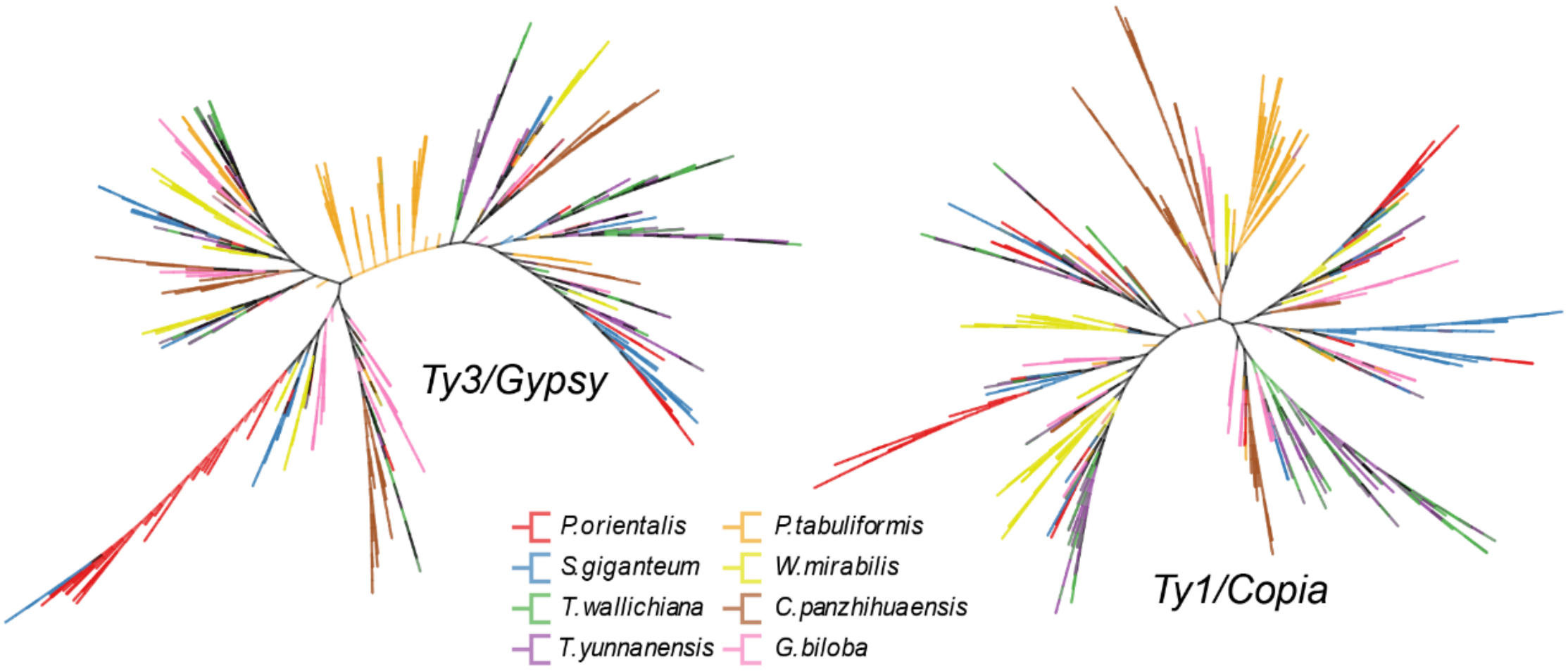

Supplemental Figure 12. Phylogenetic trees of *Ty3*/*Gypsy* and *Ty1*/*Copia* LTR-RT reverse transcriptase domains.

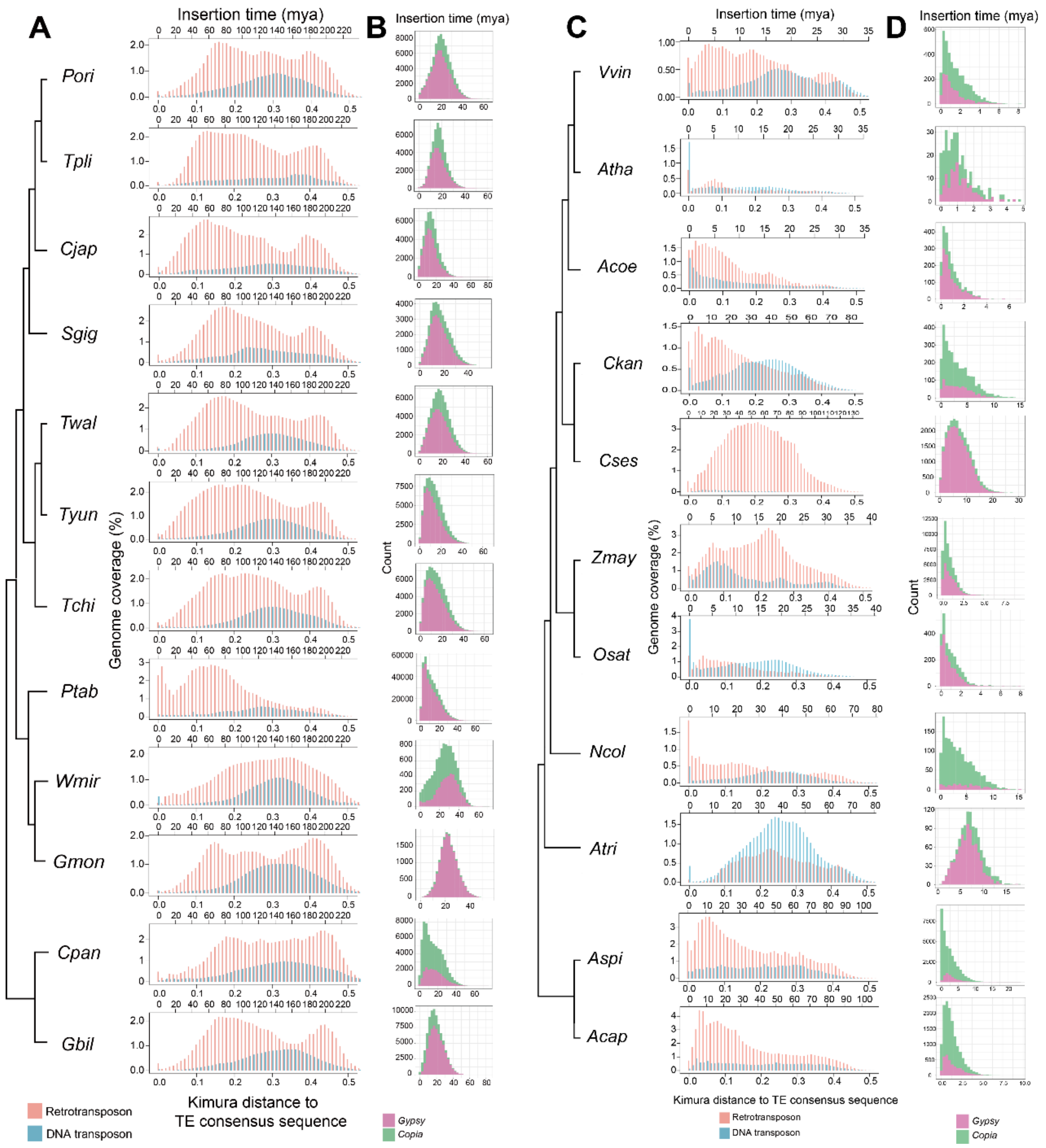

Supplemental Figure 13. Insertion time of (A) all TE types, (B) intact LTR-RT as detected in 12 gymnosperms, (C) all TE types and (D) intact LTR-RT in 11 angiosperms and pteridophytes. *Pori*: *Platycladus orientalis*; *Tpli*: *Thuja plicata*; *Cjap*: *Cryptomeria japonica*; *Sgig*: *Sequoiadendron giganteum*; *Twal*: *Taxus wallichiana*; *Tyun*: *Taxus yunnanensis*; *Tchi*: *Taxus chinensis* var. *mairei*; *Ptab*: *Pinus tabuliformis*; *Wmir*: *Welwitschia mirabilis*; *Gmon*: *Gnetum montanum*; *Cpan*: *Cycas panzhihuaensis*; *Gbil*: *Ginkgo biloba. Vvin*: *Vitis vinifera*; *Atha*: *Arabidopsis thaliana*; *Acoe*: *Aquilegia coerulea*; *Ckan*: *Cinnamomum kanehirae*; *Cses*: *Chloranthus sessilifolius*; *Zmays*: *Zea mays*; *Osat*: *Oryza sativa*; *Ncol*: *Nymphaea colorata*; *Atri*: *Amborella trichopoda*; *Aspi*: *Alsophila spinulosa*; *Acap*: *Adiantum capillus-veneris.*

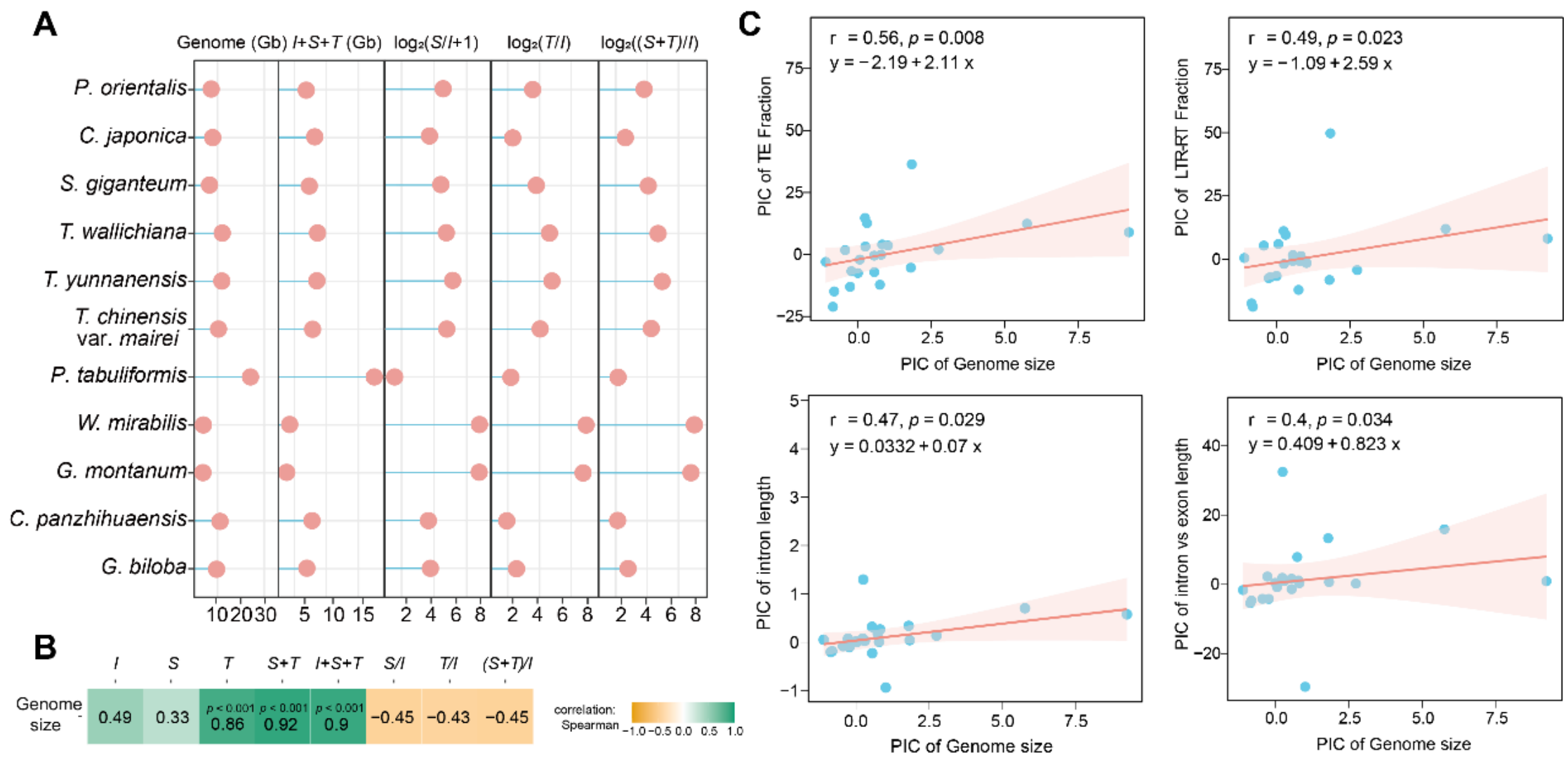

Supplemental Figure 14. Correlations between genome size and genomic features. (A) Dynamics of LTR-RTs in gymnosperms. LTR-RT generation (*S*+*T*+*I*) and LTR-RT removal rates (*S*/*I*, *T*/*I*, and (*S*+*T*)/*I*) are displayed in columns. **(B)** Heatmap showing the correlations between LTR-RT accumulation, removal, and genome size across 11 gymnosperms. *I*: intact LTR-RT, *S*: solo-LTR, *T*: truncated LTR-RT. **(C)** Correlation between genome size and TE fraction, LTR retrotransposon fraction, intron length and the ratio of intron to exon length, while accounting for phylogenetic non-independence among 23 species.

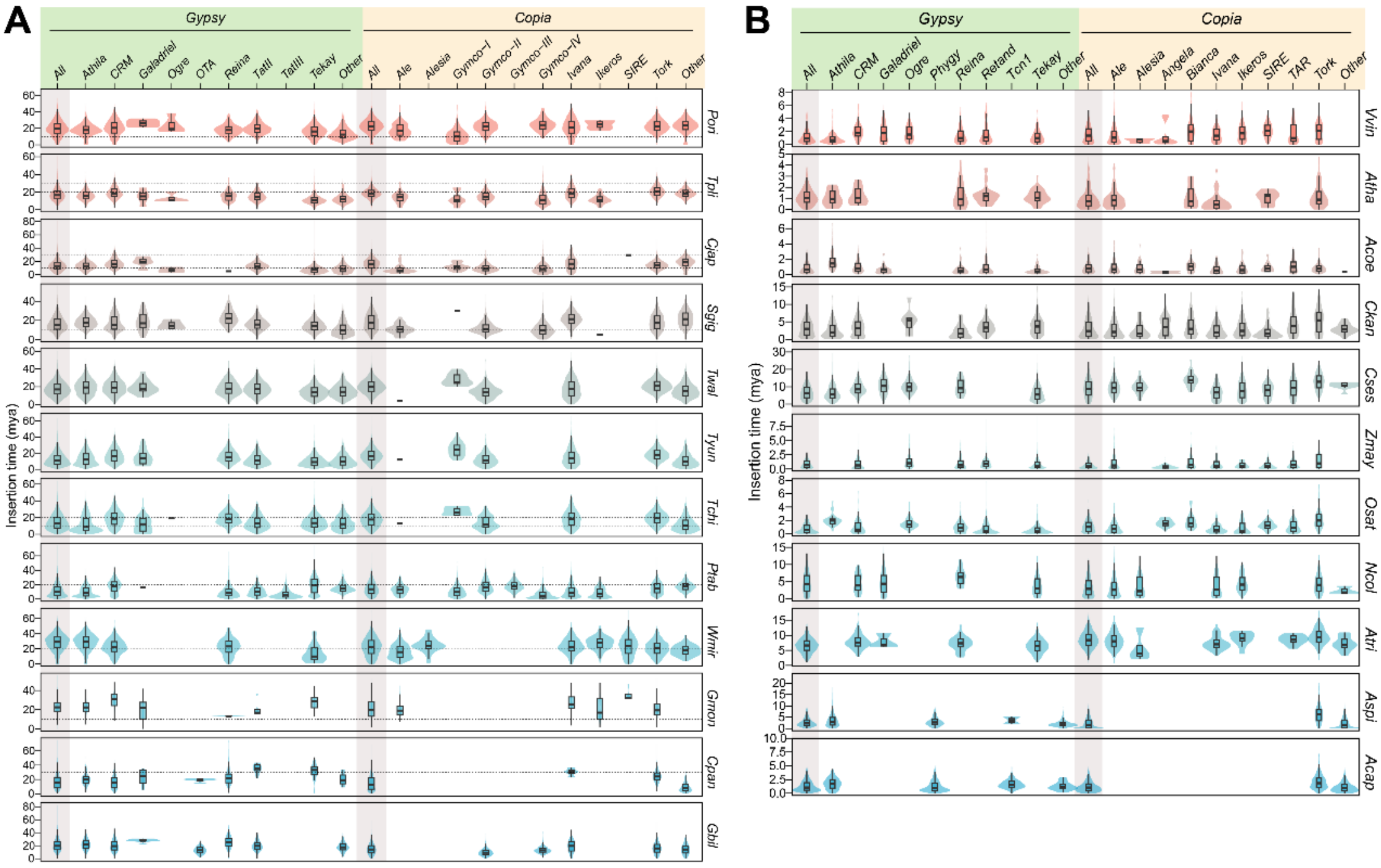

Supplemental Figure 15. Evolutionary dynamics of intact LTR-RT families in (A) 12 gymnosperms and (B) 11 angiosperms and pteridophytes. *Pori*: *Platycladus orientalis*; *Tpli*: *Thuja plicata*; *Cjap*: *Cryptomeria japonica*; *Sgig*: *Sequoiadendron giganteum*; *Twal*: *Taxus wallichiana*; *Tyun*: *Taxus yunnanensis*; *Tchi*: *Taxus chinensis* var. *mairei*; *Ptab*: *Pinus tabuliformis*; *Wmir*: *Welwitschia mirabilis*; *Gmon*: *Gnetum montanum*; *Cpan*: *Cycas panzhihuaensis*; *Gbil*: *Ginkgo biloba*. *Vvin*: *Vitis vinifera*; *Atha*: *Arabidopsis thaliana*; *Acoe*: *Aquilegia coerulea*; *Ckan*: *Cinnamomum kanehirae*; *Cses*: *Chloranthus sessilifolius*; *Zmays*: *Zea mays*; *Osat*: *Oryza sativa*; *Ncol*: *Nymphaea colorata*; *Atri*: *Amborella trichopoda*; *Aspi*: *Alsophila spinulosa*; *Acap*: *Adiantum capillus-veneris.*

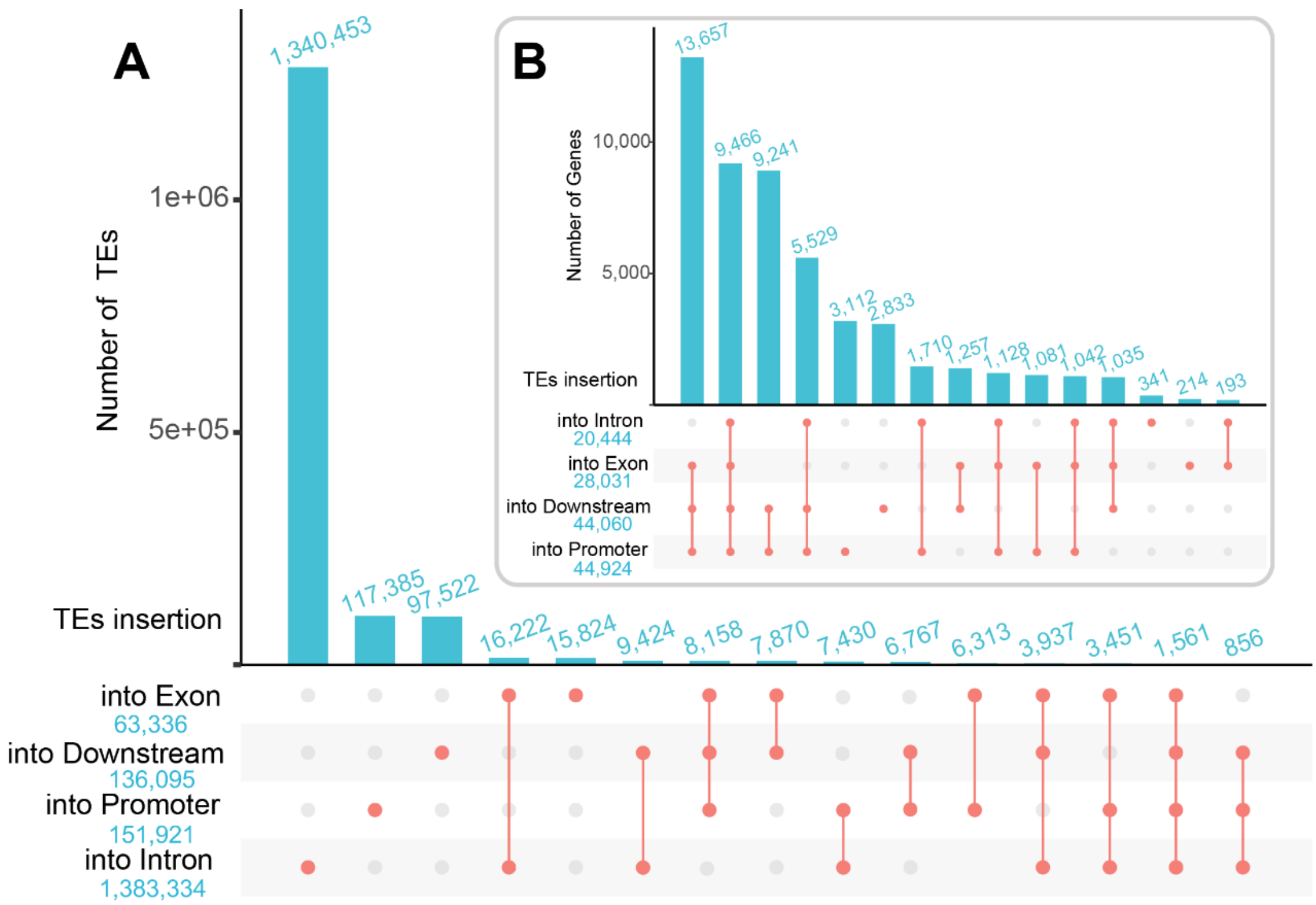

Supplemental Figure 16. UpSet plot of TE insertions relative to gene positions in *P. orientalis*. (A) UpSet plot of the TE counts for different types of insertions. **(B)** UpSet plot of the gene counts for different types of insertions.

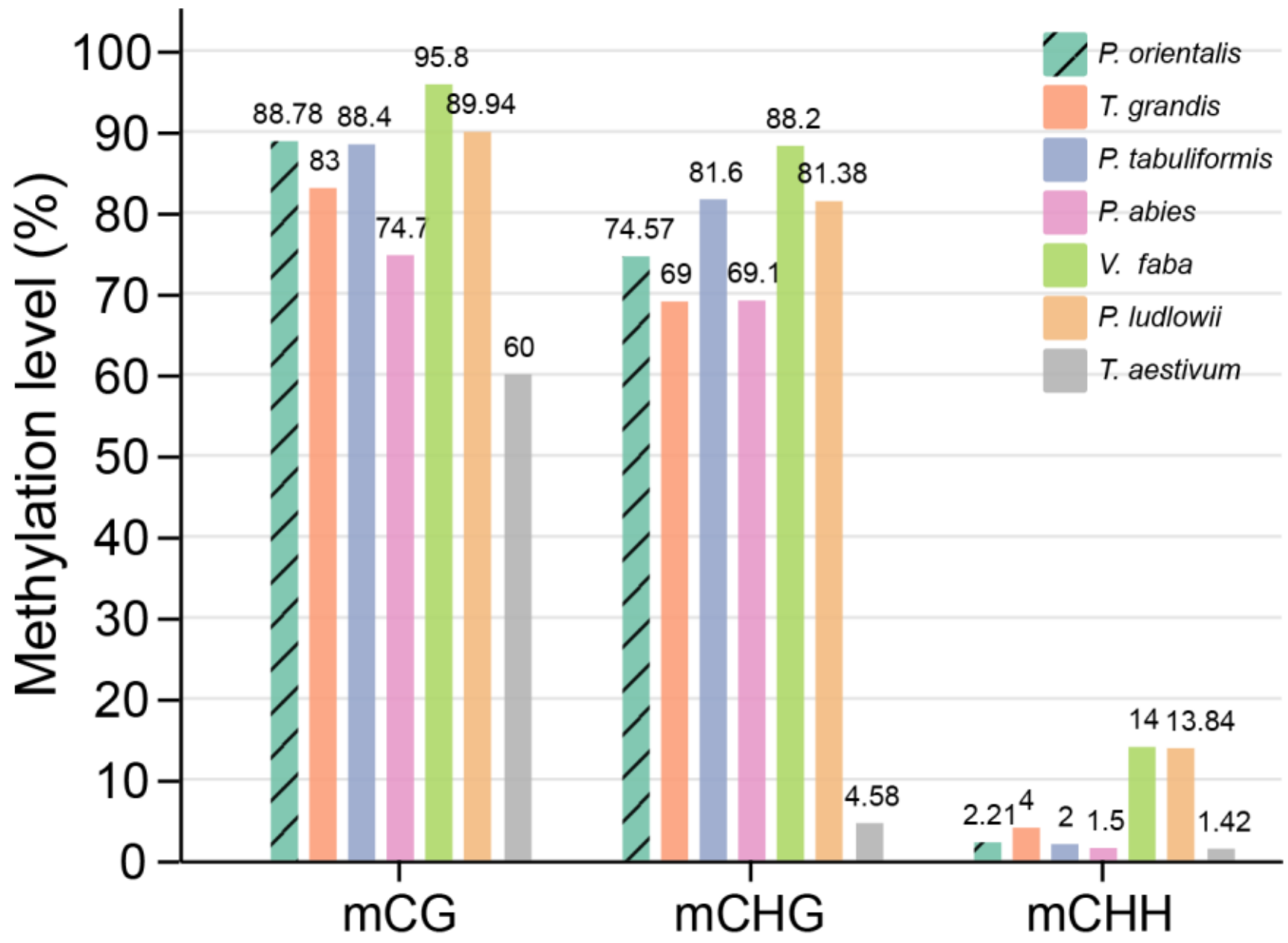

Supplemental Figure 17. Genome-wide DNA methylation level of *P. orientalis* and 6 other plant genomes with high TE content (>60%) and genome size (>10G).

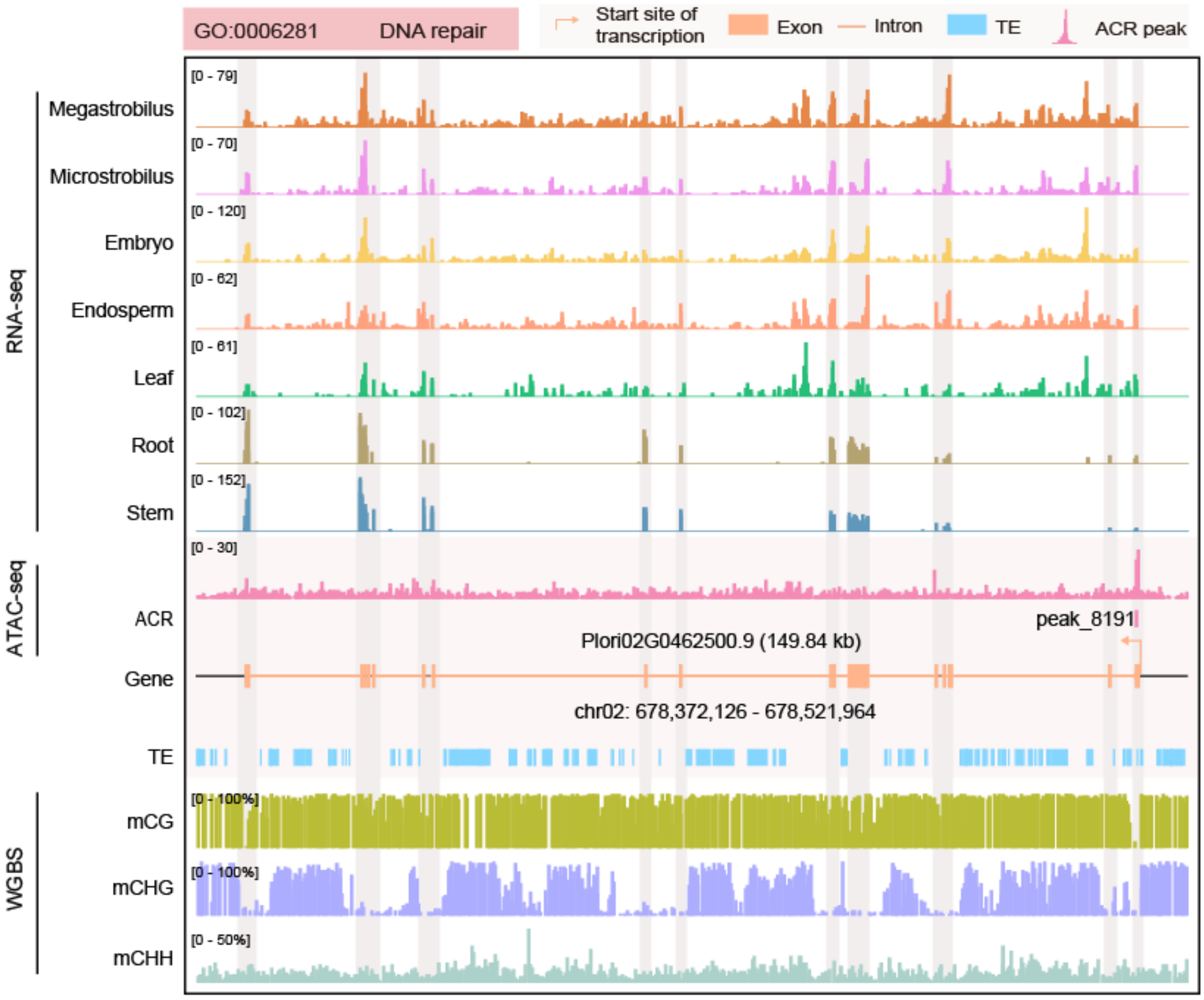

Supplemental Figure 18. The IGV presentation of expression, chromatin accessibility, and methylation levels in one ultra-long coding gene, exceeding 100 kb and harboring TE insertions.

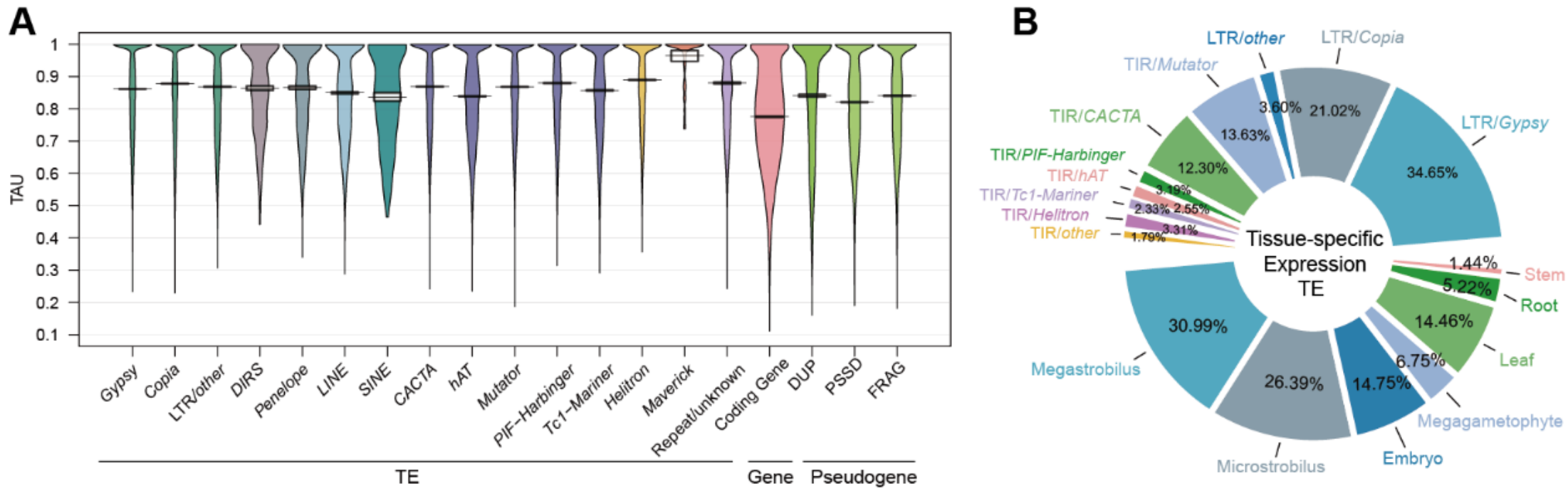

Supplemental Figure 19. The tissue-specific expression of TE in *P. orientalis*. (A) Tissue-preferential expression patterns of TE superfamilies, coding genes, and pseudogenes. Tau values closer to 1 indicate higher tissue-specific expression, while those closer to 0 indicate constitutive expression, with a tau of 1 representing complete tissue-specific expression. **(B)** Tissue-specific expression patterns of each superfamily of TE in five tissues.

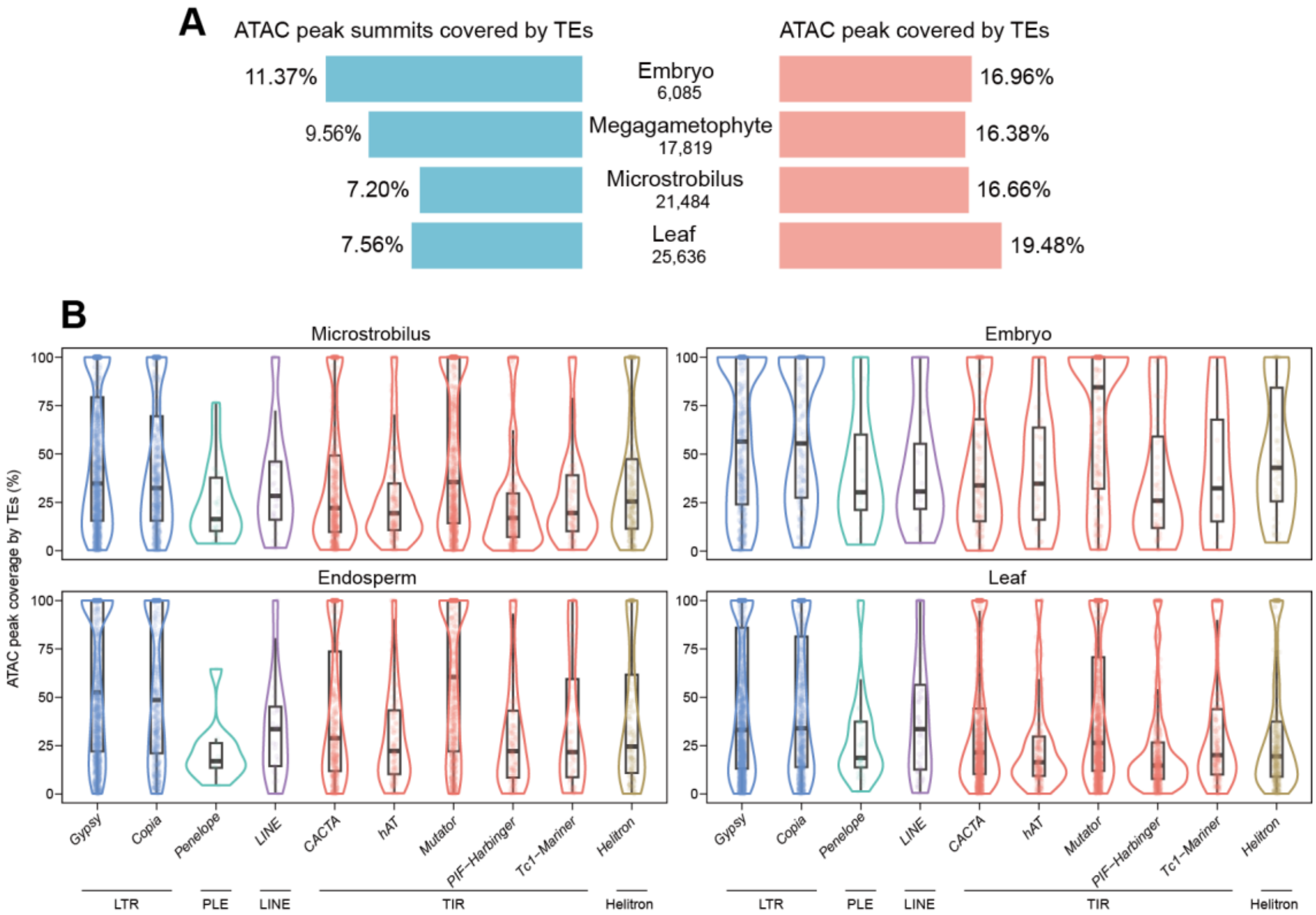

Supplemental Figure 20. The association between TEs and accessible chromatin regions (ACRs) in *P. orientalis*. (A) The total number of annotated ACRs in four tissues and the proportion of ACRs covered by TEs. **(B)** The coverage of ACRs by different TE superfamilies varies among the four organizations.

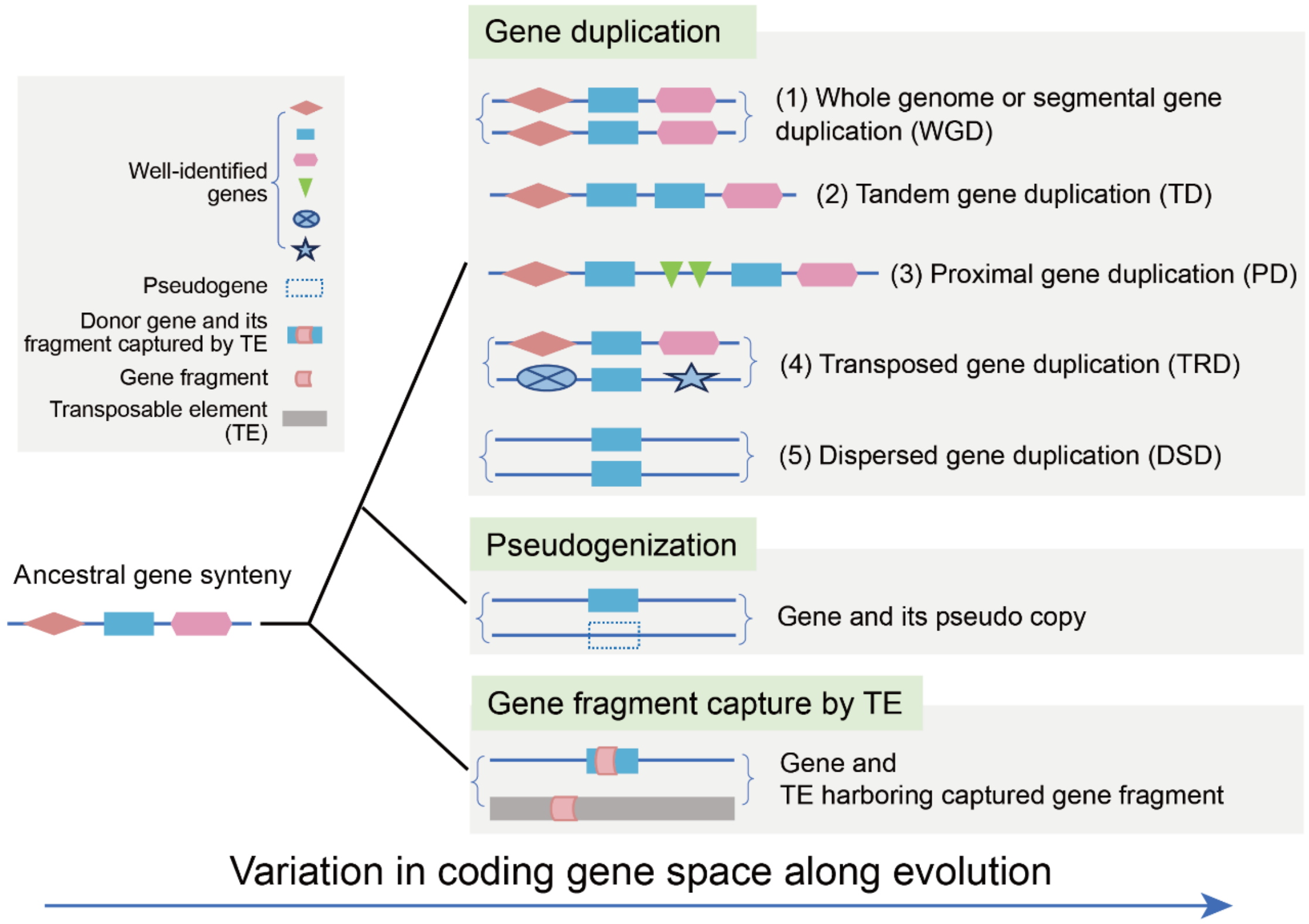

Supplemental Figure 21. Schematic illustration of evolutionary variations in the coding gene space.

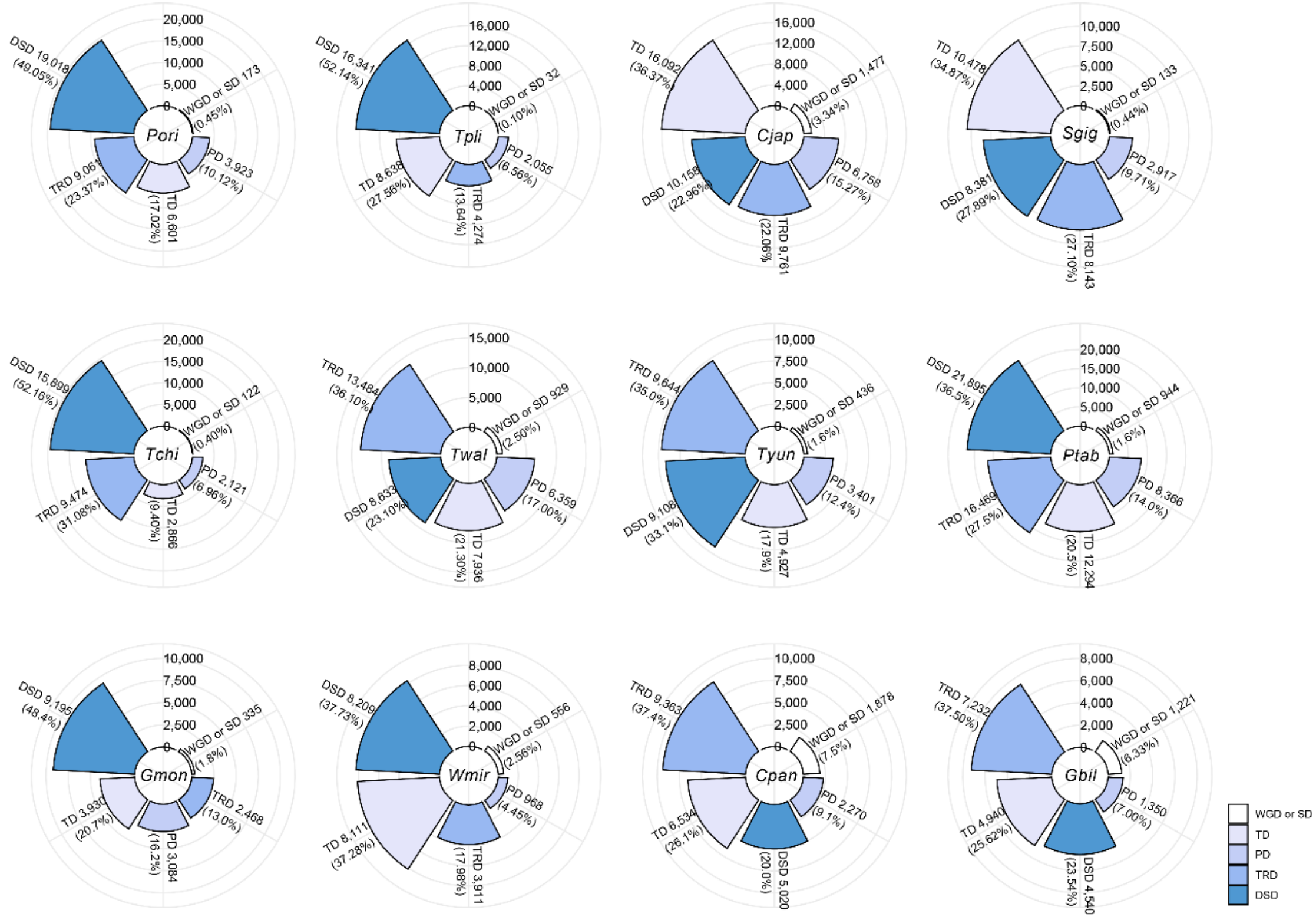

Supplemental Figure 22. The number of genes (excluding TE-associated genes) derived from different duplication modes in 12 gymnosperms. The priority of the duplicate genes is as follows: WGD or SD > TD > PD > TRD > DSD. WGD: whole-genome duplication, SD: segmental duplication, TD: tandem duplication, PD: proximal duplication, TRD: transposed duplication, DSD: dispersed duplication. *Pori*: *Platycladus orientalis*; *Tpli*: *Thuja plicata*; *Cjap*: *Cryptomeria japonica*; *Sgig*: *Sequoiadendron giganteum*; *Twal*: *Taxus wallichiana*; *Tyun*: *Taxus yunnanensis*; *Tchi*: *Taxus chinensis* var. *mairei*; *Ptab*: *Pinus tabuliformis*; *Wmir*: *Welwitschia mirabilis*; *Gmon*: *Gnetum montanum*; *Cpan*: *Cycas panzhihuaensis*; *Gbil*: *Ginkgo biloba.*

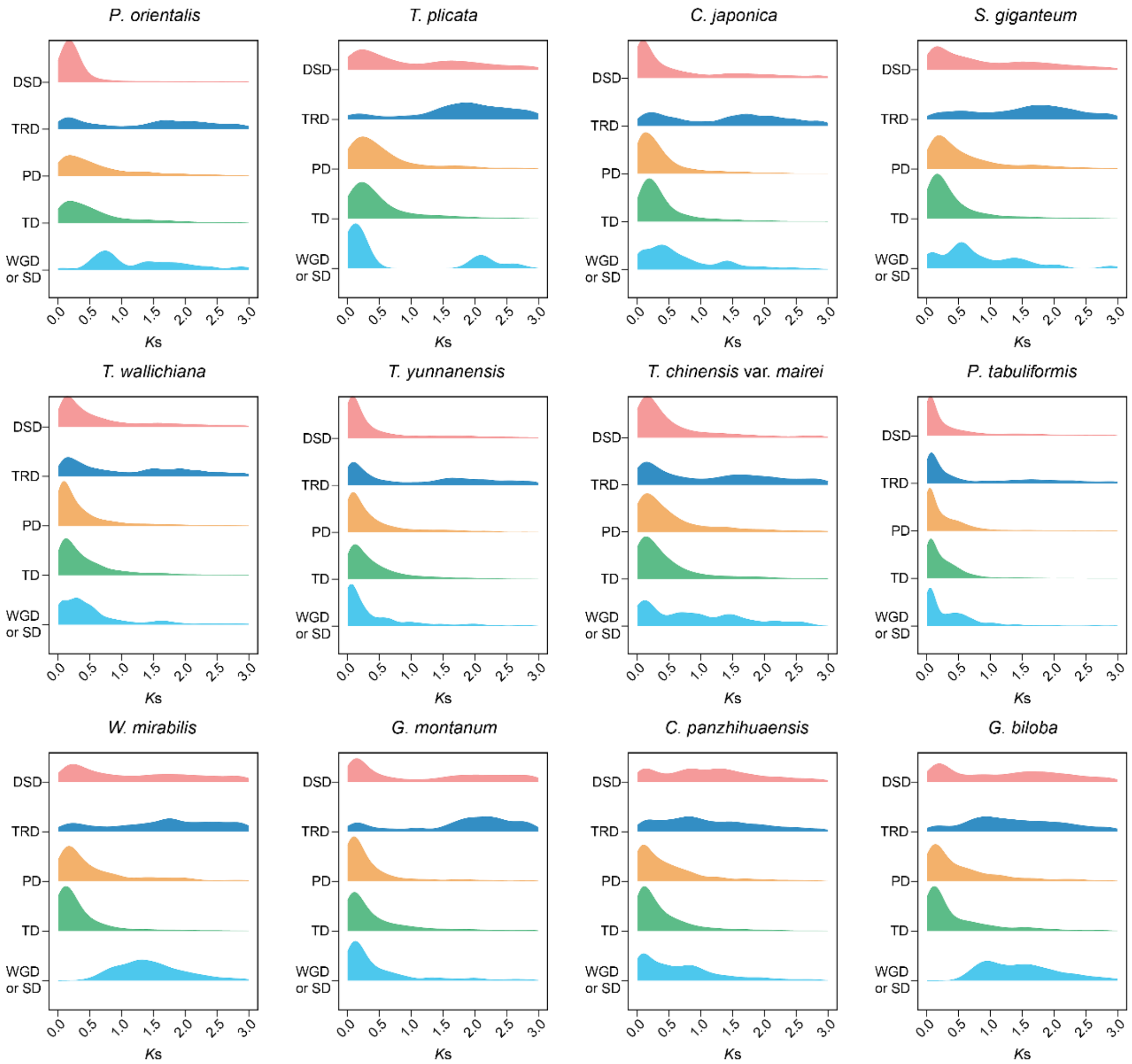

Supplemental Figure 23. *Ks* distributions of duplicate gene pairs derived from 5 duplication modes in 12 gymnosperm species. WGD: whole-genome duplication, SD: segmental duplication, TD: tandem duplication, PD: proximal duplication, TRD: transposed duplication, DSD: dispersed duplication.

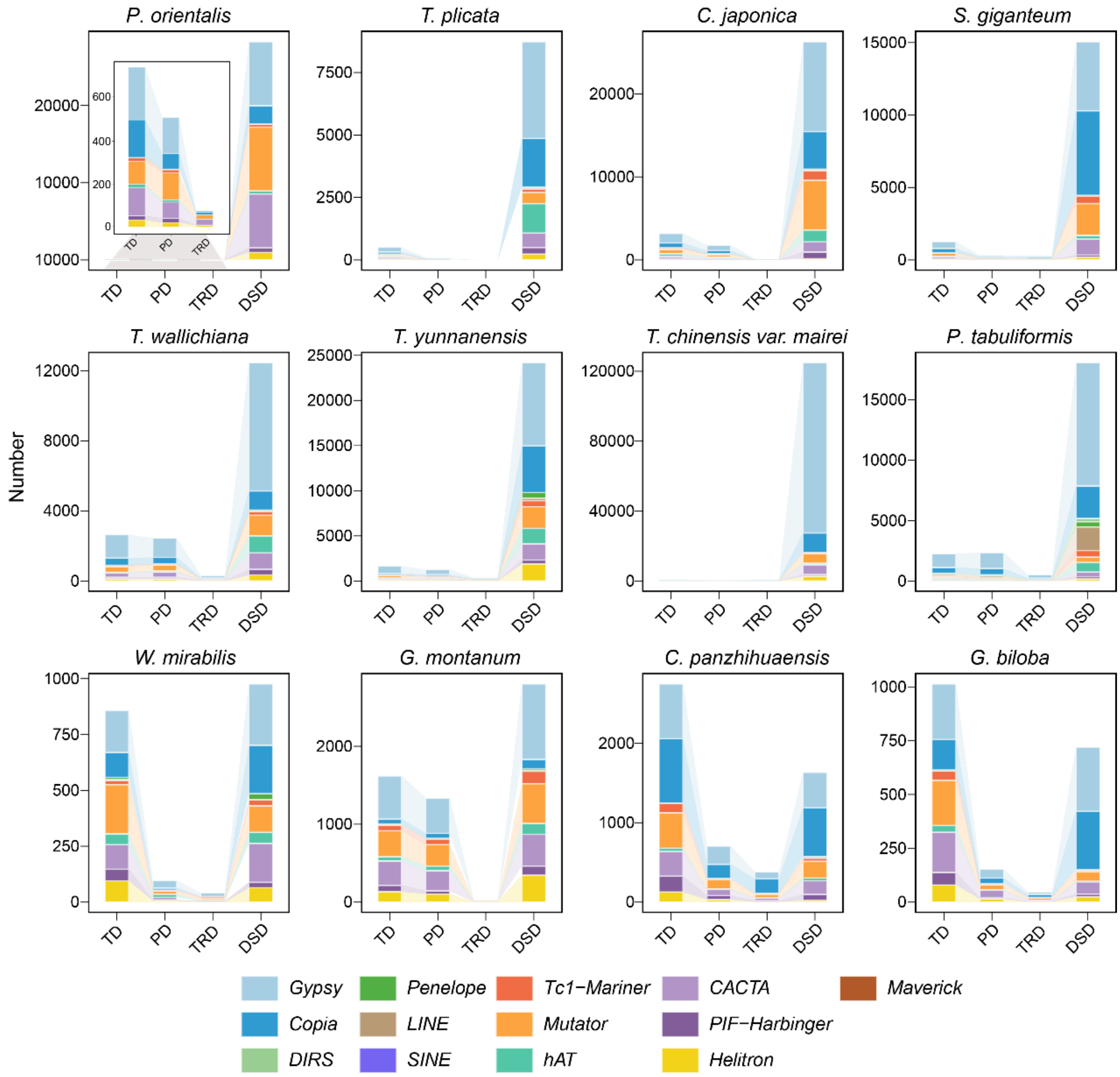

Supplemental Figure 24. Number of TE superfamilies mediating TE-recombination gene duplication. TD: tandem duplication, PD: proximal duplication, TRD: transposed duplication, DSD: dispersed duplication.

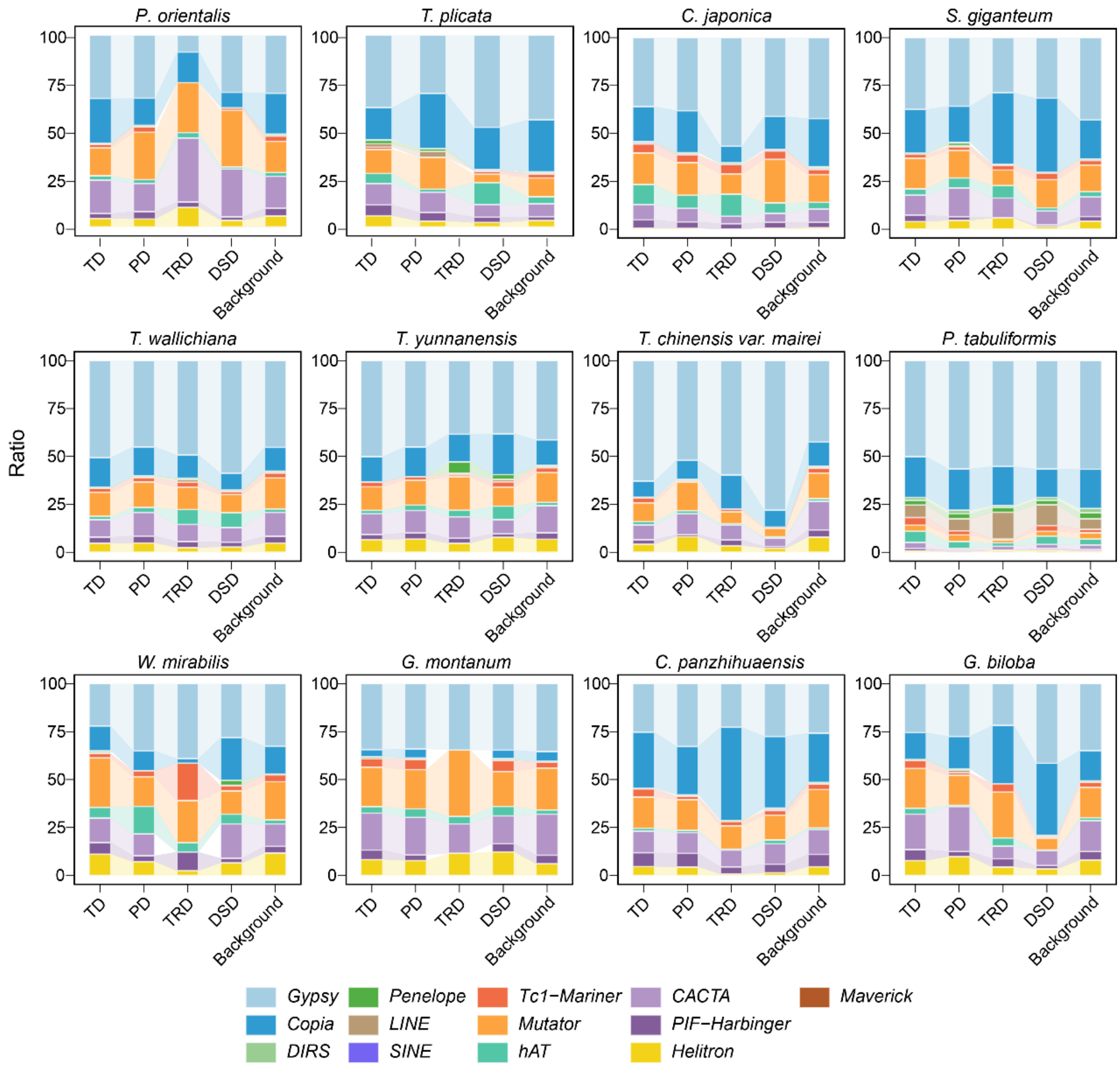

Supplemental Figure 25. Proportion of different TE superfamilies mediating TE-recombination gene duplication. The background column represents the proportion of different TE subfamilies out of the total number of TEs in the genome. TD: tandem duplication, PD: proximal duplication, TRD: transposed duplication, DSD: dispersed duplication.

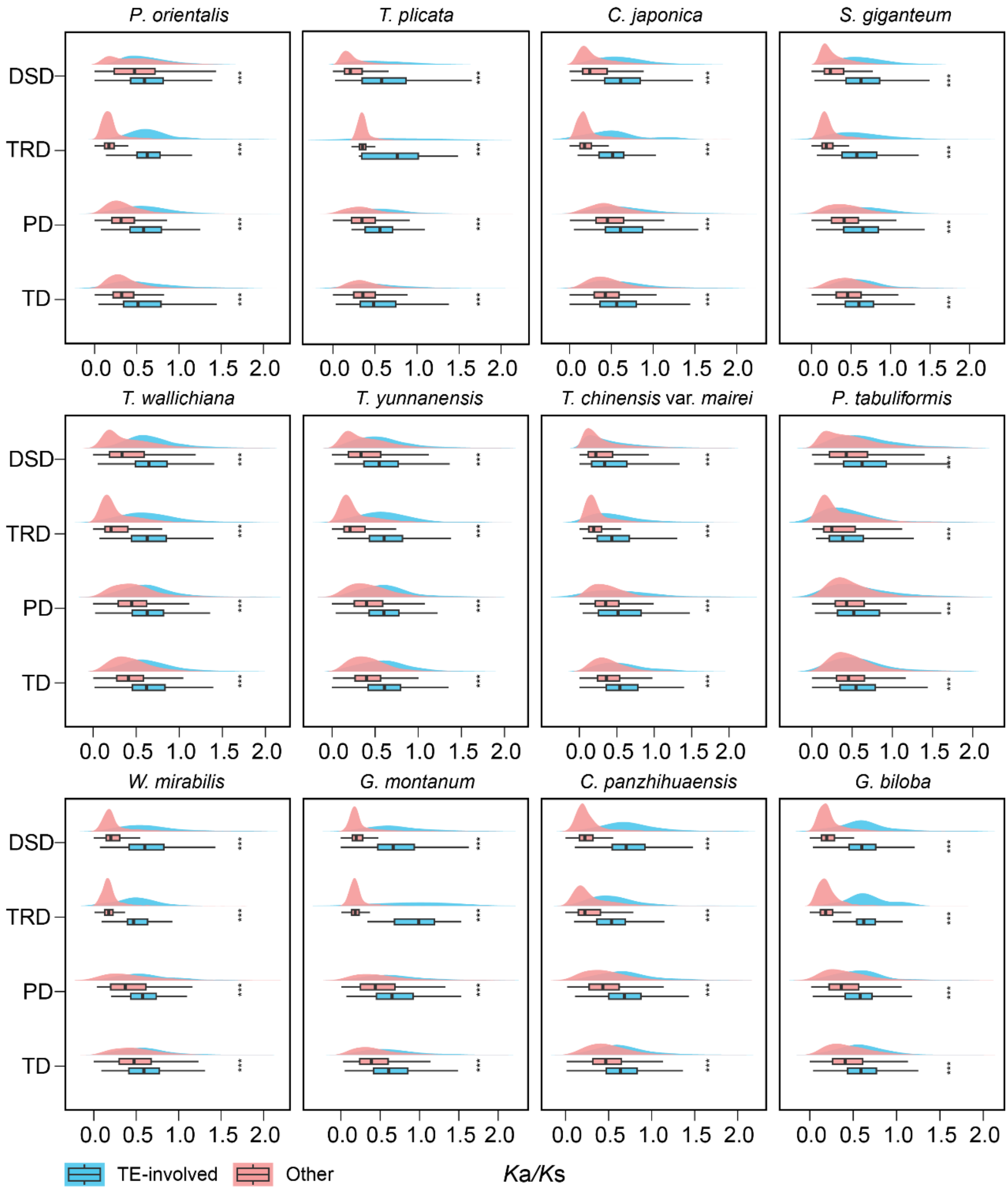

Supplemental Figure 26. Comparison of the *Ka*/*Ks* values between TE-mediated gene pairs and those of other gene pairs across 12 different species. All the data demonstrate that the former showed higher *Ka*/*Ks* values compared to the latter, and upon performing the Mann-Whitney U test: ∗∗∗, $p < 0.001$.

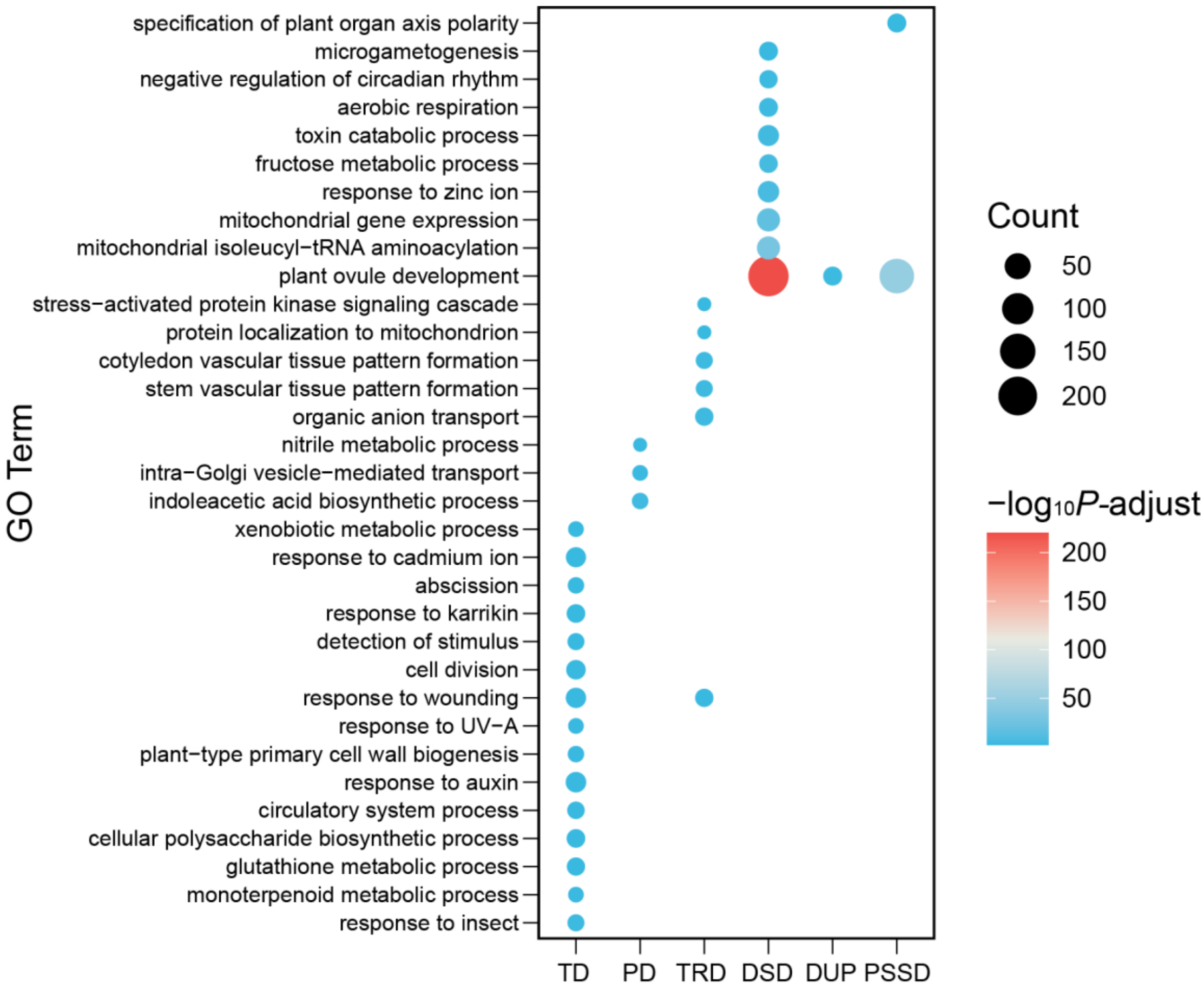

Supplemental Figure 27. Functional enrichment of genes from TE-mediated four classes of small-scale gene duplication and two classes of pseudogenization in *P. orientalis* (adjusted *p*-value < 0.01). TD: tandem duplication, PD: proximal duplication, TRD: transposed duplication, DSD: dispersed duplication, DUP: duplicated pseudogene, PSSD: processed pseudogene.

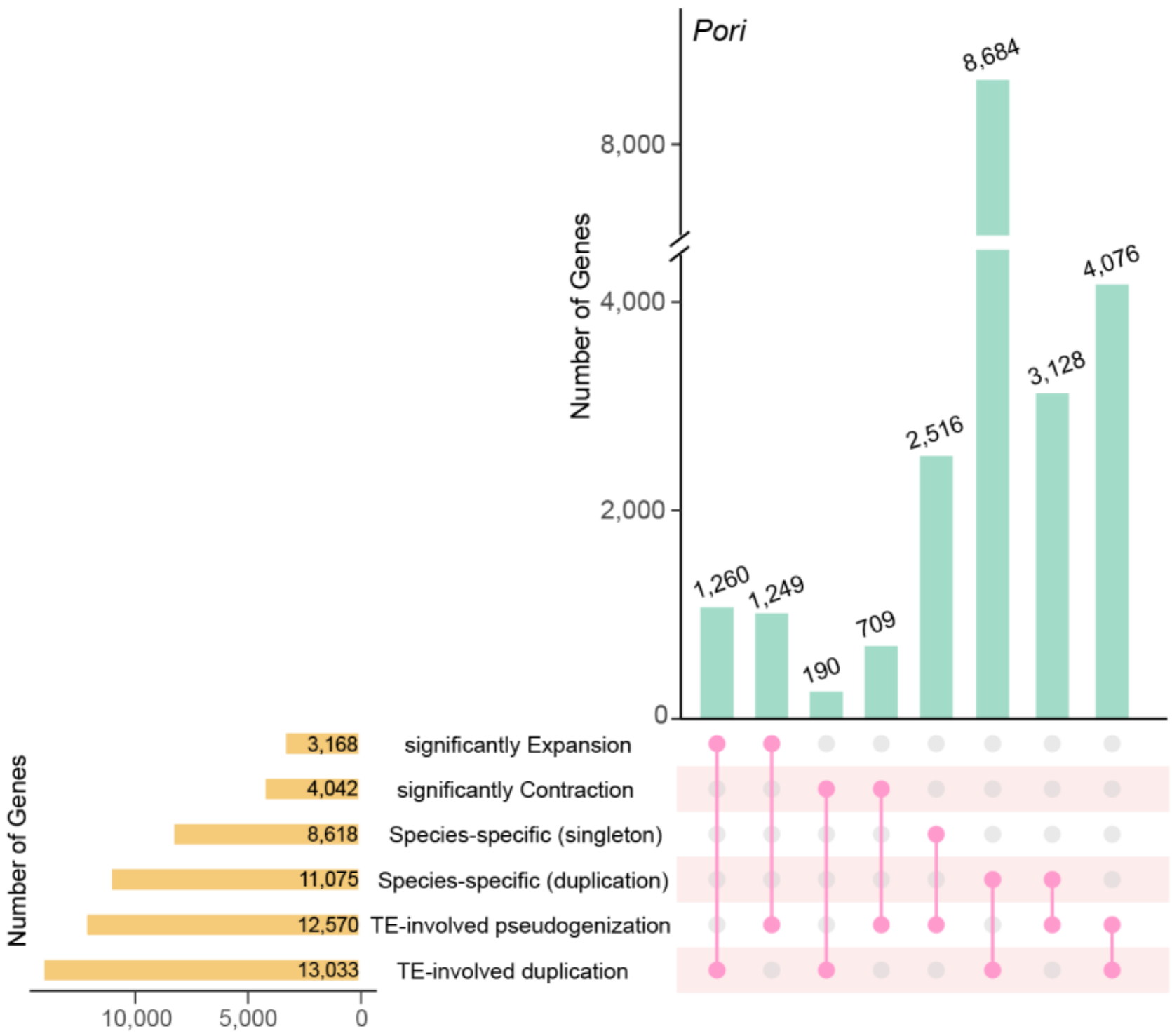

Supplemental Figure 28. Contributions of TE-mediated gene duplication and pseudogenization events to significant expansion, contraction, and species-specific dynamics of gene families in *P. orientalis*. UpSet plot illustrates the detailed intersection quantities for genes.

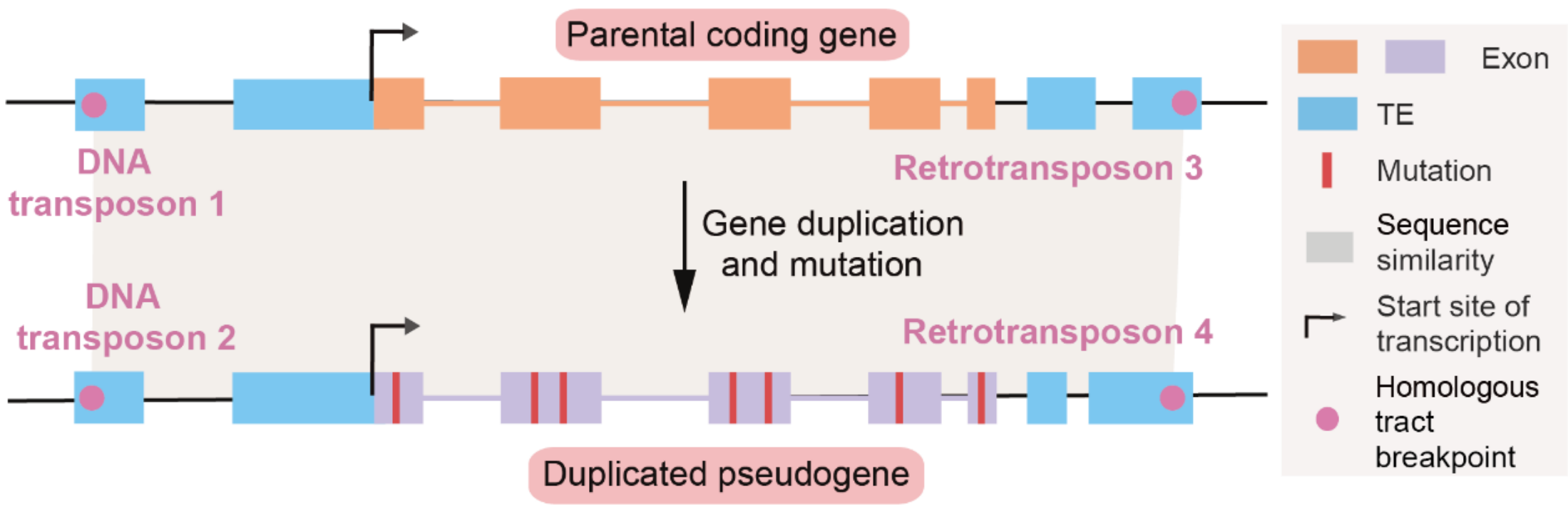

Supplemental Figure 29. Schematic representation of the identification of TE-mediated (TE-recombination) generation of duplicated pseudogenes.

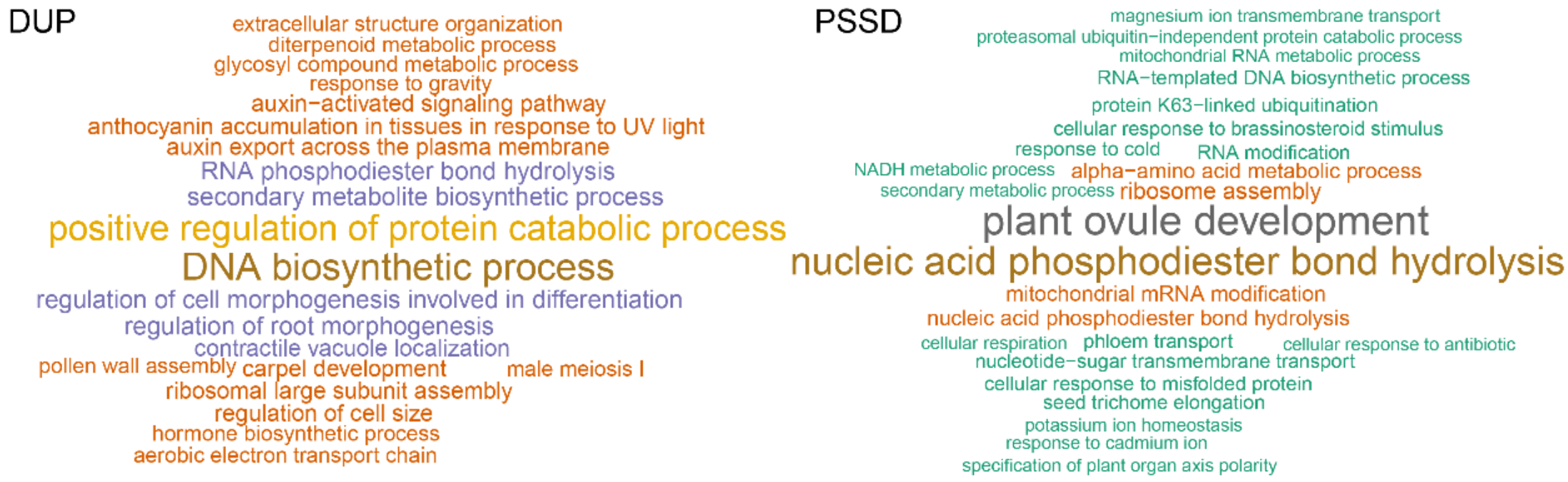

Supplemental Figure 30. GO enrichment analysis of source genes of TE-mediated pseudogenization in 12 gymnosperm species. GO terms with an adjusted *p*-value < 0.01 are plotted. The sizes of the GO terms in the word cloud correlate with their statistical significance. DUP: duplicated pseudogene, PSSD: processed pseudogene.

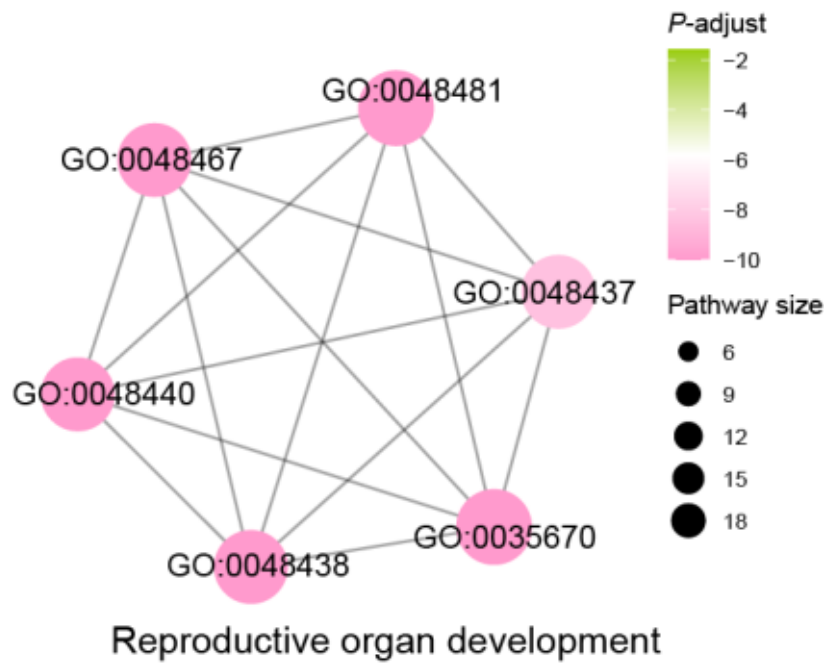

Supplemental Figure 31. Network and clustering of GO terms derived from source genes of transcribed pseudogenes under positive selection, identified through enrichment analysis in *P. orientalis*.

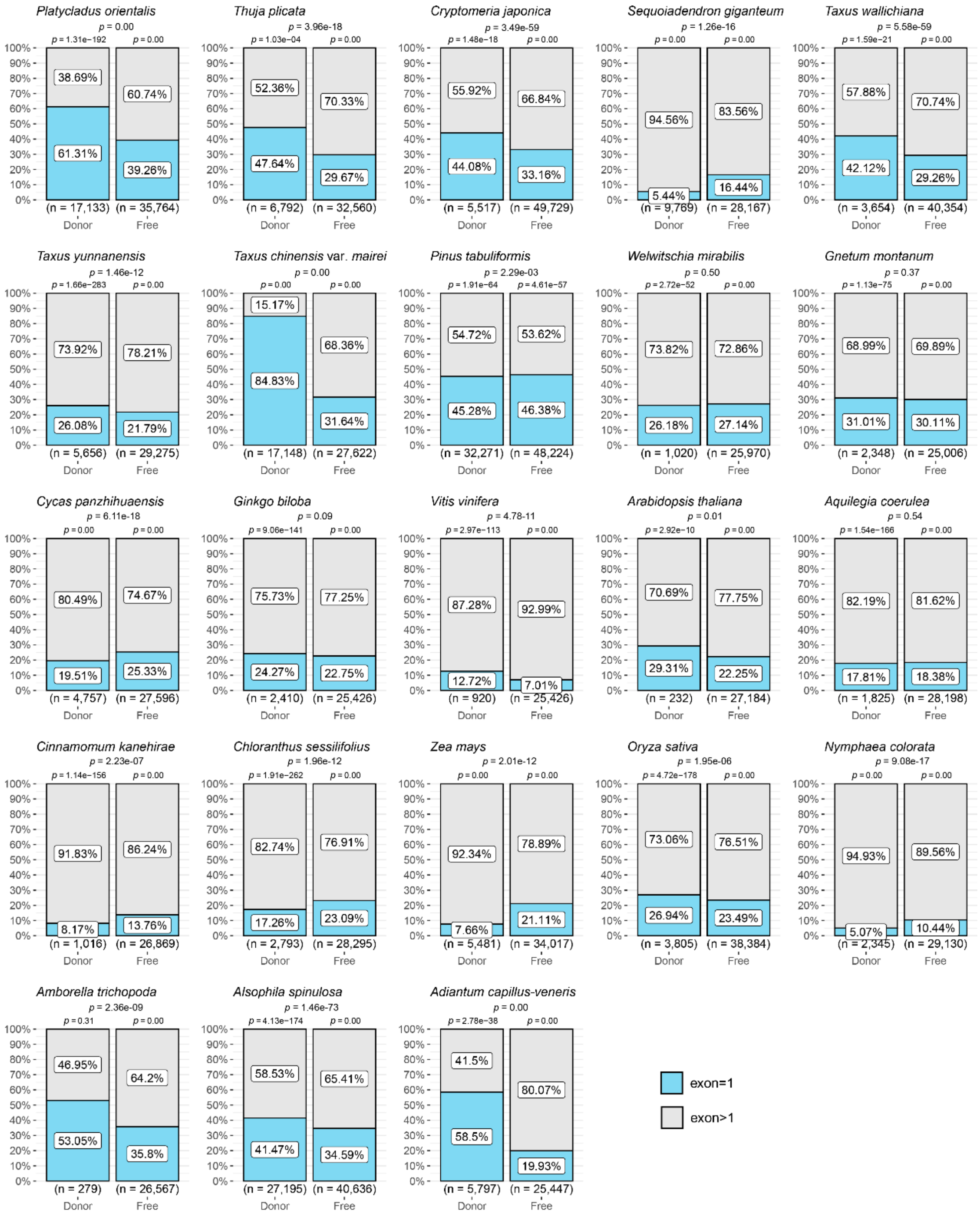

Supplemental Figure 32. Chi-square test analyses of the proportion of single-exon and multi-exon genes within donor and free genes across 23 species.

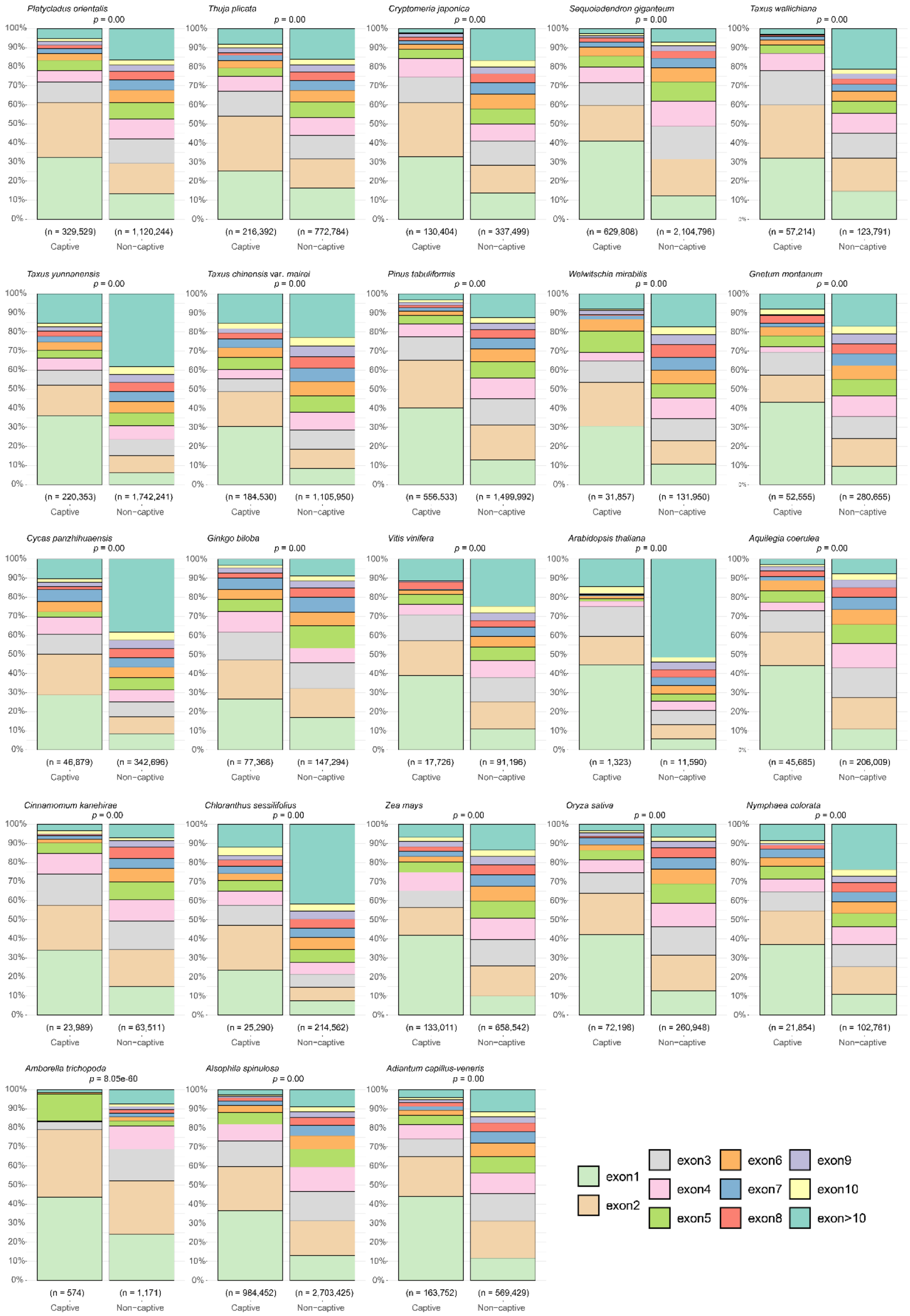

Supplemental Figure 33. Chi-square test analyses of the proportion of captive exon types to non-captive exon types within multi-exon donor genes across 23 species.

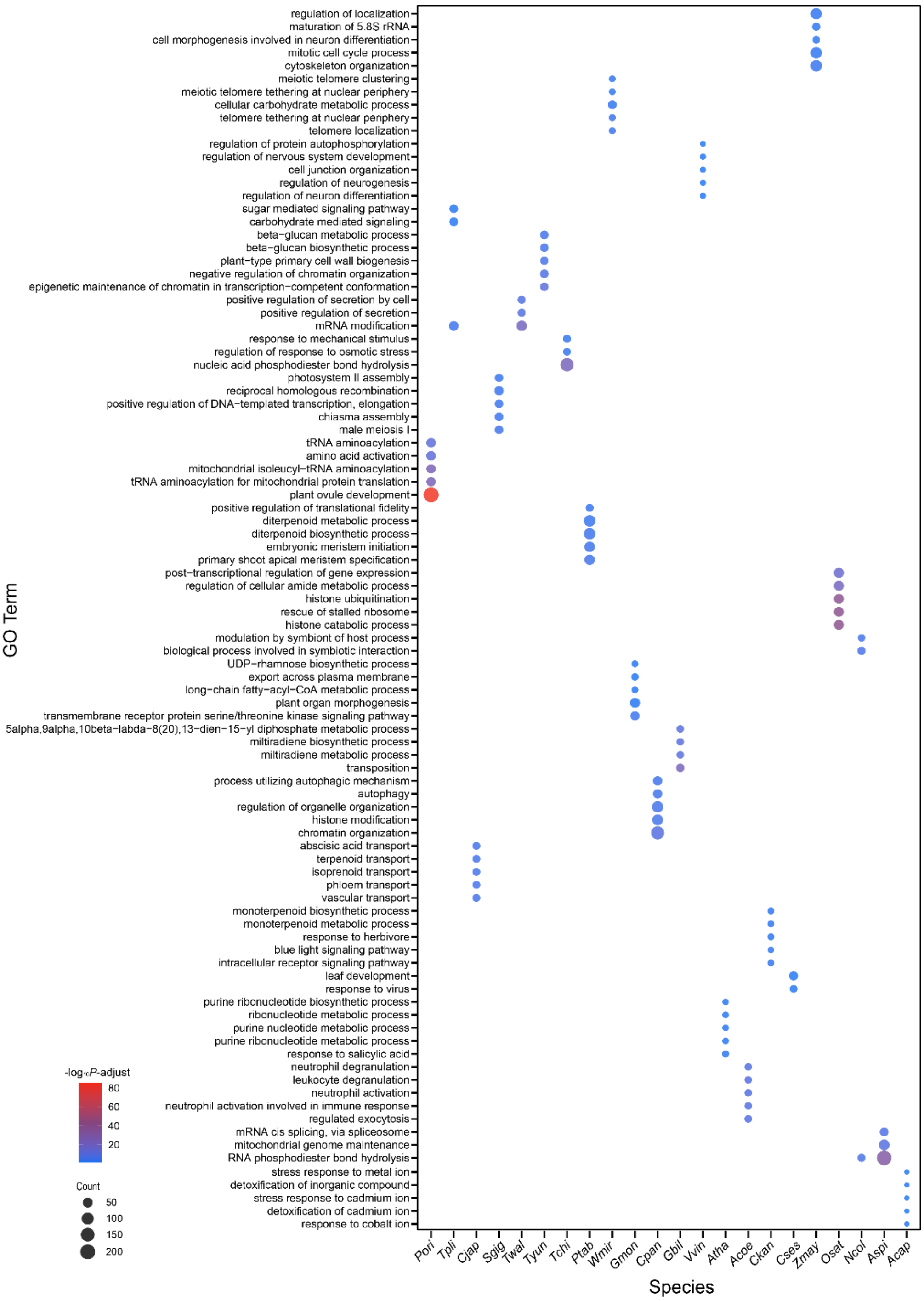

Supplemental Figure 34. Comparative GO enrichment analysis of genes with captured fragments across 23 species (adjusted *p*-value < 0.05). The top 5 functionally enriched GO terms for each species are shown. *Pori*: *Platycladus orientalis*; *Tpli*: *Thuja plicata*; *Cjap*: *Cryptomeria japonica*; *Sgig*: *Sequoiadendron giganteum*; *Twal*: *Taxus wallichiana*; *Tyun*: *Taxus yunnanensis*; *Tchi*: *Taxus chinensis* var. *mairei*; *Ptab*: *Pinus tabuliformis*; *Wmir*: *Welwitschia mirabilis*; *Gmon*: *Gnetum montanum*; *Cpan*: *Cycas panzhihuaensis*; *Gbil*: *Ginkgo biloba. Vvin*: *Vitis vinifera*; *Atha*: *Arabidopsis thaliana*; *Acoe*: *Aquilegia coerulea*; *Ckan*: *Cinnamomum kanehirae*; *Cses*: *Chloranthus sessilifolius*; *Zmays*: *Zea mays*; *Osat*: *Oryza sativa*; *Ncol*: *Nymphaea colorata*; *Aspi*: *Alsophila spinulosa*; *Acap*: *Adiantum capillus-veneris.*

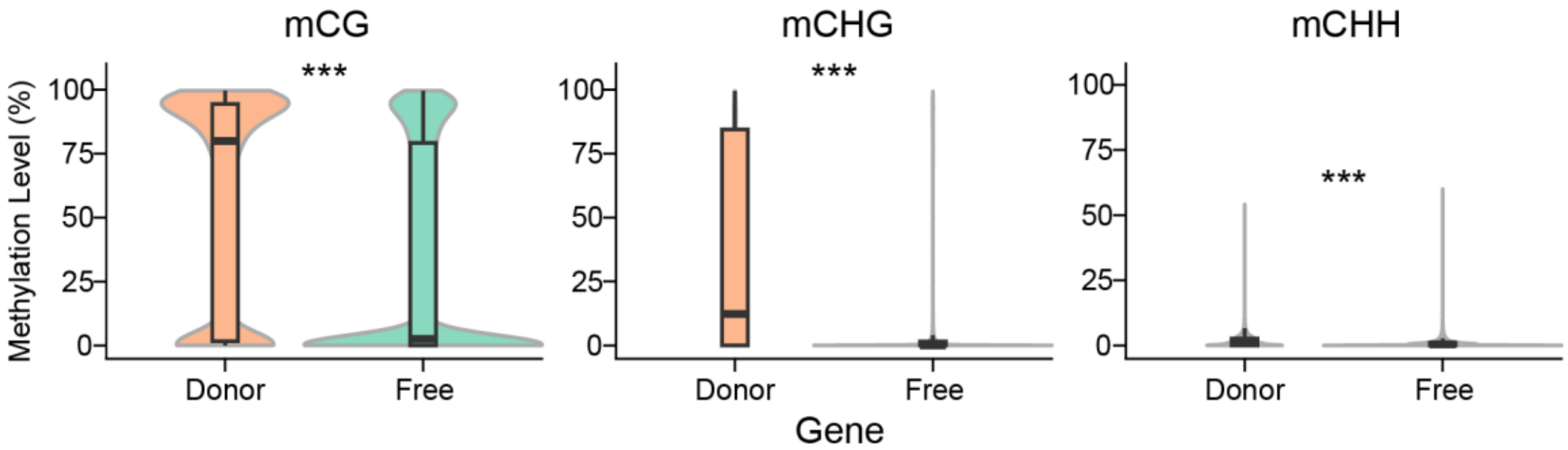

Supplemental Figure 35. Distribution of mCG, mCHG, and mCHH methylation levels in the exonic regions of the donor and free genes. Mann-Whitney U test was employed to assess the statistical significance, *** $p < 0.001$.

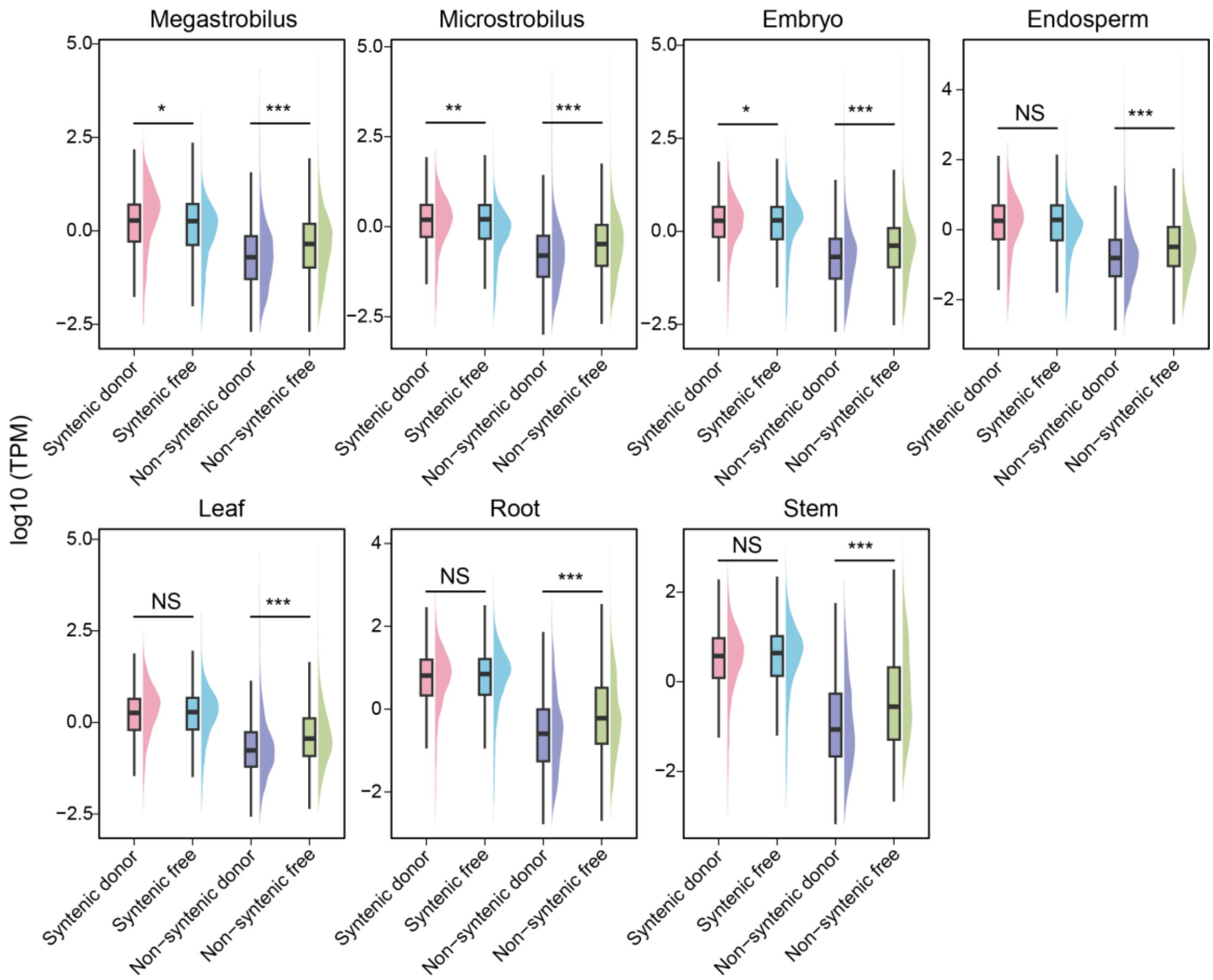

Supplemental Figure 36. Comparison of expression levels of four gene types in seven tissues. Mann-Whitney U test: ***, $p < 0.001$; NS, no significant difference.

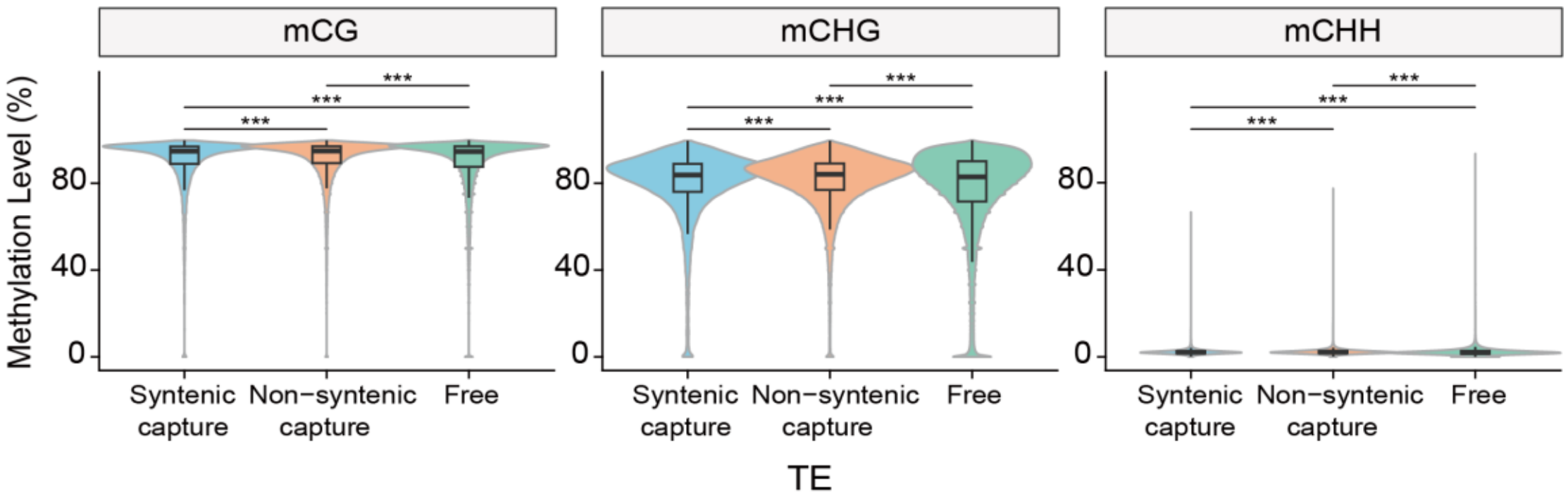

Supplemental Figure 37. Distribution of mCG, mCHG, and mCHH methylation levels in three types of TEs. Mann–Whitney Wilcoxon test: ∗∗∗, $p < 0.001$.

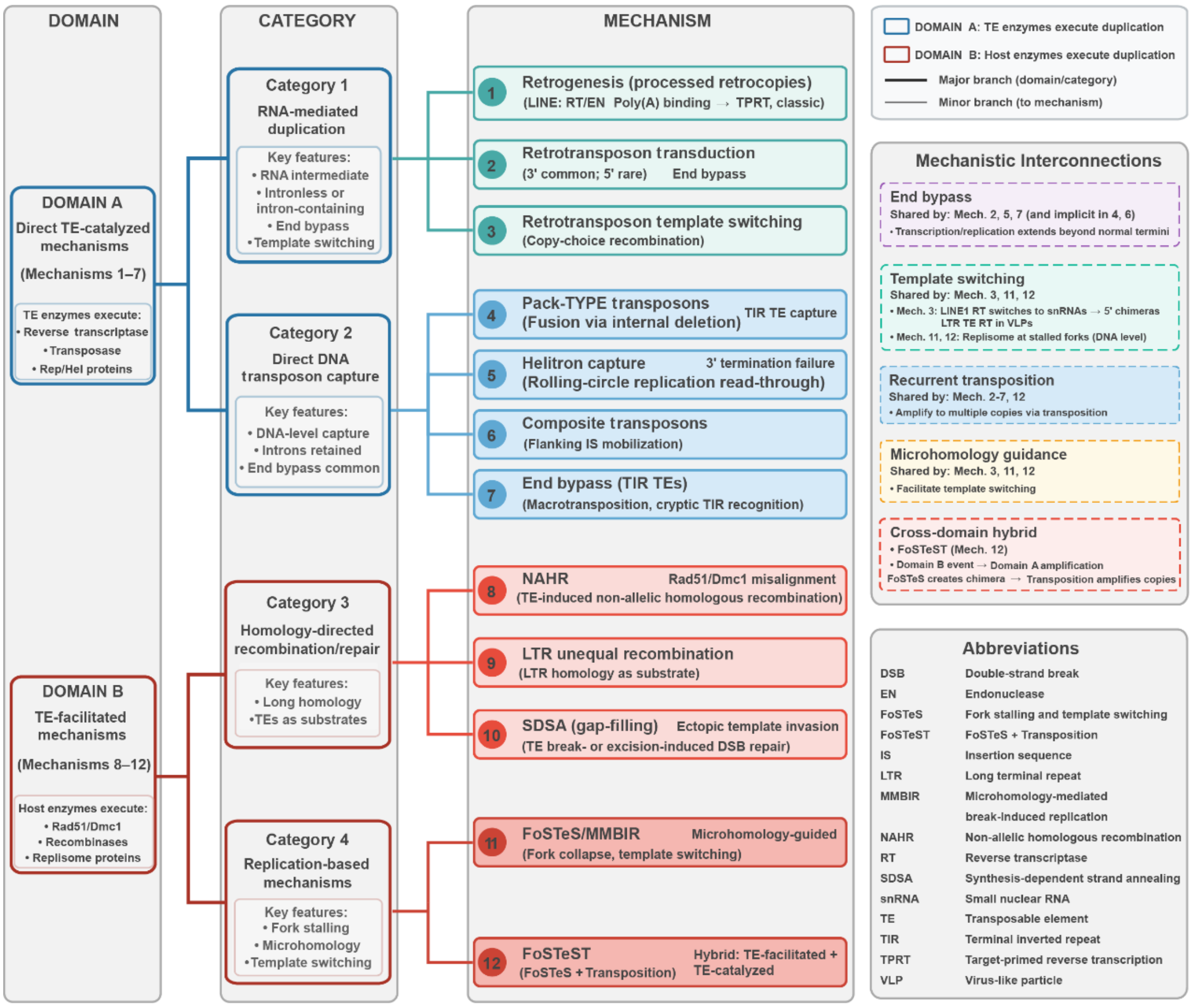

Supplemental Figure 38. Hierarchical classification of 12 TE-mediated gene duplication mechanisms. The classification scheme is organized into two primary domains, four intermediate categories, and 12 terminal mechanisms. Domain A encompasses mechanisms wherein TE-encoded enzymes directly catalyze the duplication process, whereas Domain B comprises mechanisms where TEs function as substrates or triggers for host cellular machinery. Each domain bifurcates according to the underlying molecular process: RNA-mediated duplication and DNA transposon capture under Domain A; homology-directed recombination and replication-associated errors under Domain B. Terminal labels denote the core molecular triggers or pathways characterizing 12 mechanisms, thereby illuminating the mechanistic logic of the classification. The top-right panel highlights functional interconnections and overlaps among distinct mechanisms.

Supplemental Notes

Supplemental Note 1. Genome sequencing
Supplemental Note 2. Genome survey and assembly
Supplemental Note 3. Gene annotation
Supplemental Note 4. Genome evolution and chromosome homology
Supplemental Note 5. Molecular mechanisms of TE-mediated gene duplication

Supplemental Note 1. Genome sequencing

ONT, PacBio SMRT and Illumina whole genome sequencing

To obtain an assembly with both high precision and good continuity, we combined techniques of both long- and short-read sequencing, encompassing Oxford Nanopore Technologies (ONT), PacBio Single-Molecule Real-Time (SMRT), Illumina, and Hi-C sequencing. Fresh leaves of *P. orientalis* were harvested from one individual plant for four distinct sequencing techniques.

For ONT sequencing, total DNA was extracted by using the cetyltrimethylammonium bromide (CTAB) technique along with a Genomic DNA Kit (Qiagen, Hilden, Germany). After purification and quantification, adaptors were tied to extracted DNA, utilizing the LSK109 kit (Oxford Nanopore Technologies). Consequently, sequencing libraries with DNA inserts of approximately 20 kb were structured and sequenced on a GridION X5 instrument.

For PacBio SMRT sequencing technique, genomic DNA, which had been sheared and concentrated, underwent size selection via the Blue Pippin system (Sage Sciences). Subsequently, sequencing libraries were established with DNA inserts of 20 kb, in accordance with the amplicon library protocol delineated by PacBio. Sequencing was carried out on a PacBio Sequel II platform, employing P6-C4 chemistry (3 SMRT cells).

For the Illumina sequencing, total DNA was extracted with the DNeasy Plant Mini Kit (QIAGEN, Inc.), followed by DNA purification completed using the Mobio PowerClean Pro DNA Clean-Up Kit (MO BIO Laboratories, Inc.). Paired-end (PE) libraries, of 150 bp, were devised deploying the DNAseq Library Index Kit and the sequencing was conducted on an Illumina HiSeq platform. Short reads were processed using fastp v0.19.3 (Chen et al., 2018) to exclude adapter sequences, leading and trailing bases with a quality score less than 20, along with reads maintaining an average per-base-quality of 20 via a 4-bp sliding window.

Approximately 40.27 million long reads were produced via ONT, resulting in 808.87 Gb (~100× coverage of the assembled genome) demonstrating an average read length of 20.09 kb, N50 of 25.47 kb, and maximum read length of 1,253.43 kb (**Supplemental Table 1**). A total of 21.33 million PacBio long reads were generated, yielding 256.02 Gb (roughly 30×) with an average read length of 12.00 kb (**Supplemental Table 1**). Post quality control, we acquired approximately 1,500 million clean short-read reads from Illumina (~475.52 Gb and around 57×) (**Supplemental Table 1**).

Hi-C sequencing

In the construction of the Hi-C library, we adhered to a prototypical methodology as delineated (Duan et al., 2010). Following the fixation of the leaf tissue with formaldehyde and subsequent lysis, the resultant cross-linked chromatin was subjected to enzymatic digestion utilizing the MboI restriction endonuclease. The cleaved DNA fragments were then subjected to biotinylation at the 5' protruding termini. Ligation of these blunt-end fragments facilitated the formation of chimeric junctions, which underwent subsequent purification, mechanical fragmentation, and enrichment for biotin-marked segments. These biotin-labeled DNA fragments were then processed through end-repair, adapter attachment, and polymerase chain reaction amplification to construct paired-end sequencing libraries. The assembled libraries were subsequently sequenced on an Illumina HiSeq platform.

In Hi-C sequencing, we obtained around 919.33 Gb of 6228.89 million reads,

approximately 110× coverage (**Supplemental Table 1**).

ONT and Illunima RNA sequencing

For the purpose of gene annotation, ONT full-length transcriptome sequencing was employed on needle leaf tissue. Total RNAs were extracted utilizing the RNAprep Pure Plant Kit (TIANGEN Biotech, China). The integrity of the isolated RNA was assessed via an Agilent 2100 Bioanalyzer (Agilent, USA). First-strand cDNA synthesis was performed on the purified total RNA from each sample employing the SuperScript IV First-Strand Synthesis System (Invitrogen, USA), followed by PCR amplification using the LongAmp Taq (NEB, USA) to generate double-stranded cDNA. Subsequent steps included FFPE DNA repair, end repair (NEB, USA), and adaptor ligation utilizing T4 DNA ligase (NEB, USA) on the PCR amplified products. DNA purification was then achieved with Agencourt XP beads, as per the ONT protocol. Ultimately, the constructed cDNA libraries were sequenced on the PromethION platform. In summary, we obtained approximately 14.84 Mb clean reads, roughly equivalent to 17.42 Gb **(Supplemental Table 1)**.

Furthermore, Illunima RNA sequencing was utilized on five distinct tissue types (namely, embryos, megagametophytes, leaves, megastrobili, and microstrobili) for the purpose of expression of genome elements with each stage represented by three biological replicates. The isolation of mRNA was accomplished via the NEBNext Poly(A) mRNA Magnetic Isolation Module. Strand-specific libraries were constructed using the NEBNext Ultra RNA Library Prep Kit, and sequencing was carried out on an Illumina HiSeq machine operating in 150 bp PE mode.

ATAC sequencing and WGBS

For assay for transposase accessible chromatin sequencing (ATAC-seq), following nuclear extraction, nuclei were resuspended in a translocation reaction mixture comprising Tn5 transposase and incubated with purified DNA, subsequently undergoing Polymerase Chain Reaction (PCR) amplification in accordance with the established PCR protocol. The methodology for nuclear extraction and incubation conformed to the protocols delineated by the previous study (Guo et al., 2023). The library was sequenced on the Illumina sequencing platform.

gDNA and unmethylated lambda DNA were combined and adjusted to 80 mL with 1x TE, then fragmented to 300 bp. Blunt ends were generated by filling single-stranded overhangs using T4 DNA polymerase and Klenow fragment. Fragments were dA-tailed and phosphorylated to enable ligation to adaptors. 300-600 bp fragments were selected by gel electrophoresis and recovered magnetically. Bisulfite conversion involved treating DNA with bisulfite, converting unmethylated cytosines to uracils while leaving methylated cytosines unchanged. KAPA HiFi DNA Polymerase amplified uracil-containing DNA. Library quantitation for whole genome bisulfite sequencing (WGBS) utilized Qubit 3.0 Fluorometry. Agilent 2100 Bioanalyzer confirmed insert size. StepOnePlus Real-Time PCR checked library molality (>10mM). WGBS libraries were sequenced as 150bp paired-end reads on Illumina NovaSeq 6000 with S4 flow cell.

Supplemental Note 2. Genome survey and assembly

Genome survey

To ascertain the genome size, level of heterozygosity, and repeat content of *P. orientalis*, we implemented the *k*-mer frequency analytical framework. Raw Illumina DNA sequencing reads underwent quality filtering via fastp v0.19.3 (Chen *et al.*, 2018). Subsequently, 468.36 Gb clean reads were employ yed to compute the *k*-mer frequency, with *k* set at 17, utilizing Jellyfish v.2.2.8 (Marçais and Kingsford, 2011) and GCE v1.0.2 (https://github.com/fanagislab/GCE). Our estimations yielded a genome size of 8.22–8.47 Gb, accompanied by a peak depth of 49 for the 17-mer **(Supplemental Figure 2)**. The levels of heterozygosity and repeat content were determined to be 0.81% and 86.11%, respectively.

***De novo* genome assembly**

The *de novo* genome assembly was conducted in a three-step procedure: primary assembly with the Overlap-Layout-Consensus method; Hi-C scaffolding; and polishing **(Supplemental Figure 1)**. First, the raw ONT long reads were corrected by NextDenovo v2.3.0 (https://github.com/Nextomics/NextDenovo) and Canu v1.7 (Koren et al., 2017). After evaluating the continuity and completeness of the different primary assemblies, an initial, assembly v0.1, computed from NextDenovo and SmartDenovo (Liu et al., 2021a) was selected as the optimal assembly for further polishing due to its longest contig size and high L90 index. This initial assembly v0.1 was further improved with three rounds of polishing using the Arrow algorithm (Chin et al., 2013) based on PacBio reads. High quality Illumina short reads were then aligned to the initial assembly, and NextPolish (v1.0.0) (Hu et al., 2020) was used to enhance single-base accuracy in the contigs in four rounds to produce assembly v0.2.

Subsequently, valid Hi-C data was processed together with initial assembly v0.2 using the 3D-DNA pipeline v180922 (https://github.com/theaidenlab/3d-dna) to generate primary scaffolds. These scaffolds were roughly split by Juicebox v1.8 (https://github.com/aidenlab/Juicebox), and each scaffold was processed with 3D-DNA. The new scaffolds were then meticulously optimized by removing errors in insertions, junctions, order, and mis-joins. After this scaffold adjustment, chromosome-level scaffolds and scattered contigs were merged to produce assembly v0.3 for further gap closing and polishing. Gaps were closed using LR_Gapcloser v1.1 (https://github.com/CAFS-bioinformatics/LR_Gapcloser) with corrected ONT long reads and four rounds of pilon polishing with filtered Illumina short reads to produce assembly v0.4.

For the polishing, we recognized and selectively removed substandard contigs, such as redundant contigs (i.e. same region in homologous chromosomes) with Redundans v0.13c (Pryszcz and Gabaldon, 2016), and contigs representing either low coverage below 10-fold or high non-coverage above 60%. Thereafter, the filtered assembly was aligned to the NT database with blastn v2.2.28+ (Boratyn et al., 2012) (coverage of 90%) to identify any potential pollution (external DNA sequences) presented in the assembly, and we confirmed that the final assembly v1.0 was not polluted by cross-contamination (**Supplemental Table 2**).

The final chromosome-level assembly had a total size of 8.39 Gb, exhibiting greater contiguity than any published gymnosperm genomes thus far. Specifically, the assembly contained 384 contigs (contig N50 = 57.54 Mb; longest contig = 229.03 Mb), and 126 scaffolds (scaffold N50 = 739.02 Mb; longest scaffold = 960.93 Mb). Eleven pseudochromosomes were

assembled, covering approximately 99.71% of the total assembled genome **(Supplemental Table 3)**.

The genome demonstrated superior quality, as confirmed by key assembly metrics and multiple assessments. Firstly, compleasm (a faster and more accurate reimplementation of BUSCO) analysis showed that 92.0% of the 1614 core eukaryotic genes were present in the assembly, demonstrating the high completeness of the assembled genome **(Supplemental Figure 3 and Supplemental Table 4)**. Moreover, the high fidelity of the assembly was supported by two high mapping rates of 99.54% (ONT) and 99.63% (Illumina), two minimum 20-fold genome coverages of 99.73% (ONT) and 96.44% (Illumina), and single base error rate of ~0.0005% after mapping Illumina reads to the final assembly **(Supplemental Table 5)**. Additionally, we observed significant collinear relationships between the assembled chromosomes and the previous genetic map (Jin et al., 2019) **(Supplemental Figure 4B)**, providing additional support for the high-quality genome assembly.

Supplemental Note 3. Gene annotation

Gene structural annotation

For the purpose of protein homology evidence in coding gene structural annotation, a total of 146,741 non-redundant protein sequences from related species, including *Ginkgo biloba* (Guan et al., 2016), *Gnetum montanum* (Wan et al., 2018), *Pseudotsuga menziesii* (Neale et al., 2017), *Pinus lambertiana* (Stevens et al., 2016) were gathered. Regarding transcriptome-based evidence, three strategies were employed to assemble Illumina transcriptome sequencing reads from NCBI (ERR2040822, SRR3410807, SRR3410808, SRR3410809, SRR3410810, SRR3410811, SRR3410812, SRR5892426, SRR7415721, and SRR7415720) into transcripts: 1) *de novo* assembly was conducted using Trinity v2.13.2 (Grabherr et al., 2011); 2) a genome-guided assembly was performed using HISAT2 v2.2.1 (Kim et al., 2015) and Trinity v2.13.2; 3) another genome-guided assembly was conducted using HISAT2 v2.2.1 and StringTie v2.2.0 (Pertea et al., 2015). ONT full-length transcriptome sequencing reads were mapped to the reference using Minimap2 v2.17 (Li, 2018), and subsequent clustering was performed using the spliced_bam2gff and cluster_gff commands. In addition, incorporating the transcripts from the study (Hu et al., 2016), all aforementioned transcriptomes were merged, eliminating redundancy with CD-HIT v4.8.1 (Fu et al., 2012) (95% identity and 95% coverage), resulting in a total of 414,319 transcript sequences.

The gene structure was annotated using the PASA v2.5.0 pipeline (Haas et al., 2003) firstly, basing on transcriptional evidence. High-quality open reading frame (ORF) structures generated by PASA v2.5.0 were used to train the parameter model of *ab initio* via AUGUSTUS v3.4.0 (Stanke et al., 2008).

Secondly, a library of repetitive sequences was generated *de novo* using RepeatModeler v1 (http://www.repeatmasker.org). This library was then employed to annotate and mask repetitive elements within the genome via RepeatMasker v4.0.5 (http://www.repeatmasker.org), facilitating gene prediction in the MAKER2 workflow (Holt and Yandell, 2011) **(Supplemental Table 13)**. The primary workflow was as follows. A gene model was developed for *ab initio* gene structure prediction using trained parameter model with AUGUSTUS v3.4.0. Transcript and homologous protein evidence were aligned to the masked genome using BLAST+ v2.11.0

(Camacho et al., 2009) and optimized with exonerate v2.4.0 (Slater and Birney, 2005). Subsequently, AUGUSTUS integrated models derived from three gene predictions, further enriched with UTR annotations based on transcriptomic evidence. Genes exhibiting anomalies in their coding frames, such as internal stop codons, ambiguous bases, absence of start or stop codons, or those of insufficient length (<= 50 amino acids), were excluded, culminating in gene annotations defined by the MAKER2 pipeline.

Thirdly, the BUSCO v.4.1.2 pipeline (Simão et al., 2015) was also executed, generating annotation files. To further improve the precision of annotation, we integrated and refined the coding gene predictions from the PASA, MAKER2, and BUSCO pipelines using the application of EVidenceModeler (EVM) v1.1.1 (Haas et al., 2008). Subsequently, transcripts and associated genes exhibiting TE domain coverage exceeding 30% (using TEsorter with parameters: -cov 30 -eval 1e-10) (Zhang et al., 2022) and genes with excessively short annotations (≤50 amino acids) were filtered out, ultimately yielding a reliably annotated gene set. Coding gene annotations of gymnosperms, which exclusively retained the longest transcript isoforms, were assessed for completeness using BUSCO v.4.1.2 with embryophyta_odb10 in protein mode and OMArk v.0.3.0 (Nevers et al., 2024) webserver (https://omark.omabrowser.org/, OMAmer database v2.0.0) with the ancestral clade spermatophyta.

The identification of non-coding RNAs (ncRNAs) such as transfer RNAs (tRNAs), ribosomal RNAs (rRNAs), and other ncRNAs was performed using tRNAscan-SE v2.0 (Grotewold, 2006), barrnap (https://github.com/tseemann/barrnap), and RfamScan v9.1 (http://eggnogdb.embl.de/), respectively. Finally, 52,897 protein-coding genes, 3,367 tRNAs, 547 rRNAs, and additional 684 ncRNAs were identified. The average lengths of total gene, transcript, coding sequence (CDS), exon, and intron sequences were 19,881.6, 1,890.1, 1,005.5, 307.9, and 7,792.3 bp, respectively **(Supplemental Table 9)**.

Gene functional annotation

Three strategies were employed to perform functional annotation of protein-coding genes in this study **(Supplemental Table 11)**. Firstly, we utilized the eggNOG-mapper (Huerta-Cepas et al., 2017) with (--target_taxa Viridiplantae) to map gene sequences. Then to annotate predicted gene models, a comparison was made with proteins in four protein databases: (i) NR (https://www.ncbi.nlm.nih.gov/), (ii) Swiss-Prot protein database (Albalat and Canestro, 2016), (iii) Translated EMBL-Bank (as part of the International Nucleotide Sequence Database Collaboration TrEMBL) (Albalat and Canestro, 2016), (iv) *Arabidopsis thaliana* genes (Lamesch et al., 2012) using BLASTP with a minimum identity of 30% and an E-value threshold of 1e-05. For domain similarity predictions, the predicted protein sequences were annotated using InterProScan (version 5.27-66.0) (http://www.ebi.ac.uk/InterProScan) with default parameters. The annotations derived from all strategies were concatenated to obtain the final gene functional annotations.

Supplemental Note 4. Genome evolution and chromosome homology

Genome family and phylogenomic analysis

We selected 27 representative species **(Supplemental Table 16)** of angiosperms (10,

basalmost branches, monocots, and eudicots), gymnosperms (15, 5 distinct clades: cycads, Ginkgo, gnetophytes, conifer I, and conifer II), and pteridophytes (2) to construct orthogroups using OrthoFinder v2.3.1 (Emms and Kelly, 2019). The longest transcript isoform for each locus was selected for putative gene family identification. A total of 965,468 genes, representing 91.35% of the total sequences, derived from the 27 selected species were categorized into 54,368 gene families. Among these, 30,547 families were universally shared across all genomes, while 23,821 families were unique to specific species **(Supplemental Table 15)**.

The phylogenetic tree (species tree) was reconstructed using 692 low-copy gene families. Each family included in the analysis satisfied the following criterion: a single-copy ortholog was present in at least 80% of the species, with the remaining species either lacking the gene or retaining it as a low-copy ortholog. For gene families with low-copy orthologs in some species, the copy exhibiting the shortest branch length was chosen as the representative sequence for tree construction. This approach prioritizes slowly evolving orthologs, which are typically under strong purifying selection, thereby mitigating phylogenetic artifacts such as long-branch attraction (LBA) and enhancing the accuracy of the species tree inference. Nucleotide sequences from 692 single-copy orthologs were aligned using MUSCLE v3.8.3.1 (Edgar, 2004) under default parameters. The alignment was subsequently concatenated and subjected to trimming using trimAl v1.2 with the -automated1 option (Capella-Gutiérrez et al., 2009). A maximum likelihood phylogenetic species tree was then constructed using IQ-TREE v1.6.7 (-m MF) (Nguyen et al., 2015), employing 1000 bootstrap replicates and the optimal model of JTT+F+R6 as specified input settings.

Concatenation-based methodologies were employed to infer species tree topologies, yielding robust bootstrap values. Divergence times between individual species were estimated using the MCMCTree module within PAML (Yang, 2007) on the basis of the 4-fold degenerate (4d) sites, the whole coding sequences, and the species tree. The 4d sites were extracted from the gene sequences that were retained in the preceding step. These divergence times were calibrated utilizing known calibration points from the Timetree database (http://timetree.org/) by imposing soft bounds at the split nodes of *Arabidopsis thaliana*–*Vitis vinifera* (109.8–124.4 mya), *Zea mays*–*Oryza sativa* (41.4–51.9 mya), Ginkgo–Cycas (275.3–316.4 mya), and *Welwitschia mirabilis*–*Gnetum montanum* (94.2–132.0 mya). Utilizing CAFÉ v5.0 (Mendes et al., 2021), we assessed the expansion and contraction of gene families between extant species and their most recent common ancestors (MRCAs) through MRCA analysis. The visualization of the tree was accomplished using tvBOT (Xie et al., 2023).

The high consistency between 4d sites and whole coding sequence analyses (Spearman $R = 0.98$, $p < 0.001$) validates the robustness of temporal estimates. Phylogenetic and molecular dating indicated that *P. orientalis* was most related to *Thuja plicata*, with an estimated divergence time of approximately 80.02 million years ago (mya), belonging to the conifer II clade. The split between conifer I and conifer II was estimated at around 295.17 mya. The divergence time estimates obtained from the 4d sites and the whole coding sequences were highly consistent. In total, 887 gene families were found expanded in *P. orientalis*, with 155 (containing 3,404 genes) significantly expanded ($p < 0.05$). Meanwhile, 1,173 gene families were contracted, with 370 (containing 4,074 genes) significantly contracted ($p < 0.05$), relative to the common ancestor of *P. orientalis* and *T. plicata* **(Figure 2A and Supplemental Table 17)**. The expanded gene families show significant functional enrichment for pathways related to

plant growth and development (e.g. plant ovule development, regulation of plant organ formation), biosynthesis and metabolism (e.g. lignin, monoterpene and steroid biosynthetic processes), and immunity and defense responses (e.g. cellular response to UV, defense response to insects and bacteria, resistance-mediated immunity), concurrently, certain gene families associated with plant organ senescence, auxin metabolic processes, and other pathways exhibited contraction **(Supplemental Figure 6 and Supplemental Tables 18, 19)**. Unassigned single-copy genes that could not be clustered with orthologous genes from other species (single gene) and species genes comprising over 98% of the total genes in an orthogroup (duplicated gene) were collectively considered as species-specific genes. In *P. orientalis*, there were 8,634 single genes constituting 16.3% of total genes, as well as 680 orthogroups encompassing 11,081 duplicated genes, amounting to 19,715 species-specific genes in total, which were particularly enriched in developmental processes associated with female gametophytes of gymnosperms, lipid transport **(Supplemental Figure 6 and Supplemental Table 20)**.

WGD and genomic synteny

To evaluate potential whole-genome duplication (WGD) event in *P. orientalis*, we analyzed the distribution of synonymous substitutions per site (*K*s) for each paralog. All species protein sequences were aligned all-versus-all using DIAMOND v2.0.2.140 (Buchfink et al., 2015) (blastp -e 1e-5 --max-target-seqs 10), respectively. Syntenic regions with collinear paralog pairs were identified by MCScanX (Wang et al., 2012) (https://github.com/wyp1125/MCScanX) with default parameters. ParaAT v2.0 (Zhang et al., 2012) constructed multiple protein-coding DNA alignments with parameters (-m muscle -f axt), and KaKs_Calculator v2.0 (Wang et al., 2010) calculated *K*s values for each paralog pair with default parameters (-m YN). The WGDI (Sun et al., 2022) software was also employed to sequentially execute the -icl, -ks, -bi, -c, and -bk parameters to identify syntenic blocks and syntenic genes. Tree2GD v1.0.43 (Chen et al., 2022) with default parameters was utilized to confirm the occurrence of the WGD event in gymnosperms. A gene duplication was counted at a node if it included two paralogous clades shared by two or more species with high bootstrap support. Duplication events were mapped to the corresponding nodes on the species tree. To assess potential WGDs in gymnosperms, we estimated the number of gene duplications (GDs) across different retention types. The (AB)(AB) type was defined as gene phylogenies where both taxa A and B retained two gene copies following a putative shared WGD event, providing evidence for WGDs (Ren et al., 2018). We also recognized that loss (or non-detection) of one paralog in one lineage would result in (AB)A or (AB)B types in gene trees.

Syntenic gene pairs between *P. orientalis* and ten gymnosperm species (*Cryptomeria japonica* (Fujino et al., 2024), *Sequoiadendron giganteum* (Scott et al., 2020), *Taxus wallichiana* (Cheng et al., 2021), *Taxus yunnanensis* (Song et al., 2021), *Taxus chinensis* var. *mairei* (Xiong et al., 2021), *Pinus tabuliformis* (Niu et al., 2022), *Welwitschia mirabilis* (Wan et al., 2021), *Gnetum montanum* (Wan *et al.*, 2021), *Cycas panzhihuaensis* (Liu et al., 2022), and *Ginkgo biloba* (Liu et al., 2021b)) were identified using JCVI v0.84, the Python version of MCScan (Tang et al., 2008) (https://github.com/tanghaibao/jcvi/wiki/MCscan-(Python-version)). Using coding sequences (CDS) and annotation files as inputs, syntenic blocks for each species pair were identified with 'jcvi.compara.catalog_ortholog'. Syntenic blocks were filtered with

'jcvi.compara.synteny_screen' using parameters: --minspan = 30 --simple. Synteny patterns were detected using 'jcvi.compara.synteny_depth --histogram'.

Synteny analysis between *P. orientalis* and 10 other recently assembled chromosome-level gymnosperm species revealed varying degrees of genome reorganization via chromosomal exchanges during gymnosperm evolution **(Supplemental Figure 10)**, and chromosomes show rearrangements, inversions, expansions, or contractions by crossed, converging, or spreading lines **(Figures 2C, D and Supplemental Figure 11)**. Overall, inter-family chromosomal rearrangements were more common than intra-family, reflecting broader evolutionary divergence. *P. orientalis*, *C. japonica*, and *S. giganteum* all belong to Cupressaceae and have higher chromosomal synteny with each other versus other gymnosperms due to their closer phylogenetic relationship. Specifically, six *P. orientalis* chromosomes (Chr1, Chr3, Chr5, Chr6, Chr7, and Chr10) demonstrated nearly one-to-one synteny with corresponding chromosomes in *C. japonica* (Chr3, Chr10, Chr4, Chr7, Chr1, and Chr8) and *S. giganteum* (Chr3, Chr2, Chr6, Chr1, Chr7, and Chr9) **(Figure 2C)**. Interestingly, *S. giganteum* Chr6 was much shorter than its syntenic counterparts in both species. The other 5 chromosomes in *P. orientalis* (Chr2, Chr4, Chr8, Chr9 and Chr11) underwent several rearrangements during or following Cupressaceae radiation. *P. orientalis* (with 11 chromosomes) shared 12,569, 11,115, 9,541, and 8,904 syntenic gene pairs with conifers with 12 chromosomes, i.e. *T. wallichiana*, *T. yunnanensis*, *T. chinensis* var. *mairei*, and *P. tabuliformis*, respectively **(Supplemental Table 22)**, in accordance with their phylogenetic relationships. Except for an almost one-to-one syntenic relationship between *P. orientalis* chromosome 11 and *P. tabuliformis*, each chromosome of *P. orientalis* corresponded to partial segments of 2, 3 or 4 chromosomes of the other four conifers **(Figure 2D and Supplemental Figure 11)**, exhibiting complex and diverse chromosomal rearrangements during the evolution of 11 and 12 chromosome numbers in conifers. Two species from Gnetophyta, *W. mirabilis* and *G. montanum,* showed underwent substantial genome rearrangements and limited syntenic regions with *P. orientalis* chromosomes.

References

Albalat, R., and Canestro, C. (2016). Evolution by gene loss. Nat. Rev. Genet. **17**:379-391. https://doi.org/10.1038/nrg.2016.39.

Boratyn, G.M., Schaffer, A.A., Agarwala, R., Altschul, S.F., Lipman, D.J., and Madden, T.L. (2012). Domain enhanced lookup time accelerated BLAST. Biol. Direct **7**:12. https://doi.org/10.1186/1745-6150-7-12.

Buchfink, B., Xie, C., and Huson, D.H. (2015). Fast and sensitive protein alignment using DIAMOND. Nat. Methods **12**:59-60. https://doi.org/10.1038/nmeth.3176.

Camacho, C., Coulouris, G., Avagyan, V., Ma, N., Papadopoulos, J., Bealer, K., and Madden, T.L. (2009). BLAST+: architecture and applications. BMC Bioinf. **10**:421. https://doi.org/10.1186/1471-2105-10-421.

Capella-Gutiérrez, S., Silla-Martínez, J.M., and Gabaldón, T. (2009). trimAl: a tool for automated alignment trimming in large-scale phylogenetic analyses. Bioinformatics **25**:1972-1973. https://doi.org/10.1093/bioinformatics/btp348.

Chen, D., Zhang, T., Chen, Y., Ma, H., and Qi, J. (2022). Tree2GD: a phylogenomic method to detect large-scale gene duplication events. Bioinformatics **38**:5317-5321. https://doi.org/10.1093/bioinformatics/btac669.

Chen, S., Zhou, Y., Chen, Y., and Gu, J. (2018). fastp: an ultra-fast all-in-one FASTQ preprocessor. Bioinformatics **34**:i884-i890. https://doi.org/10.1093/bioinformatics/bty560.

Cheng, J., Wang, X., Liu, X., Zhu, X., Li, Z., Chu, H., Wang, Q., Lou, Q., Cai, B., Yang, Y., et al. (2021). Chromosome-level genome of Himalayan yew provides insights into the origin and evolution of the paclitaxel biosynthetic pathway. Mol. Plant **14**:1199-1209. https://doi.org/10.1016/j.molp.2021.04.015.

Chin, C.S., Alexander, D.H., Marks, P., Klammer, A.A., Drake, J., Heiner, C., Clum, A., Copeland, A., Huddleston, J., Eichler, E.E., et al. (2013). Nonhybrid, finished microbial genome assemblies from long-read SMRT sequencing data. Nat. Methods **10**:563-569. https://doi.org/10.1038/nmeth.2474.

Duan, Z., Andronescu, M., Schutz, K., McIlwain, S., Kim, Y.J., Lee, C., Shendure, J., Fields, S., Blau, C.A., and Noble, W.S. (2010). A three-dimensional model of the yeast genome. Nature **465**:363-367. https://doi.org/10.1038/nature08973.

Edgar, R.C. (2004). MUSCLE: multiple sequence alignment with high accuracy and high throughput. Nucleic Acids Res. **32**:1792-1797. https://doi.org/10.1093/nar/gkh340.

Emms, D.M., and Kelly, S. (2019). OrthoFinder: phylogenetic orthology inference for comparative genomics. Genome Biol. **20**:238. https://doi.org/10.1186/s13059-019-1832-y.

Fu, L., Niu, B., Zhu, Z., Wu, S., and Li, W. (2012). CD-HIT: accelerated for clustering the next-generation sequencing data. Bioinformatics **28**:3150-3152. https://doi.org/10.1093/bioinformatics/bts565.

Fujino, T., Yamaguchi, K., Yokoyama, T.T., Hamanaka, T., Harazono, Y., Kamada, H., Kobayashi, W., Ujino-Ihara, T., Uchiyama, K., and Matsumoto, A. (2024). A chromosome-level genome assembly of a model conifer plant, the Japanese cedar, *Cryptomeria japonica* D. Don. BMC Genomics **25**:1039. https://doi.org/10.1186/s12864-024-10929-4.

Grabherr, M.G., Haas, B.J., Yassour, M., Levin, J.Z., Thompson, D.A., Amit, I., Adiconis, X., Fan, L., Raychowdhury, R., Zeng, Q., et al. (2011). Full-length transcriptome assembly from RNA-Seq data without a reference genome. Nat. Biotechnol. **29**:644-652. https://doi.org/10.1038/nbt.1883.

Grotewold, E. (2006). The genetics and biochemistry of floral pigments. Annu. Rev. Plant Biol. **57**:761-780. https://doi.org/10.1146/annurev.arplant.57.032905.105248.

Guan, R., Zhao, Y., Zhang, H., Fan, G., Liu, X., Zhou, W., Shi, C., Wang, J., Liu, W., Liang, X., et al. (2016). Draft genome of the living fossil *Ginkgo biloba*. GigaScience **5**:49. https://doi.org/10.1186/s13742-016-0154-1.

Guo, S., Ji, Y., Zheng, Y., Watkins, C.B., Ma, L., Wang, Q., Liang, H., Bai, C., Fu, A., Li, L., et al. (2023). Transcriptomic, metabolomic, and ATAC-seq analysis reveal the regulatory mechanism of senescence of post-harvest tomato fruit. Front. Plant Sci. **14**:1142913. https://doi.org/10.3389/fpls.2023.1142913.

Haas, B.J., Salzberg, S.L., Zhu, W., Pertea, M., Allen, J.E., Orvis, J., White, O., Buell, C.R., and Wortman, J.R. (2008). Automated eukaryotic gene structure annotation using EVidenceModeler and the Program to Assemble Spliced Alignments. Genome Biol. **9**:R7. https://doi.org/10.1186/gb-2008-9-1-r7.

Haas, B.J., Delcher, A.L., Mount, S.M., Wortman, J.R., Smith, R.K., Jr., Hannick, L.I., Maiti, R., Ronning, C.M., Rusch, D.B., Town, C.D., et al. (2003). Improving the *Arabidopsis*

genome annotation using maximal transcript alignment assemblies. Nucleic Acids Res. **31**:5654-5666. https://doi.org/10.1093/nar/gkg770.

Holt, C., and Yandell, M. (2011). MAKER2: an annotation pipeline and genome-database management tool for second-generation genome projects. BMC Bioinf. **12**:491. https://doi.org/10.1186/1471-2105-12-491.

Hu, J., Fan, J., Sun, Z., and Liu, S. (2020). NextPolish: a fast and efficient genome polishing tool for long-read assembly. Bioinformatics **36**:2253-2255. https://doi.org/10.1093/bioinformatics/btz891.

Hu, X.G., Liu, H., Jin, Y., Sun, Y.Q., Li, Y., Zhao, W., El-Kassaby, Y.A., Wang, X.R., and Mao, J.F. (2016). *De novo* transcriptome assembly and characterization for the widespread and stress-tolerant conifer *Platycladus orientalis*. PLoS One **11**:e0148985. https://doi.org/10.1371/journal.pone.0148985.

Huerta-Cepas, J., Forslund, K., Coelho, L.P., Szklarczyk, D., Jensen, L.J., von Mering, C., and Bork, P. (2017). Fast genome-wide functional annotation through orthology assignment by eggNOG-Mapper. Mol. Biol. Evol. **34**:2115-2122. https://doi.org/10.1093/molbev/msx148.

Jin, Y., Zhao, W., Nie, S., Liu, S.S., El-Kassaby, Y.A., Wang, X.R., and Mao, J.F. (2019). Genome-wide variant identification and high-density genetic map construction using RADseq for *Platycladus orientalis* (Cupressaceae). G3: Genes, Genomes, Genet. **9**:3663-3672. https://doi.org/10.1534/g3.119.400684.

Kim, D., Langmead, B., and Salzberg, S.L. (2015). HISAT: a fast spliced aligner with low memory requirements. Nat. Methods **12**:357-360. https://doi.org/10.1038/nmeth.3317.

Koren, S., Walenz, B.P., Berlin, K., Miller, J.R., Bergman, N.H., and Phillippy, A.M. (2017). Canu: scalable and accurate long-read assembly via adaptive k-mer weighting and repeat separation. Genome Res. **27**:722-736. https://doi.org/10.1101/gr.215087.116.

Lamesch, P., Berardini, T.Z., Li, D., Swarbreck, D., Wilks, C., Sasidharan, R., Muller, R., Dreher, K., Alexander, D.L., Garcia-Hernandez, M., et al. (2012). The Arabidopsis Information Resource (TAIR): improved gene annotation and new tools. Nucleic Acids Res. **40**:D1202-1210. https://doi.org/10.1093/nar/gkr1090.

Li, H. (2018). Minimap2: pairwise alignment for nucleotide sequences. Bioinformatics **34**:3094-3100.

Liu, H., Wu, S., Li, A., and Ruan, J. (2021a). SMARTdenovo: a *de novo* assembler using long noisy reads. GigaByte **2021**:gigabyte15. https://doi.org/10.46471/gigabyte.15.

Liu, H., Wang, X., Wang, G., Cui, P., Wu, S., Ai, C., Hu, N., Li, A., He, B., Shao, X., et al. (2021b). The nearly complete genome of *Ginkgo biloba* illuminates gymnosperm evolution. Nat. Plants **7**:748-756. https://doi.org/10.1038/s41477-021-00933-x.

Liu, Y., Wang, S., Li, L., Yang, T., Dong, S., Wei, T., Wu, S., Liu, Y., Gong, Y., Feng, X., et al. (2022). The *Cycas* genome and the early evolution of seed plants. Nat. Plants **8**:389-401. https://doi.org/10.1038/s41477-022-01129-7.

Marçais, G., and Kingsford, C. (2011). A fast, lock-free approach for efficient parallel counting of occurrences of *k*-mers. Bioinformatics **27**:764-770. https://doi.org/10.1093/bioinformatics/btr011.

Mendes, F.K., Vanderpool, D., Fulton, B., and Hahn, M.W. (2021). CAFE 5 models variation in evolutionary rates among gene families. Bioinformatics **36**:5516-5518.

https://doi.org/10.1093/bioinformatics/btaa1022.

Neale, D.B., McGuire, P.E., Wheeler, N.C., Stevens, K.A., Crepeau, M.W., Cardeno, C., Zimin, A.V., Puiu, D., Pertea, G.M., Sezen, U.U., et al. (2017). The Douglas-fir genome sequence rreveals specialization of the photosynthetic apparatus in Pinaceae. G3: Genes, Genomes, Genet. **7**:3157-3167. https://doi.org/10.1534/g3.117.300078.

Nevers, Y., Warwick Vesztrocy, A., Rossier, V., Train, C.-M., Altenhoff, A., Dessimoz, C., and Glover, N.M. (2024). Quality assessment of gene repertoire annotations with OMArk. Nat. Biotechnol.:1-10. https://doi.org/10.1038/s41587-024-02147-w.

Nguyen, L.T., Schmidt, H.A., von Haeseler, A., and Minh, B.Q. (2015). IQ-TREE: a fast and effective stochastic algorithm for estimating maximum-likelihood phylogenies. Mol. Biol. Evol. **32**:268-274. https://doi.org/10.1093/molbev/msu300.

Niu, S., Li, J., Bo, W., Yang, W., Zuccolo, A., Giacomello, S., Chen, X., Han, F., Yang, J., Song, Y., et al. (2022). The Chinese pine genome and methylome unveil key features of conifer evolution. Cell **185**:204-217 e214. https://doi.org/10.1016/j.cell.2021.12.006.

Pertea, M., Pertea, G.M., Antonescu, C.M., Chang, T.C., Mendell, J.T., and Salzberg, S.L. (2015). StringTie enables improved reconstruction of a transcriptome from RNA-seq reads. Nat. Biotechnol. **33**:290-295. https://doi.org/10.1038/nbt.3122.

Pryszcz, L.P., and Gabaldon, T. (2016). Redundans: an assembly pipeline for highly heterozygous genomes. Nucleic Acids Res. **44**:e113. https://doi.org/10.1093/nar/gkw294.

Ren, R., Wang, H., Guo, C., Zhang, N., Zeng, L., Chen, Y., Ma, H., and Qi, J. (2018). Widespread whole genome duplications contribute to genome complexity and species diversity in angiosperms. Mol. Plant **11**:414-428. https://doi.org/10.1016/j.molp.2018.01.002.

Scott, A.D., Zimin, A.V., Puiu, D., Workman, R., Britton, M., Zaman, S., Caballero, M., Read, A.C., Bogdanove, A.J., Burns, E., et al. (2020). A reference genome sequence for giant sequoia. G3: Genes, Genomes, Genet. **10**:3907-3919. https://doi.org/10.1534/g3.120.401612.

Simão, F.A., Waterhouse, R.M., Ioannidis, P., Kriventseva, E.V., and Zdobnov, E.M. (2015). BUSCO: assessing genome assembly and annotation completeness with single-copy orthologs. Bioinformatics **31**:3210-3212. https://doi.org/10.1093/bioinformatics/btv351.

Slater, G.S., and Birney, E. (2005). Automated generation of heuristics for biological sequence comparison. BMC Bioinf. **6**:31. https://doi.org/10.1186/1471-2105-6-31.

Song, C., Fu, F., Yang, L., Niu, Y., Tian, Z., He, X., Yang, X., Chen, J., Sun, W., Wan, T., et al. (2021). *Taxus yunnanensis* genome offers insights into gymnosperm phylogeny and taxol production. Commun. Biol. **4**:1203. https://doi.org/10.1038/s42003-021-02697-8.

Stanke, M., Diekhans, M., Baertsch, R., and Haussler, D. (2008). Using native and syntenically mapped cDNA alignments to improve *de novo* gene finding. Bioinformatics **24**:637-644. https://doi.org/10.1093/bioinformatics/btn013.

Stevens, K.A., Wegrzyn, J.L., Zimin, A., Puiu, D., Crepeau, M., Cardeno, C., Paul, R., Gonzalez-Ibeas, D., Koriabine, M., Holtz-Morris, A.E., et al. (2016). Sequence of the sugar pine megagenome. Genetics **204**:1613-1626. https://doi.org/10.1534/genetics.116.193227.

Sun, P., Jiao, B., Yang, Y., Shan, L., Li, T., Li, X., Xi, Z., Wang, X., and Liu, J. (2022). WGDI: A user-friendly toolkit for evolutionary analyses of whole-genome duplications and

ancestral karyotypes. Mol. Plant **15**:1841-1851. https://doi.org/10.1016/j.molp.2022.10.018.

Tang, H., Bowers, J.E., Wang, X., Ming, R., Alam, M., and Paterson, A.H. (2008). Synteny and collinearity in plant genomes. Science **320**:486-488. https://doi.org/10.1126/science.1153917.

Wan, T., Liu, Z., Leitch, I.J., Xin, H., Maggs-Kolling, G., Gong, Y., Li, Z., Marais, E., Liao, Y., Dai, C., et al. (2021). The *Welwitschia* genome reveals a unique biology underpinning extreme longevity in deserts. Nat. Commun. **12**:4247. https://doi.org/10.1038/s41467-021-24528-4.

Wan, T., Liu, Z.M., Li, L.F., Leitch, A.R., Leitch, I.J., Lohaus, R., Liu, Z.J., Xin, H.P., Gong, Y.B., Liu, Y., et al. (2018). A genome for gnetophytes and early evolution of seed plants. Nat. Plants **4**:82-89. https://doi.org/10.1038/s41477-017-0097-2.

Wang, D., Zhang, Y., Zhang, Z., Zhu, J., and Yu, J. (2010). KaKs_Calculator 2.0: a toolkit incorporating gamma-series methods and sliding window strategies. Genomics Proteomics Bioinformatics **8**:77-80. https://doi.org/10.1016/S1672-0229(10)60008-3.

Wang, Y., Tang, H., Debarry, J.D., Tan, X., Li, J., Wang, X., Lee, T.H., Jin, H., Marler, B., Guo, H., et al. (2012). MCScanX: a toolkit for detection and evolutionary analysis of gene synteny and collinearity. Nucleic Acids Res. **40**:e49. https://doi.org/10.1093/nar/gkr1293.

Xie, J., Chen, Y., Cai, G., Cai, R., Hu, Z., and Wang, H. (2023). Tree Visualization By One Table (tvBOT): a web application for visualizing, modifying and annotating phylogenetic trees. Nucleic Acids Res. **51**:W587-W592. https://doi.org/10.1093/nar/gkad359.

Xiong, X., Gou, J., Liao, Q., Li, Y., Zhou, Q., Bi, G., Li, C., Du, R., Wang, X., and Sun, T. (2021). The *Taxus* genome provides insights into paclitaxel biosynthesis. Nat. Plants **7**:1026-1036. https://doi.org/10.1038/s41477-021-00963-5.

Yang, Z. (2007). PAML 4: phylogenetic analysis by maximum likelihood. Mol. Biol. Evol. **24**:1586-1591. https://doi.org/10.1093/molbev/msm088.

Zhang, Z., Xiao, J., Wu, J., Zhang, H., Liu, G., Wang, X., and Dai, L. (2012). ParaAT: a parallel tool for constructing multiple protein-coding DNA alignments. Biochem. Biophys. Res. Commun. **419**:779-781. https://doi.org/10.1016/j.bbrc.2012.02.101.

Supplemental Note 5. Molecular mechanisms of TE-mediated gene duplication

Abstract

Gene duplication is a fundamental driver of evolutionary innovation, and transposable elements (TEs) have emerged as powerful engines capable of duplicating, capturing, and relocating gene sequences through remarkably diverse molecular pathways. Here, we provide a comprehensive classification of TE-mediated gene duplication mechanisms based on extensive literature survey spanning bacteria, fungi, plants, insects, and mammals. We define TE-mediated gene duplication specifically as mechanisms that duplicate, amplify, or expand the existing gene space, particularly the protein-coding gene repertoire, where pre-existing host gene sequences are copied and relocated through TE activity. This definition explicitly excludes processes by which TEs contribute sequence material to create novel genes de novo, such as TE exonization, transposase domestication, TE-derived regulatory elements, and TE-derived noncoding RNAs.

We identify 12 distinct molecular mechanisms and organize them into a hierarchical classification framework comprising two domains and four categories. Domain A (Direct TE-Catalyzed Mechanisms) includes seven mechanisms where TE-encoded enzymes, reverse transcriptases, transposases, and Rep/Hel proteins, directly execute the duplication process: Category 1 (RNA-Mediated Duplication) encompasses retrogenesis, retrotransposon transduction, and retrotransposon template switching; Category 2 (Direct DNA Transposon-Mediated Capture) comprises Pack-TYPE transposons (fusion via internal deletion), Helitrons (rolling-circle replication read-through), composite transposons, and end bypass in TIR TEs. Domain B (TE-Facilitated Mechanisms) includes five mechanisms where TEs provide homology substrates or structural triggers acted upon by host cellular machinery: Category 3 (Homology-Directed Recombination/Repair) contains NAHR, LTR unequal recombination, and SDSA (gap-filling); Category 4 (Replication-Based Mechanisms) includes FoSTeS/MMBIR and FoSTeST. The existence of these 12 mechanisms arises from three fundamental sources: the diversity of TE types and their enzymatic machineries, multiple points of "leakiness" in TE life cycles that can capture host sequences, and the capacity of TEs to serve as substrates for host repair and replication machinery.

Despite the enormous diversity of TE types and transposition machineries, we identify key mechanistic interconnections that bridge these pathways. Template switching emerges as a unifying principle shared across both RNA-level (Mechanisms 3) and DNA-level (Mechanisms 11 and 12) processes, though with distinct molecular contexts, cytoplasmic reverse transcription for LINE-1, virus-like particle-enclosed reverse transcription for LTR elements, and replisome-mediated switching at stalled replication forks. Additional shared features include end bypass (Mechanisms 2, 5, 7, and implicit in 4, 6), microhomology guidance (Mechanisms 3, and 11, 12), and cross-domain hybrid mechanisms exemplified by FoSTeST (Mechanism 12), where a Domain B replication error creates a chimeric sequence that is subsequently amplified through Domain A transposition. This classification framework provides a foundation for systematic identification and functional characterization of TE-mediated duplicates across genomes, advancing our understanding of how transposable elements drive the evolution of gene repertoires.

1. Introduction

Gene duplication is a fundamental driver of evolutionary innovation, providing raw genetic material for the emergence of new functions (Long et al., 2003; Ohno, 1970). While whole-genome duplication, tandem duplication, and segmental duplication have long been recognized as major sources of gene copies, the contribution of transposable elements (TEs) to gene duplication has only been fully appreciated in the genomic era. TEs, once dismissed as "selfish DNA" or "junk", are now understood to be powerful engines of genomic innovation, capable of duplicating, capturing, shuffling, and relocating gene sequences through remarkably diverse molecular pathways (Feschotte and Pritham, 2007; Kaessmann et al., 2009).

Here, with extensive literature survey, we summarized, in total, 12 molecular mechanisms of TE-mediated gene duplication (**Supplemental Figure 38 and Supplemental Tables 41-43**), representing the current understanding of how TE-mediated gene duplication across domains of life. These mechanisms have been identified through decades of research spanning bacteria, yeast, plants, insects, and mammals, combining classical genetics, molecular biology, and comparative genomics. The diversity of these mechanisms reflects the extraordinary variety of TE types themselves, from bacterial insertion sequences to mammalian LINE elements, from plant Helitrons to composite transposons, each with distinct transposition machineries that can be co-opted or exploited for gene duplication.

1.1 What We Includes and Excludes

Scope Definition: What Includes - Amplification of Existing Gene Space

This classification framework focuses specifically on TE-mediated mechanisms that duplicate, amplify, or expand the existing gene space, particularly the protein-coding gene repertoire. The defining criterion is that pre-existing host gene sequences are copied and relocated, resulting in additional copies of genes or gene fragments that existed prior to the TE-mediated event.

The included mechanisms share the following characteristics, such as (1) A host gene (or gene fragment) that exists prior to the duplication event; (2) TE activity (either TE-encoded enzymes or TE-created substrates) that results in copying of the host gene sequence; (3) A new genomic copy of the host gene (complete or partial) at a different location; (4) Amplification or expansion of existing genetic information.

Scope Definition: What Excludes

Importantly, this our identification and classification of molecular mechanisms of TE-mediated gene duplication does not include mechanisms by which TEs contribute sequence material that becomes incorporated into novel genes. These alternative modes of TE-mediated gene evolution, while biologically important, involve de novo creation of genic sequences from TE sequences rather than duplication of pre-existing genes, such as TE exonization (Lev-Maor et al., 2003; Sorek, 2007; Sorek et al., 2002), transposase capture for transcription factors (TE Domestication) (Cosby et al., 2021; Kapitonov and Jurka, 2005), TE-derived regulatory elements (Gebrie, 2023; Ilık et al., 2025), solo-LTR regulatory function (Chen et al., 2024; Thompson et al., 2016), TE-derived ncRNAs (Kapusta et al., 2013; Panariello et al., 2025; Zhou et al., 2019), and transposase neofunctionalization (Alzohairy et al., 2013; Cosby *et al.*, 2021; Jangam et al., 2017).

1.2 Why 12 mechanisms? The Molecular Logic

The existence of 12 distinct mechanisms arises from three fundamental sources of variation (**Supplemental Figure 38 and Supplemental Tables 41-43**). First, one group of fundamental sources are the diversity of TE types and their enzymatic machineries. TEs are classified into two major classes based on their transposition intermediate (Kapitonov and Jurka, 2008; Wicker et al., 2007). Each TE type encodes distinct enzymatic activities that interact with host DNA via specific molecular pathways, resulting in gene duplication.

Second, multiple points of "leakiness" in TE life cycles are providing more fundamental variation sources. TEs have evolved streamlined life cycles for self-propagation, but these cycles contain multiple points where "errors" or "leakiness" can capture host sequences. For instance, the principle of "end bypass" was emphasized as one of three common features shared across TE-mediated duplication mechanisms (Ma et al., 2023). Also, LINE-1 reverse transcription can proceed via canonical poly(A) binding or through template switching when RT encounters obstacles, demonstrating how a single TE type can generate duplicates through multiple molecular routes (Ma *et al.*, 2023).

Third, TEs presents substrates for host machinery. Beyond their own enzymatic activities, TEs create genomic conditions that predispose to duplication by host cellular machinery.

2. Two-Domain Classification System

2.1 Domain A: Direct TE-Catalyzed Mechanisms

TE-encoded enzymes (reverse transcriptase, transposase, Rep/Hel proteins) directly execute the duplication process (Jiang et al., 2004; Kaessmann *et al.*, 2009).

Includes:

- Category 1: RNA-mediated duplication (Mechanisms 1–3)
- Category 2: Direct DNA transposon-mediated capture (Mechanisms 4–7)

2.2 Domain B: TE-Facilitated Mechanisms

TEs provide homology substrates, structural triggers, or architectural features that are then acted upon by host cellular machinery (Lee et al., 2007; Lupski, 1998).

Includes:

- Category 3: Homology-directed ectopic recombination/repair (Mechanisms 8–10)
- Category 4: Replication-based mechanisms (Mechanisms 11–12)

3. DOMAIN A: Direct TE-Catalyzed Mechanisms

3.1 Category 1: RNA-Mediated Duplication Mechanisms

These mechanisms require reverse transcription of RNA intermediates, producing predominantly intronless gene copies (Kaessmann *et al.*, 2009) (**Supplemental Figure 38 and Supplemental Tables 41-43**. During reverse transcription, intron-retaining gene copies can also be generated in the presence of antisense read-through transcription or weak splicing signals (Jiang et al., 2009; Xiao et al., 2008).

Mechanism 1: Retrogenesis (Processed Retrocopies)

General Mechanism / Molecular Pathway

L1 (LINE-1) retrotransposon-encoded proteins (ORF1p and ORF2p) bind mature, spliced mRNAs via their poly(A) tails in the cytoplasm (Hohjoh and Singer, 1996). The ribonucleoprotein complex is transported to the nucleus where L1 ORF2p endonuclease (EN) cleaves

chromosomal DNA at a consensus sequence (5'-TTTT/AA) (Feng et al., 1996). The ORF2p reverse transcriptase (RT) then generates cDNA copies through target-primed reverse transcription (TPRT), using the 3'-OH of the cleaved DNA as a primer (Luan et al., 1993). This creates processed pseudogenes or functional retrogenes at new genomic locations.

Core Molecular Trigger

- **Primary trigger**: LINE1 ORF2p reverse transcriptase and endonuclease activity (Feng *et al.*, 1996)
- **Template**: Mature, spliced, polyadenylated mRNA
- **Integration**: Target-primed reverse transcription (TPRT) at L1 EN cleavage sites (Luan *et al.*, 1993)
- **Cis preference**: L1 machinery shows strong cis preference for L1 mRNA, but can act in trans on cellular mRNAs (Wei et al., 2001)

Typical Genomic Features / Signatures

For Pathway A (Poly(A) binding):

- Complete loss of introns (processed from mature mRNA)
- Poly(A) tail remnant at 3' end
- Target site duplications (TSDs) flanking the insertion
- Absence of upstream regulatory elements (promoterless unless acquiring new promoter)

Evolutionary Outcome

- **Gene innovation**: 575 potentially functional retrogenes in human genome (Vinckenbosch et al., 2006)
- **"Out-of-X" pattern**: Strong bias for X-linked parental genes producing autosomal retrogenes, driven by meiotic sex chromosome inactivation (MSCI) (Emerson et al., 2004; Potrzebowski et al., 2008)
- **Testis expression bias**: Many retrogenes acquire testis-specific expression (Vinckenbosch *et al.*, 2006)
- **Neofunctionalization**: Can acquire new functions through subcellular relocalization (Rosso et al., 2008)

Case Studies

Gene	Species	Details	Reference
jingwei	*D. yakuba*	Adh-derived retrogene inserted into *yande* intron ~2.5 Mya; testis-specific; under positive selection; chimeric structure	(Long and Langley, 1993)
CDC14Bretro	Hominoid primates	Retroposed 18–25 Mya from CDC14B; brain/testis expression; underwent subcellular relocalization to ER membranes; positive selection	(Rosso *et al.*, 2008)
Pgk-2	Mouse/mammals	Autosomal intronless copy of X-linked Pgk-1; testis-specific expression compensates for MSCI	(McCarrey and Thomas, 1987)
sphinx	*Drosophila melanogaster*	Male-specific chimeric gene formed by retroposition + exon shuffling; novel reproductive function	(Wang et al., 2002)

Mechanism 2: Retrotransposon Transduction (3' and 5')

General Mechanism / Molecular Pathway

Retrotransposon transduction occurs when L1, SVA, or LTR retrotransposons mobilize flanking genomic sequences during retrotransposition. This represents "end bypass" at either the 3' or 5' end of the element (Kazazian and Goodier, 2002; Ma *et al.*, 2023).

3' Transduction (Common): Retrotransposon transcription initiates from an internal promoter but bypasses the element's weak polyadenylation signal, continuing transcription into downstream genomic flanking sequences (Holmes et al., 1994; Moran et al., 1999).

5' Transduction (Rare): When a retrotransposon's internal promoter is silenced or weak, transcription may initiate from an upstream host gene promoter, producing a chimeric transcript that includes upstream genomic sequence followed by the retrotransposon (Cordaux and Batzer, 2009; Kazazian and Goodier, 2002). This chimeric transcript undergoes reverse transcription and integration, carrying the upstream sequences to the new insertion site.

Note: SVA (SINE-VNTR-Alu) transposons are relatively young, active, hominid-specific mobile genetic elements in the human genome, made of repetitive sequences (hexamer, Alu-like, VNTR, SINE-R) that can copy and insert themselves, often using LINE-1 machinery, contributing to genetic variation and disease by disrupting or regulating genes, like in X-linked dystonia parkinsonism (XDP) or muscular dystrophies.

Core Molecular Trigger

- **3' Transduction trigger**: Weak or bypassed polyadenylation signal at retrotransposon 3' end (Moran *et al.*, 1999)
- **5' Transduction trigger**: Upstream host promoter drives transcription of retrotransposon (Kazazian and Goodier, 2002)
- **Read-through transcription**: RNA polymerase II continues into flanking genomic DNA
- **Co-mobilization**: Flanking sequences are reverse-transcribed and integrated together with the element

Typical Genomic Features / Signatures

3' Transduction:

- Retrotransposon sequence (LINE, SVA, or LTR element) upstream of transduced material
- TSDs flanking the entire insertion (including transduced sequence)
- May include exons, introns, promoters, or regulatory elements from flanking genes
- 5' and 3' LTRs flanking captured genomic material (LTR-RT 3' Transduction)
- Intron structure can be retained (antisense read-through or weak splicing signals)
- Multiple genes possible in single transduction event

5' Transduction:

- Upstream genomic sequence at 5' end of transposon
- TSDs flanking entire insertion (upstream sequence + transposon)
- May include promoter/regulatory elements
- Transposon internal promoter typically inactive
- Much rarer than 3' transduction

Evolutionary Outcome

- **Genome expansion**: ~0.6–1% of human DNA derives from L1 transduction (Pickeral et al. 2000)

- **Gene family creation**: Can duplicate entire genes (e.g., AMAC family) (Xing et al., 2006)
- **Regulatory innovation**: Transduced sequences may include promoters/enhancers
- **Exon shuffling**: Can relocate coding exons to new genomic contexts (Moran *et al.*, 1999)
- **Promoter mobilization** (5'): Can relocate regulatory elements to new loci

Case Studies

Element/Gene	Type	Species	Details	Reference
**AMAC gene family**	3'	Human	SVA transduction duplicated entire AMAC gene creating multi-copy family	(Xing *et al.*, 2006)
***sun* locus**	3'	Tomato	LTR retrotransposon Rider 3' transduction affects fruit shape through IQ67 domain gene duplication	(Jiang *et al.*, 2009; Xiao *et al.*, 2008)
**L1 transduction**	3'	Human	L1 insertions carry transduced sequences	(Moran *et al.*, 1999)

Mechanism 3: Retrotransposon-Mediated Template Switching

General Mechanism / Molecular Pathway

During LTR retrotransposon replication, two RNA copies are co-packaged into virus-like particles. During reverse transcription, the reverse transcriptase can switch templates between the two RNA copies (copy-choice recombination), creating chimeric cDNAs (Derr et al., 1991). When cellular mRNAs are co-packaged with retrotransposon RNA, or when template switching captures flanking sequences, gene sequences can be duplicated and reshuffled.

In addition to the classical poly(A) binding mechanism, LINE-1 can also capture host sequences through template switching during reverse transcription. When L1 RT encounters secondary structures or pauses during cDNA synthesis, it can switch to a nearby RNA template (particularly small nuclear RNAs) and continue synthesis, creating chimeric retrocopies. This typically attaches host sequences to the 5' end of LINE-1 elements via microhomology (2-10 bp) at the fusion junction (Buzdin et al., 2002). Eighty-one such chimeras have been identified in the human genome, with 93% containing small nuclear RNA sequences (especially U6 snRNA) at their 5' ends, suggesting spatial proximity between L1 reverse transcription and the splicing machinery.

Core Molecular Trigger

- **Primary trigger**: Low template affinity of retroviral RT allows template switching (Goodrich and Duesberg, 1990)
- **Copy-choice recombination**: RT switches between co-packaged RNA templates, LINE1 RT capture host sequences (especially snRNAs) at 5' end
- **Microhomology**: Short homologous sequences guide template switching

Typical Genomic Features / Signatures

- Mosaic element structure with sequences from different sources
- Microhomology at recombination junctions
- Shuffled exons from different genes possible
- Intronless or intron-containing products
- Preferential capture of snRNAs (LINE1)

Evolutionary Outcome

- **Gene capture**: Occasional capture of host gene sequences
- **Exon shuffling**: Can combine exons from different genes

Case Studies

Element/Gene	Species	Details	Reference
**Bs1 retrotransposon**	Maize	Chimeric element containing fragments from three genes through template switching	(Elrouby and Bureau, 2010)
**LTR retrocopies**	*D. melanogaster*	15 LTR-mediated retrocopies identified; microhomology at junctions	(Tan et al., 2016)

3.2 Category 2: Direct DNA Transposon-Mediated Capture

These mechanisms involve DNA transposons physically capturing gene fragments during DNA-level transposition events. Products characteristically retain introns (Jiang *et al.*, 2004) (**Supplemental Figure 38 and Supplemental Tables 41-43**).

Mechanism 4: Pack-TYPE Transposons (Fusion via internal deletion)

General Mechanism / Molecular Pathway

Pack-TYPE elements are a kind of Class II non-autonomous TE discovered in plants that contain DNA fragments captured from coding genes between their TIR sequences (Catoni et al., 2019; Gisby and Catoni, 2022; Jiang *et al.*, 2004). The formation of Pack-TYPE elements involves: (1) Tandem or nearby insertion of two TIR TEs flanking a gene region, (2) Deletion, mutation or loss of internal sequences in TIR TEs, (3) Intervening genomic DNA (including gene fragments) is captured between the outer TIRs, (4) Transposase from autonomous elements mobilizes the entire structure including captured sequences (Catoni *et al.*, 2019; Jiang *et al.*, 2004).

This mechanism has been demonstrated across multiple TIR transposon superfamilies, leading to the generalization from "Pack-MULEs" to "Pack-TYPE" elements (Gisby and Catoni, 2022).

TE Superfamilies Involved

Main: *MULE, CACTA, hAT, PIF-Harbinger, TC1-Mariner*

Core Molecular Trigger

- **Primary trigger**: Transposase recognizing outer TIRs after internal TE sequences loss (Jiang *et al.*, 2004)
- **Mobilization**: Standard cut-and-paste transposition of entire captured structure
- **Expression**: at least 22% of Pack-MULEs in rice are transcribed (Hanada et al., 2009)

Typical Genomic Features / Signatures

- Superfamily-specific TIRs at element termini
- TSDs flanking the element (length specific to superfamily)
- Gene fragments (often partial) with introns retained (DNA-level capture)
- Multiple gene fragments possible (~20% of Pack-MULEs contain fragments from >1 gene) (Jiang *et al.*, 2004)
- Internal sequences lack transposase ORF

Evolutionary Outcome

- **Massive gene shuffling**: >3,000 Pack-MULEs in rice capturing fragments from >1,000

genes (Jiang *et al.*, 2004)

- **Transcription**: ~40% of Pack-MULEs transcribed; 9% ribosome-associated (Zhao et al., 2018)
- **Purifying selection**: Subset under selective constraint suggesting functionality (Hanada *et al.*, 2009)
- **Chimeric transcripts**: Chimeric Pack-MULEs more frequently expressed (Hanada *et al.*, 2009)

Case Studies

System	Species	Details	Reference
**Pack-MULEs**	Rice (*Oryza sativa*)	>3,000 elements; fragments from >1,000 genes; pioneering discovery	(Jiang *et al.*, 2004)
**Pack-MULEs functional**	Rice	~22% transcribed; 28 with direct translation evidence; purifying selection	(Hanada *et al.*, 2009)
**Pack-CACTAs**	*Arabidopsis*	First real-time mobilization observed in epiRILs; captured gene fragments mobilized	(Catoni *et al.*, 2019)
**Pack-TYPEs**	Rice/Maize	>10,000 Pack-TYPE elements across superfamilies (MULE, CACTA, hAT, PIF, Mariner)	(Gisby and Catoni, 2022)

Mechanism 5: Helitrons (Rolling-circle replication read-through)

General Mechanism / Molecular Pathway

Helitrons transpose via rolling-circle replication mediated by Rep/Hel proteins (Kapitonov and Jurka, 2001). The Rep protein nicks the 5'-TC terminus, initiates rolling-circle replication, and should terminate at the 3'-CTRR hairpin structure. When termination fails, replication continues into flanking genomic DNA, capturing gene sequences (Morgante et al., 2005). The captured sequences are then transposed to new locations.

Core Molecular Trigger

- **Primary trigger**: Termination failure at 3'-CTRR hairpin (Kapitonov and Jurka, 2001)
- **Rolling-circle replication**: Rep protein-mediated strand displacement synthesis
- **Stepwise capture**: Subsequent termination failures can add additional gene sequences (Dong et al., 2011)

Typical Genomic Features / Signatures

- **NO target site duplications** (key distinguishing feature from TIR transposons)
- No terminal inverted repeats
- Frequent multi-gene capture (Dong *et al.*, 2011; Morgante *et al.*, 2005)

Evolutionary Outcome

- **Massive gene capture**: ~2% of maize genome; captures fragments from hunderds genes (Morgante *et al.*, 2005)
- **Intraspecific diversity**: Major contributor to presence/absence variation between maize lines
- **Exon shuffling**: Creates novel gene combinations
- **Thousands new transcripts estimated in maize** (Du et al., 2009)
- **Recurrent transposition**: Creates hundreds of copies of captured genes (Ma *et al.*, 2023)

Case Studies

System	Species	Details	Reference
**Maize Helitrons**	*Zea mays*	Thousands of elements; ~2% of genome; >20,000 gene fragments; thousands of intact	(Du *et al.*, 2009; Morgante *et al.*, 2005)
**HelibatN217.1**	Bat *Myotis lucifugus*	Single Helitrons duplicating 392 copies of TMBIM4 gene fragment	(Thomas et al., 2014)
**HelibatN3**	Bat *Myotis*	>1,000 copies of NUBPL gene captured	(Pritham and Feschotte, 2007)

Mechanism 6: Composite Transposons

General Mechanism / Molecular Pathway

Composite transposons form when two insertion sequence (IS) elements flank a segment of DNA containing passenger genes (Kleckner, 1981). The transposase recognizes the outermost inverted repeats (IRs) of the flanking IS elements and mobilizes the entire structure, including the central passenger genes, as a single unit (Siguier et al., 2014).

Core Molecular Trigger

- **Primary trigger**: Transposase recognition of outermost IRs from flanking IS elements
- **Composite structure**: Central DNA + flanking IS elements move as unit

Typical Genomic Features / Signatures

- Paired IS elements at termini
- TSDs flanking the complete composite structure
- Central passenger genes (often antibiotic resistance genes)
- Hybrid promoters possible at IS-gene junctions

Evolutionary Outcome

- **Antibiotic resistance spread**: Major mechanism for dissemination of resistance genes
- **Horizontal gene transfer**: Mobilizes genes between species and genera
- **Bacterial genome evolution**: Major driver of plasticity in bacterial genomes

Case Studies

Element	Organism	Details	Reference
**Tn5**	*E. coli*	Flanking IS50 elements; carries kanamycin/bleomycin/streptomycin resistance	(Kleckner, 1981)
**Tn10**	Bacteria	Flanking IS10 elements; tetracycline resistance; well-characterized	(Haniford, 2006)
**IS*Apf1***	Bacteria	mcr-1 colistin resistance gene flanked by IS; global spread	(Wang et al., 2018)

Mechanism 7: End Bypass in TIR Transposable Elements

General Mechanism / Molecular Pathway

Transposases may misrecognize terminal sequences, using cryptic TIRs in flanking DNA or TIRs from different elements (macrotransposition), leading to capture of flanking sequences beyond the normal element boundaries (Zhang et al., 2009b; Zhang et al., 2006). This can also occur when transposase recognizes the TIR of one element and a cryptic TIR-like sequence in an adjacent element or flanking region.

Core Molecular Trigger

- **Primary trigger**: Cryptic TIR recognition by transposase (Zhang *et al.*, 2006)
- **Macrotransposition**: Alternative end pairing captures intervening DNA

- **End bypass**: One of three common features of TE-mediated gene duplication (Ma *et al.*, 2023)

Typical Genomic Features / Signatures

- TIRs at termini (may include non-canonical sequences)
- TSDs flanking insertion
- Captured sequences beyond canonical element boundaries
- Can create Pack-TIR-like structures

Evolutionary Outcome

- **Gene fragment capture**: Creates structures similar to Pack-TIRs (Tan et al., 2021)
- **Genomic rearrangement**: Macrotransposition can affect up to 60 kb (Zhang *et al.*, 2006)
- **Novel gene fusions**: Captured sequences may fuse with element-derived sequences

Case Studies

System	Species	Details	Reference
**Ac/Ds macrotransposition**	Maize	Macrotransposition up to 60 kb	(Zhang *et al.*, 2006)

4. DOMAIN B: TE-Facilitated Mechanisms

4.1 Category 3: Homology-Directed Ectopic Recombination/Repair

These mechanisms utilize TEs as homology substrates for host recombination and repair machinery (Lupski, 1998).

Mechanism 8: NAHR (Non-Allelic Homologous Recombination)

General Mechanism / Molecular Pathway

During meiosis, programmed double-strand breaks (DSBs) occur at recombination hotspots. The 3' single-stranded DNA tails are coated with Rad51/Dmc1 recombinases and invade homologous sequences (Sasaki et al., 2010). When homologous sequences are present at non-allelic positions (due to LCRs or TE clusters), misalignment can occur, leading to Holliday junction formation and resolution that produces deletions, duplications, or inversions (Lupski, 1998).

Core Molecular Trigger

- **Primary trigger**: Misalignment at non-allelic homologous sequences during meiotic recombination
- **Requirements**: large repeats, or sequence identity over large stretches of DNA
- **Host machinery**: Rad51/Dmc1 recombinases, Holliday junction resolvases

Typical Genomic Features / Signatures

- Large segmental duplications/deletions
- Breakpoints within low-copy repeats (LCRs) or TE clusters
- Recurrent rearrangements (same breakpoints in unrelated individuals)
- Long homology tracts at breakpoints
- Reciprocal products possible (duplication ↔ deletion)

Evolutionary Outcome

- **Genomic disorders**: Recurrent CNVs cause specific disease phenotypes
- **Gene family expansion**: Can duplicate entire genes or gene clusters
- **Population CNV variation**: Major source of structural variation

- **Cancer predisposition**: Predisposes to somatic rearrangements

Case Studies

Locus	Species	Details	Reference
**17p12 PMP22**	human	1.4 Mb duplication/deletion; 24-kb CMT1A-REPs with 99% identity; the Charcot-Marie-Tooth type 1A paralogous repeats (CMT1A-REPs) themselves are segments of repetitive DNA sequence, but they are not functional, active transposons in the human genome today. Instead, they contain the fossilized remnants of a mariner-like transposable element (TE)	(Inoue et al., 2001; Kiyosawa and Chance, 1996)
**multiple**	human	LINEs and HERVs mediated, recurrent deletion and duplication rearrangements at four loci	(Campbell et al., 2014)
**multiple**	human	516 (Copy-number variation) CNVs potentially mediated by LINEs	(Startek et al., 2015)
**MATE1**	maize	Tandem duplication of the MATE1 gene occurred twice, likely mediated by retrotransposons	(Maron et al., 2013)

Mechanism 9: LTR-RT Unequal Recombination (Solo-LTR Formation)

General Mechanism / Molecular Pathway

LTR retrotransposons possess two identical or near-identical LTR sequences. Intra-element recombination between the 5' and 3' LTRs removes the internal region, leaving a solo-LTR (Vitte and Panaud, 2003). Inter-element recombination between LTRs of different copies can delete or duplicate intervening sequences, including host genes (Vitte and Bennetzen, 2006; Vitte and Panaud, 2003).

Core Molecular Trigger

- **Primary trigger**: Homologous recombination between LTR sequences
- **Intra-element**: 5' LTR × 3' LTR → solo-LTR + circular excision product
- **Inter-element**: LTR of element 1 × LTR of element 2 → deletion/duplication of intervening sequences

Typical Genomic Features / Signatures

- **Gene deletions/duplications between LTR copies**
- **Genome size reduction**: Counterbalances LTR retrotransposon amplification (Vitte and Panaud, 2003)
- **Gene loss**: Recombination between flanking LTRs can delete intervening genes
- **Regulatory variation**: Solo-LTRs retain enhancer functions
- **Genome turnover**: Major mechanism for genome contraction in plants

Case Studies

Gene	Species	Details	Reference
Rdl	Drosophila melanogaster	The 113-kb duplication contains one WT copy of Rdl and a second copy with two point mutations;	(Remnant et al.,

		ectopic recombination between *Roo* LTR-RT is involved in generating this genomic rearrangement	2013)

Mechanism 10: SDSA (Gap-Filling)

General Mechanism / Molecular Pathway

When a transposon inserts or breaks, it creates a double-strand break (DSB). The break is processed by 5'→3' resection, creating 3' single-stranded DNA tails. These tails can invade ectopic homologous sequences (rather than the sister chromatid), and synthesis-dependent strand annealing (SDSA) copies information from the ectopic template to the excision site (Gloor et al., 1991; Nassif et al., 1994). This can result in gene conversion events that copy sequences from distant genomic locations (Hedges and Deininger, 2007; Wicker et al., 2010).

Core Molecular Trigger

- **Primary trigger**: DSB created by transposon insertion or breakage
- **Template search**: 3' ssDNA tails search for homologous sequences
- **Ectopic template**: Non-allelic homologous sequence serves as repair template
- **SDSA pathway**: Synthesis-dependent strand annealing (non-crossover outcome)

Typical Genomic Features / Signatures

- Gene conversion tracts
- Ectopic sequence copied to new location
- No reciprocal exchange (SDSA mechanism)

Evolutionary Outcome

- **Gene conversion**: Can spread beneficial alleles
- **Sequence diversification**: Introduces variation from distant loci
- **Pack-TIR generation**: Gap-filling is transposition-independent mechanism for single-copy Pack-TIR formation (Ma *et al.*, 2023; Tan *et al.*, 2021)

Case Studies

System	Species	Details	Reference
**P-element/white locus**	*Drosophila*	Nearly 8,000 bp copied from ectopic template	(Nassif *et al.*, 1994)
**P-element gene targeting**	*Drosophila*	Foundation for gene replacement; homologous sequences used as repair template	(Gloor *et al.*, 1991)
**Bradi1g24450**	*monocotyledon*	Fillering DNA capture of a genomic fragment containing Bd1g24450during the repair of a Mutator-induced double-strand break	(Wicker *et al.*, 2010)

4.2 Category 4: Replication-Based Mechanisms

These mechanisms arise from errors during DNA replication, facilitated by TE-associated genomic complexity (Hastings et al., 2009; Lee *et al.*, 2007).

Mechanism 11: FoSTeS/MMBIR

General Mechanism / Molecular Pathway

When a replication fork encounters complex genomic architecture (LCRs, palindromes, TE-rich regions), it may stall (Lee *et al.*, 2007). The 3' end of the lagging strand can detach from its template and invade a nearby replication fork using microhomology (Gu et al., 2015). DNA

synthesis continues on the new template, and this process can occur serially (FoSTeS ×2, FoSTeS ×3), resulting in complex rearrangements (Hastings *et al.*, 2009).
MMBIR (microhomology-mediated break-induced replication) is a related mechanism that can occur when a broken fork is repaired using microhomology-guided template invasion (Hastings *et al.*, 2009).

TE Connection

Complex TE-rich architecture promotes fork stalling; TEs provide microhomology substrates. (Gu *et al.*, 2015).

Core Molecular Trigger

- **Primary trigger**: Replication fork stalling at complex architecture
- **Template switch**: 3' lagging strand invades new template via microhomology
- **Serial switching**: FoSTeS ×2, ×3 creates increasing complexity

Typical Genomic Features / Signatures

- Short microhomology at junctions (distinguishes from NAHR)
- Templated insertions from distant sequences
- Interspersed normal and amplified segments

Evolutionary Outcome

- **Genomic disorders**: Nonrecurrent CNVs causing disease
- **Complex rearrangements**: Much more complex than simple deletions/duplications
- **Exon duplication**: Can duplicate individual exons
- **De novo gene structures**: Novel gene arrangements possible

Case Studies

Disorder	Locus	Details	Reference
**Pelizaeus-Merzbacher disease**	PLP1	Original FoSTeS discovery; complex duplications 200 kb–7 Mb	(Lee *et al.*, 2007)
**MECP2 duplication syndrome**	MECP2	27% show complex rearrangements; DUP-TRP/INV-DUP pattern	(Carvalho et al., 2009)
**Potocki-Lupski syndrome**	17p11.2	nonrecurrent duplications; complex	(Carvalho *et al.*, 2009)

Mechanism 12: FoSTeST (FoSTeS + Transposition)

General Mechanism / Molecular Pathway

FoSTeST represents a hybrid mechanism combining replication-based errors with transposition (Tan *et al.*, 2021). Such a molecular pathway may involve: (1) FoSTeS creates a chimeric sequence containing gene fragments through template switching; (2) This chimeric sequence acquires or becomes flanked by TIRs (through insertion or end bypass); (3) Transposase recognizes the TIRs and mobilizes the entire structure; (4) The Pack-TIR is then amplified through recurrent transposition.

Note: FoSTeST is a recently proposed mechanism (Tan *et al.*, 2021) with limited independent validation. Further experimental confirmation is needed.

Core Molecular Trigger

- **Primary trigger**: FoSTeS event creates initial chimeric structure
- **TIR acquisition**: Chimeric sequence acquires TIR flanking sequences
- **Transposition**: Transposase mobilizes the Pack-TIR structure
- **Amplification**: Recurrent transposition creates multiple copies

Typical Genomic Features / Signatures

- Pack-TIR structure (TIRs + gene fragments)
- Microhomology at internal junctions (FoSTeS signature)
- Multiple related copies (transposition amplification)
- TSDs flanking each insertion
- Replication-associated sequence complexity

Evolutionary Outcome

- **Multicopy amplification**: Pack-TIRs can reach dozens to hundreds of copies
- **New gene families**: Creates paralogous gene copies
- **Rapid evolution**: Multiple copies provide substrate for divergence
- **Neofunctionalization**: Some copies may acquire new functions

Case Studies

System	Species	Details	Reference
**Ssk-FB4 family**	*D. melanogaster*	Gene duplicated and captured by TIR transposon FB4; positive selection evidence; multiple copies	(Tan *et al.*, 2021)
**Multicopy Pack-TIRs**	100 animal genomes	370 Pack-TIRs identified; FoSTeST explains origin from single-copy precursors	(Tan *et al.*, 2021)

5. Interconnections and Boundaries

While the classification provides clear conceptual organization, the mechanisms are not entirely independent. We see overlaps among the 12 molecular mechanisms summarized here. And, some individual mechanisms are sequentially connected.

5. 1 Mechanistic Overlaps

Template switching: A broadly shared mechanism occurring in:

- **Mechanism 3 (Template Switching)**: 1. Template switching is mandatory for LTR retrotransposon-mediated retrocopies, occurring within virus-like particles (Goodrich and Duesberg, 1990; Tan *et al.*, 2016). 2. LINE-1 RT can switch templates during reverse transcription, capturing snRNAs and other host sequences at the 5' end via microhomology (Buzdin *et al.*, 2002).
- **Mechanisms 11–12 (FoSTeS/MMBIR, FoSTeST)**: Template switching at the DNA level during replication fork stalling

This reveals template switching as a **unifying principle** across both RNA-mediated and DNA replication-based gene duplication mechanisms, though with distinct molecular contexts (cytoplasmic RT for LINE-1, VLP-enclosed RT for LTR elements, replisome for FoSTeS).

End bypass: A common feature across retrotransposon transduction, Helitron and TIR TE capture (**Mechanisms 2, 4–7**) (Ma *et al.*, 2023)

Microhomology: Guides both template switching and FoSTeS/MMBIR (**Mechanisms 3, 11, 12**)

5.2 Sequential Mechanisms

- **FoSTeST (Mechanism 12)** explicitly combines a Domain B event (FoSTeS) with a Domain A event (transposition) (Tan *et al.*, 2021)
- **Recurrent transposition** can amplify products of any capture mechanism, as emphasized by Ma et al. (Ma *et al.*, 2023) (**Mechanisms 2-7, 12**).

5.3 Pathway flexibility within mechanisms: Some mechanisms can operate through multiple

molecular pathways. For instance, LINE-1-mediated retrogenesis can proceed via either direct poly(A) binding (TPRT) or template switching (Ma *et al.*, 2023). This flexibility may explain the diversity of retrocopy structures observed in genomes and suggests that mechanistic boundaries are sometimes permeable. The choice of pathway may depend on cellular conditions, RNA secondary structures, and spatial proximity of reverse transcriptase to potential template RNAs.

5.4 Organism-Specific Prevalence

- **Mammals**: RNA-mediated mechanisms (L1 retrogenesis, transduction) predominate (Kaessmann *et al.*, 2009)
- **Plants**: DNA capture mechanisms (Pack-TYPE, Helitrons) are more prominent (Gisby and Catoni, 2022; Jiang *et al.*, 2004; Morgante *et al.*, 2005)
- **Bacteria**: Composite transposons drive horizontal gene transfer (Siguier *et al.*, 2014)
- **Humans**: NAHR and FoSTeS/MMBIR underlie many genomic disorders (Zhang et al., 2009a)

6. Conclusion

Through extensive literature survey, we have summarized 12 molecular mechanisms of TE-mediated gene duplication, organized into a hierarchical two-domain, four-category classification framework. Domain A encompasses seven mechanisms where TE-encoded enzymes, reverse transcriptases, transposases, and Rep/Hel proteins, directly catalyze the duplication process, while Domain B includes five mechanisms where TEs provide substrates or structural triggers that are acted upon by host cellular machinery. This classification reflects the fundamental distinction between direct TE catalysis and TE facilitation, providing a conceptual framework for understanding the diverse pathways through which transposable elements contribute to gene duplication across all domains of life. Importantly, We identified that end bypass, template switching, and recurrent transposition are shared features across diverse molecular mechanisms.

The evolutionary significance of TE-mediated gene duplication extends beyond the mere copying of genetic material. The intrinsic properties of these mechanisms, particularly their propensity for exon shuffling, chimeric gene formation, and relocation to new genomic contexts, predispose duplicated genes toward neofunctionalization rather than simple dosage amplification or subfunctionalization. As genomic technologies continue to advance, we anticipate deeper characterization of both the molecular details underlying these mechanisms and the functional consequences of TE-mediated duplicates, particularly for understudied mechanisms such as retrotransposon-mediated retroposition and gene capture in plants. The framework presented here provides a foundation for systematic identification and classification of TE-mediated duplicates across genomes, contributing to our understanding of how these "controlling elements," as Barbara McClintock termed them, continue to shape the evolution of gene repertoires and drive phenotypic innovation.

References:

Albalat, R., and Canestro, C. (2016). Evolution by gene loss. Nat. Rev. Genet. **17**:379-391. https://doi.org/10.1038/nrg.2016.39.

Alzohairy, A.M., Gyulai, G., Jansen, R.K., and Bahieldin, A. (2013). Transposable

elements domesticated and neofunctionalized by eukaryotic genomes. Plasmid **69**:1-15. 10.1016/j.plasmid.2012.08.001.

Boratyn, G.M., Schaffer, A.A., Agarwala, R., Altschul, S.F., Lipman, D.J., and Madden, T.L. (2012). Domain enhanced lookup time accelerated BLAST. Biol. Direct **7**:12. https://doi.org/10.1186/1745-6150-7-12.

Buchfink, B., Xie, C., and Huson, D.H. (2015). Fast and sensitive protein alignment using DIAMOND. Nat. Methods **12**:59-60. https://doi.org/10.1038/nmeth.3176.

Buzdin, A., Ustyugova, S., Gogvadze, E., Vinogradova, T., Lebedev, Y., and Sverdlov, E. (2002). A new family of chimeric retrotranscripts formed by a full copy of U6 small nuclear RNA fused to the 3′ terminus of l1. Genomics **80**:402-406.

Camacho, C., Coulouris, G., Avagyan, V., Ma, N., Papadopoulos, J., Bealer, K., and Madden, T.L. (2009). BLAST+: architecture and applications. BMC Bioinf. **10**:421. https://doi.org/10.1186/1471-2105-10-421.

Campbell, I.M., Gambin, T., Dittwald, P., Beck, C.R., Shuvarikov, A., Hixson, P., Patel, A., Gambin, A., Shaw, C.A., Rosenfeld, J.A., et al. (2014). Human endogenous retroviral elements promote genome instability via non-allelic homologous recombination. BMC Biol **12**:74. 10.1186/s12915-014-0074-4.

Capella-Gutiérrez, S., Silla-Martínez, J.M., and Gabaldón, T. (2009). trimAl: a tool for automated alignment trimming in large-scale phylogenetic analyses. Bioinformatics **25**:1972-1973. https://doi.org/10.1093/bioinformatics/btp348.

Carvalho, C.M., Zhang, F., Liu, P., Patel, A., Sahoo, T., Bacino, C.A., Shaw, C., Peacock, S., Pursley, A., Tavyev, Y.J., et al. (2009). Complex rearrangements in patients with duplications of MECP2 can occur by fork stalling and template switching. Hum Mol Genet **18**:2188-2203. 10.1093/hmg/ddp151.

Catoni, M., Jonesman, T., Cerruti, E., and Paszkowski, J. (2019). Mobilization of Pack-CACTA transposons in Arabidopsis suggests the mechanism of gene shuffling. Nucleic Acids Res **47**:1311-1320. 10.1093/nar/gky1196.

Chen, D., Zhang, T., Chen, Y., Ma, H., and Qi, J. (2022). Tree2GD: a phylogenomic method to detect large-scale gene duplication events. Bioinformatics **38**:5317-5321. https://doi.org/10.1093/bioinformatics/btac669.

Chen, M., Huang, X., Wang, C., Wang, S., Jia, L., and Li, L. (2024). Endogenous retroviral solo-LTRs in human genome. Front. Genet. **15**:1358078. 10.3389/fgene.2024.1358078.

Chen, S., Zhou, Y., Chen, Y., and Gu, J. (2018). fastp: an ultra-fast all-in-one FASTQ preprocessor. Bioinformatics **34**:i884-i890. https://doi.org/10.1093/bioinformatics/bty560.

Cheng, J., Wang, X., Liu, X., Zhu, X., Li, Z., Chu, H., Wang, Q., Lou, Q., Cai, B., Yang, Y., et al. (2021). Chromosome-level genome of Himalayan yew provides insights into the origin and evolution of the paclitaxel biosynthetic pathway. Mol. Plant **14**:1199-1209. https://doi.org/10.1016/j.molp.2021.04.015.

Chin, C.S., Alexander, D.H., Marks, P., Klammer, A.A., Drake, J., Heiner, C., Clum, A., Copeland, A., Huddleston, J., Eichler, E.E., et al. (2013). Nonhybrid, finished microbial genome assemblies from long-read SMRT sequencing data. Nat. Methods **10**:563-569. https://doi.org/10.1038/nmeth.2474.

Cordaux, R., and Batzer, M.A. (2009). The impact of retrotransposons on human genome evolution. Nat. Rev. Genet. **10**:691-703. 10.1038/nrg2640.

Cosby, R.L., Judd, J., Zhang, R., Zhong, A., Garry, N., Pritham, E.J., and Feschotte, C. (2021). Recurrent evolution of vertebrate transcription factors by transposase capture. Science **371**:eabc6405. 10.1126/science.abc6405.

Derr, L.K., Strathern, J.N., and Garfinkel, D.J. (1991). RNA-mediated recombination in S. cerevisiae. Cell **67**:355-364. 10.1016/0092-8674(91)90187-4.

Dong, Y., Lu, X., Song, W., Shi, L., Zhang, M., Zhao, H., Jiao, Y., and Lai, J. (2011). Structural characterization of helitrons and their stepwise capturing of gene fragments in the maize genome. BMC Genomics **12**:609. 10.1186/1471-2164-12-609.

Du, C., Fefelova, N., Caronna, J., He, L., and Dooner, H.K. (2009). The polychromatic Helitron landscape of the maize genome. Proc. Natl. Acad. Sci. U. S. A. **106**:19916-19921. 10.1073/pnas.0904742106.

Duan, Z., Andronescu, M., Schutz, K., McIlwain, S., Kim, Y.J., Lee, C., Shendure, J., Fields, S., Blau, C.A., and Noble, W.S. (2010). A three-dimensional model of the yeast genome. Nature **465**:363-367. https://doi.org/10.1038/nature08973.

Edgar, R.C. (2004). MUSCLE: multiple sequence alignment with high accuracy and high throughput. Nucleic Acids Res. **32**:1792-1797. https://doi.org/10.1093/nar/gkh340.

Elrouby, N., and Bureau, T.E. (2010). Bs1, a New Chimeric Gene Formed by Retrotransposon-Mediated Exon Shuffling in Maize

Plant Physiol. **153**:1413-1424. 10.1104/pp.110.157420.

Emerson, J.J., Kaessmann, H., Betran, E., and Long, M. (2004). Extensive gene traffic on the mammalian X chromosome. Science **303**:537-540. 10.1126/science.1090042.

Emms, D.M., and Kelly, S. (2019). OrthoFinder: phylogenetic orthology inference for comparative genomics. Genome Biol. **20**:238. https://doi.org/10.1186/s13059-019-1832-y.

Feng, Q., Moran, J.V., Kazazian, H.H., Jr., and Boeke, J.D. (1996). Human L1 retrotransposon encodes a conserved endonuclease required for retrotransposition. Cell **87**:905-916. 10.1016/s0092-8674(00)81997-2.

Feschotte, C., and Pritham, E.J. (2007). DNA transposons and the evolution of eukaryotic genomes. Annu. Rev. Genet. **41**:331-368. 10.1146/annurev.genet.40.110405.090448.

Fu, L., Niu, B., Zhu, Z., Wu, S., and Li, W. (2012). CD-HIT: accelerated for clustering the next-generation sequencing data. Bioinformatics **28**:3150-3152. https://doi.org/10.1093/bioinformatics/bts565.

Fujino, T., Yamaguchi, K., Yokoyama, T.T., Hamanaka, T., Harazono, Y., Kamada, H., Kobayashi, W., Ujino-Ihara, T., Uchiyama, K., and Matsumoto, A. (2024). A chromosome-level genome assembly of a model conifer plant, the Japanese cedar, *Cryptomeria japonica* D. Don. BMC Genomics **25**:1039. https://doi.org/10.1186/s12864-024-10929-4.

Gebrie, A. (2023). Transposable elements as essential elements in the control of gene expression. Mobile DNA **14**:9.

Gisby, J.S., and Catoni, M. (2022). The widespread nature of Pack-TYPE transposons reveals their importance for plant genome evolution. PLoS Genet **18**:e1010078. 10.1371/journal.pgen.1010078.

Gloor, G.B., Nassif, N.A., Johnson-Schlitz, D.M., Preston, C.R., and Engels, W.R. (1991).

Targeted gene replacement in Drosophila via P element-induced gap repair. Science **253**:1110-1117. 10.1126/science.1653452.

Goodrich, D.W., and Duesberg, P.H. (1990). Retroviral recombination during reverse transcription. Proc. Natl. Acad. Sci. U. S. A. **87**:2052-2056. 10.1073/pnas.87.6.2052.

Grabherr, M.G., Haas, B.J., Yassour, M., Levin, J.Z., Thompson, D.A., Amit, I., Adiconis, X., Fan, L., Raychowdhury, R., Zeng, Q., et al. (2011). Full-length transcriptome assembly from RNA-Seq data without a reference genome. Nat. Biotechnol. **29**:644-652. https://doi.org/10.1038/nbt.1883.

Grotewold, E. (2006). The genetics and biochemistry of floral pigments. Annu. Rev. Plant Biol. **57**:761-780. https://doi.org/10.1146/annurev.arplant.57.032905.105248.

Gu, S., Yuan, B., Campbell, I.M., Beck, C.R., Carvalho, C.M., Nagamani, S.C., Erez, A., Patel, A., Bacino, C.A., Shaw, C.A., et al. (2015). Alu-mediated diverse and complex pathogenic copy-number variants within human chromosome 17 at p13.3. Hum Mol Genet **24**:4061-4077. 10.1093/hmg/ddv146.

Guan, R., Zhao, Y., Zhang, H., Fan, G., Liu, X., Zhou, W., Shi, C., Wang, J., Liu, W., Liang, X., et al. (2016). Draft genome of the living fossil *Ginkgo biloba*. GigaScience **5**:49. https://doi.org/10.1186/s13742-016-0154-1.

Guo, S., Ji, Y., Zheng, Y., Watkins, C.B., Ma, L., Wang, Q., Liang, H., Bai, C., Fu, A., Li, L., et al. (2023). Transcriptomic, metabolomic, and ATAC-seq analysis reveal the regulatory mechanism of senescence of post-harvest tomato fruit. Front. Plant Sci. **14**:1142913. https://doi.org/10.3389/fpls.2023.1142913.

Haas, B.J., Salzberg, S.L., Zhu, W., Pertea, M., Allen, J.E., Orvis, J., White, O., Buell, C.R., and Wortman, J.R. (2008). Automated eukaryotic gene structure annotation using EVidenceModeler and the Program to Assemble Spliced Alignments. Genome Biol. **9**:R7. https://doi.org/10.1186/gb-2008-9-1-r7.

Haas, B.J., Delcher, A.L., Mount, S.M., Wortman, J.R., Smith, R.K., Jr., Hannick, L.I., Maiti, R., Ronning, C.M., Rusch, D.B., Town, C.D., et al. (2003). Improving the *Arabidopsis* genome annotation using maximal transcript alignment assemblies. Nucleic Acids Res. **31**:5654-5666. https://doi.org/10.1093/nar/gkg770.

Hanada, K., Vallejo, V., Nobuta, K., Slotkin, R.K., Lisch, D., Meyers, B.C., Shiu, S.H., and Jiang, N. (2009). The functional role of pack-MULEs in rice inferred from purifying selection and expression profile. Plant Cell **21**:25-38. 10.1105/tpc.108.063206.

Haniford, D.B. (2006). Transpososome dynamics and regulation in Tn10 transposition. Crit Rev Biochem Mol Biol **41**:407-424. 10.1080/10409230600987415.

Hastings, P.J., Ira, G., and Lupski, J.R. (2009). A microhomology-mediated break-induced replication model for the origin of human copy number variation. PLoS Genet **5**:e1000327. 10.1371/journal.pgen.1000327.

Hedges, D.J., and Deininger, P.L. (2007). Inviting instability: Transposable elements, double-strand breaks, and the maintenance of genome integrity. Mutat Res **616**:46-59. 10.1016/j.mrfmmm.2006.11.021.

Hohjoh, H., and Singer, M.F. (1996). Cytoplasmic ribonucleoprotein complexes containing human LINE-1 protein and RNA. EMBO J **15**:630-639.

Holmes, S.E., Dombroski, B.A., Krebs, C.M., Boehm, C.D., and Kazazian, H.H., Jr. (1994). A new retrotransposable human L1 element from the LRE2 locus on

chromosome 1q produces a chimaeric insertion. Nat. Genet. **7**:143-148. 10.1038/ng0694-143.

Holt, C., and Yandell, M. (2011). MAKER2: an annotation pipeline and genome-database management tool for second-generation genome projects. BMC Bioinf. **12**:491. https://doi.org/10.1186/1471-2105-12-491.

Hu, J., Fan, J., Sun, Z., and Liu, S. (2020). NextPolish: a fast and efficient genome polishing tool for long-read assembly. Bioinformatics **36**:2253-2255. https://doi.org/10.1093/bioinformatics/btz891.

Hu, X.G., Liu, H., Jin, Y., Sun, Y.Q., Li, Y., Zhao, W., El-Kassaby, Y.A., Wang, X.R., and Mao, J.F. (2016). *De novo* transcriptome assembly and characterization for the widespread and stress-tolerant conifer *Platycladus orientalis*. PLoS One **11**:e0148985. https://doi.org/10.1371/journal.pone.0148985.

Huerta-Cepas, J., Forslund, K., Coelho, L.P., Szklarczyk, D., Jensen, L.J., von Mering, C., and Bork, P. (2017). Fast genome-wide functional annotation through orthology assignment by eggNOG-Mapper. Mol. Biol. Evol. **34**:2115-2122. https://doi.org/10.1093/molbev/msx148.

Ilık, İ.A., Yang, X., Zhang, Z.Z., and Aktaş, T. (2025). Transcriptional and post-transcriptional regulation of transposable elements and their roles in development and disease. Nat. Rev. Mol. Cell Biol. **26**:759-775.

Inoue, K., Dewar, K., Katsanis, N., Reiter, L.T., Lander, E.S., Devon, K.L., Wyman, D.W., Lupski, J.R., and Birren, B. (2001). The 1.4-Mb CMT1A Duplication/HNPP Deletion Genomic Region Reveals Unique Genome Architectural Features and Provides Insights into the Recent Evolution of New Genes. Genome Res. **11**:1018-1033. 10.1101/gr.180401.

Jangam, D., Feschotte, C., and Betran, E. (2017). Transposable Element Domestication As an Adaptation to Evolutionary Conflicts. Trends Genet **33**:817-831. 10.1016/j.tig.2017.07.011.

Jiang, N., Gao, D., Xiao, H., and van der Knaap, E. (2009). Genome organization of the tomato sun locus and characterization of the unusual retrotransposon Rider. Plant J. **60**:181-193. 10.1111/j.1365-313X.2009.03946.x.

Jiang, N., Bao, Z., Zhang, X., Eddy, S.R., and Wessler, S.R. (2004). Pack-MULE transposable elements mediate gene evolution in plants. Nature **431**:569-573. https://doi.org/10.1038/nature02953.

Jin, Y., Zhao, W., Nie, S., Liu, S.S., El-Kassaby, Y.A., Wang, X.R., and Mao, J.F. (2019). Genome-wide variant identification and high-density genetic map construction using RADseq for *Platycladus orientalis* (Cupressaceae). G3: Genes, Genomes, Genet. **9**:3663-3672. https://doi.org/10.1534/g3.119.400684.

Kaessmann, H., Vinckenbosch, N., and Long, M. (2009). RNA-based gene duplication: mechanistic and evolutionary insights. Nat Rev Genet **10**:19-31. 10.1038/nrg2487.

Kapitonov, V.V., and Jurka, J. (2001). Rolling-circle transposons in eukaryotes. Proc. Natl. Acad. Sci. U. S. A. **98**:8714-8719. 10.1073/pnas.151269298.

Kapitonov, V.V., and Jurka, J. (2005). RAG1 core and V(D)J recombination signal sequences were derived from

transposons. Plos Biol **3**:998-1011. ARTN e181

10.1371/journal.pbio.0030181.

Kapitonov, V.V., and Jurka, J. (2008). A universal classification of eukaryotic transposable elements implemented in Repbase. Nat. Rev. Genet. **9**:411-412; author reply 414. 10.1038/nrg2165-c1.

Kapusta, A., Kronenberg, Z., Lynch, V.J., Zhuo, X., Ramsay, L., Bourque, G., Yandell, M., and Feschotte, C. (2013). Transposable elements are major contributors to the origin, diversification, and regulation of vertebrate long noncoding RNAs. PLoS Genet **9**:e1003470. 10.1371/journal.pgen.1003470.

Kazazian, H.H., Jr., and Goodier, J.L. (2002). LINE drive. retrotransposition and genome instability. Cell **110**:277-280. 10.1016/s0092-8674(02)00868-1.

Kim, D., Langmead, B., and Salzberg, S.L. (2015). HISAT: a fast spliced aligner with low memory requirements. Nat. Methods **12**:357-360. https://doi.org/10.1038/nmeth.3317.

Kiyosawa, H., and Chance, P.F. (1996). Primate origin of the CMT1A-REP repeat and analysis of a putative transposon-associated recombinational hotspot. Hum Mol Genet **5**:745-753. 10.1093/hmg/5.6.745.

Kleckner, N. (1981). Transposable elements in prokaryotes. Annu. Rev. Genet. **15**:341-404. 10.1146/annurev.ge.15.120181.002013.

Koren, S., Walenz, B.P., Berlin, K., Miller, J.R., Bergman, N.H., and Phillippy, A.M. (2017). Canu: scalable and accurate long-read assembly via adaptive k-mer weighting and repeat separation. Genome Res. **27**:722-736. https://doi.org/10.1101/gr.215087.116.

Lamesch, P., Berardini, T.Z., Li, D., Swarbreck, D., Wilks, C., Sasidharan, R., Muller, R., Dreher, K., Alexander, D.L., Garcia-Hernandez, M., et al. (2012). The Arabidopsis Information Resource (TAIR): improved gene annotation and new tools. Nucleic Acids Res. **40**:D1202-1210. https://doi.org/10.1093/nar/gkr1090.

Lee, J.A., Carvalho, C.M., and Lupski, J.R. (2007). A DNA replication mechanism for generating nonrecurrent rearrangements associated with genomic disorders. Cell **131**:1235-1247. 10.1016/j.cell.2007.11.037.

Lev-Maor, G., Sorek, R., Shomron, N., and Ast, G. (2003). The birth of an alternatively spliced exon: 3' splice-site selection in Alu exons. Science **300**:1288-1291. 10.1126/science.1082588.

Li, H. (2018). Minimap2: pairwise alignment for nucleotide sequences. Bioinformatics **34**:3094-3100.

Liu, H., Wu, S., Li, A., and Ruan, J. (2021a). SMARTdenovo: a *de novo* assembler using long noisy reads. GigaByte **2021**:gigabyte15. https://doi.org/10.46471/gigabyte.15.

Liu, H., Wang, X., Wang, G., Cui, P., Wu, S., Ai, C., Hu, N., Li, A., He, B., Shao, X., et al. (2021b). The nearly complete genome of *Ginkgo biloba* illuminates gymnosperm evolution. Nat. Plants **7**:748-756. https://doi.org/10.1038/s41477-021-00933-x.

Liu, Y., Wang, S., Li, L., Yang, T., Dong, S., Wei, T., Wu, S., Liu, Y., Gong, Y., Feng, X., et al. (2022). The *Cycas* genome and the early evolution of seed plants. Nat. Plants **8**:389-401. https://doi.org/10.1038/s41477-022-01129-7.

Long, M., and Langley, C.H. (1993). Natural selection and the origin of jingwei, a chimeric processed functional gene in Drosophila. Science **260**:91-95. 10.1126/science.7682012.

Long, M., Betran, E., Thornton, K., and Wang, W. (2003). The origin of new genes: glimpses from the young and old. Nat. Rev. Genet. **4**:865-875. 10.1038/nrg1204.

Luan, D.D., Korman, M.H., Jakubczak, J.L., and Eickbush, T.H. (1993). Reverse transcription of R2Bm RNA is primed by a nick at the chromosomal target site: A mechanism for non-LTR retrotransposition. Cell **72**:595-605. 10.1016/0092-8674(93)90078-5.

Lupski, J.R. (1998). Genomic disorders: structural features of the genome can lead to DNA rearrangements and human disease traits. Trends Genet **14**:417-422. 10.1016/s0168-9525(98)01555-8.

Ma, H., Wang, M., Zhang, Y.E., and Tan, S. (2023). The power of "controllers": Transposon-mediated duplicated genes evolve towards neofunctionalization. J Genet Genomics **50**:462-472. 10.1016/j.jgg.2023.04.003.

Marçais, G., and Kingsford, C. (2011). A fast, lock-free approach for efficient parallel counting of occurrences of *k*-mers. Bioinformatics **27**:764-770. https://doi.org/10.1093/bioinformatics/btr011.

Maron, L.G., Guimarães, C.T., Kirst, M., Albert, P.S., Birchler, J.A., Bradbury, P.J., Buckler, E.S., Coluccio, A.E., Danilova, T.V., and Kudrna, D. (2013). Aluminum tolerance in maize is associated with higher MATE1 gene copy number. Proc. Natl. Acad. Sci. U.S.A. **110**:5241-5246.

McCarrey, J.R., and Thomas, K. (1987). Human testis-specific PGK gene lacks introns and possesses characteristics of a processed gene. Nature **326**:501-505. 10.1038/326501a0.

Mendes, F.K., Vanderpool, D., Fulton, B., and Hahn, M.W. (2021). CAFE 5 models variation in evolutionary rates among gene families. Bioinformatics **36**:5516-5518. https://doi.org/10.1093/bioinformatics/btaa1022.

Moran, J.V., DeBerardinis, R.J., and Kazazian, H.H., Jr. (1999). Exon shuffling by L1 retrotransposition. Science **283**:1530-1534. 10.1126/science.283.5407.1530.

Morgante, M., Brunner, S., Pea, G., Fengler, K., Zuccolo, A., and Rafalski, A. (2005). Gene duplication and exon shuffling by helitron-like transposons generate intraspecies diversity in maize. Nat. Genet. **37**:997-1002. 10.1038/ng1615.

Nassif, N., Penney, J., Pal, S., Engels, W.R., and Gloor, G.B. (1994). Efficient copying of nonhomologous sequences from ectopic sites via P-element-induced gap repair. Mol Cell Biol **14**:1613-1625. 10.1128/mcb.14.3.1613-1625.1994.

Neale, D.B., McGuire, P.E., Wheeler, N.C., Stevens, K.A., Crepeau, M.W., Cardeno, C., Zimin, A.V., Puiu, D., Pertea, G.M., Sezen, U.U., et al. (2017). The Douglas-fir genome sequence rreveals specialization of the photosynthetic apparatus in Pinaceae. G3: Genes, Genomes, Genet. **7**:3157-3167. https://doi.org/10.1534/g3.117.300078.

Nevers, Y., Warwick Vesztrocy, A., Rossier, V., Train, C.-M., Altenhoff, A., Dessimoz, C., and Glover, N.M. (2024). Quality assessment of gene repertoire annotations with OMArk. Nat. Biotechnol.:1-10. https://doi.org/10.1038/s41587-024-02147-w.

Nguyen, L.T., Schmidt, H.A., von Haeseler, A., and Minh, B.Q. (2015). IQ-TREE: a fast and effective stochastic algorithm for estimating maximum-likelihood phylogenies. Mol. Biol. Evol. **32**:268-274. https://doi.org/10.1093/molbev/msu300.

Niu, S., Li, J., Bo, W., Yang, W., Zuccolo, A., Giacomello, S., Chen, X., Han, F., Yang, J., Song, Y., et al. (2022). The Chinese pine genome and methylome unveil key features of conifer evolution. Cell **185**:204-217 e214. https://doi.org/10.1016/j.cell.2021.12.006.

Ohno, S. (1970). Evolution by Gene Duplication (Springer-Verlag). 10.1007/978-3-642-

86659-3.

Panariello, F., Stambolliu, A., Panepuccia, M., Burattin, F.V., and Bodega, B. (2025). Transposable Element-Derived Noncoding RNAs. In Transposable Elements, Transcriptomics, and Diseases, (Springer: pp. 153-189.

Pertea, M., Pertea, G.M., Antonescu, C.M., Chang, T.C., Mendell, J.T., and Salzberg, S.L. (2015). StringTie enables improved reconstruction of a transcriptome from RNA-seq reads. Nat. Biotechnol. **33**:290-295. https://doi.org/10.1038/nbt.3122.

Potrzebowski, L., Vinckenbosch, N., Marques, A.C., Chalmel, F., Jegou, B., and Kaessmann, H. (2008). Chromosomal gene movements reflect the recent origin and biology of therian sex chromosomes. PLoS Biol **6**:e80. 10.1371/journal.pbio.0060080.

Pritham, E.J., and Feschotte, C. (2007). Massive amplification of rolling-circle transposons in the lineage of the bat

Myotis lucifugus. Proc. Natl. Acad. Sci. U.S.A. **104**:1895-1900. 10.1073/pnas.0609601104.

Pryszcz, L.P., and Gabaldon, T. (2016). Redundans: an assembly pipeline for highly heterozygous genomes. Nucleic Acids Res. **44**:e113. https://doi.org/10.1093/nar/gkw294.

Remnant, E.J., Good, R.T., Schmidt, J.M., Lumb, C., Robin, C., Daborn, P.J., and Batterham, P. (2013). Gene duplication in the major insecticide target site, Rdl, in Drosophila melanogaster. Proc. Natl. Acad. Sci. U. S. A. **110**:14705-14710. 10.1073/pnas.1311341110.

Ren, R., Wang, H., Guo, C., Zhang, N., Zeng, L., Chen, Y., Ma, H., and Qi, J. (2018). Widespread whole genome duplications contribute to genome complexity and species diversity in angiosperms. Mol. Plant **11**:414-428. https://doi.org/10.1016/j.molp.2018.01.002.

Rosso, L., Marques, A.C., Weier, M., Lambert, N., Lambot, M.A., Vanderhaeghen, P., and Kaessmann, H. (2008). Birth and rapid subcellular adaptation of a hominoid-specific CDC14 protein. PLoS Biol **6**:e140. 10.1371/journal.pbio.0060140.

Sasaki, M., Lange, J., and Keeney, S. (2010). Genome destabilization by homologous recombination in the germ line. Nat Rev Mol Cell Biol **11**:182-195. 10.1038/nrm2849.

Scott, A.D., Zimin, A.V., Puiu, D., Workman, R., Britton, M., Zaman, S., Caballero, M., Read, A.C., Bogdanove, A.J., Burns, E., et al. (2020). A reference genome sequence for giant sequoia. G3: Genes, Genomes, Genet. **10**:3907-3919. https://doi.org/10.1534/g3.120.401612.

Siguier, P., Gourbeyre, E., and Chandler, M. (2014). Bacterial insertion sequences: their genomic impact and diversity. FEMS Microbiol. Rev. **38**:865-891. 10.1111/1574-6976.12067.

Simão, F.A., Waterhouse, R.M., Ioannidis, P., Kriventseva, E.V., and Zdobnov, E.M. (2015). BUSCO: assessing genome assembly and annotation completeness with single-copy orthologs. Bioinformatics **31**:3210-3212. https://doi.org/10.1093/bioinformatics/btv351.

Slater, G.S., and Birney, E. (2005). Automated generation of heuristics for biological sequence comparison. BMC Bioinf. **6**:31. https://doi.org/10.1186/1471-2105-6-31.

Song, C., Fu, F., Yang, L., Niu, Y., Tian, Z., He, X., Yang, X., Chen, J., Sun, W., Wan, T., et al. (2021). *Taxus yunnanensis* genome offers insights into gymnosperm phylogeny and taxol production. Commun. Biol. **4**:1203. https://doi.org/10.1038/s42003-021-02697-8.

Sorek, R. (2007). The birth of new exons: mechanisms and evolutionary consequences. RNA **13**:1603-1608. 10.1261/rna.682507.

Sorek, R., Ast, G., and Graur, D. (2002). Alu-containing exons are alternatively spliced. Genome Res. **12**:1060-1067. 10.1101/gr.229302.

Stanke, M., Diekhans, M., Baertsch, R., and Haussler, D. (2008). Using native and syntenically mapped cDNA alignments to improve *de novo* gene finding. Bioinformatics **24**:637-644. https://doi.org/10.1093/bioinformatics/btn013.

Startek, M., Szafranski, P., Gambin, T., Campbell, I.M., Hixson, P., Shaw, C.A., Stankiewicz, P., and Gambin, A. (2015). Genome-wide analyses of LINE-LINE-mediated nonallelic homologous recombination. Nucleic Acids Res. **43**:2188-2198. 10.1093/nar/gku1394.

Stevens, K.A., Wegrzyn, J.L., Zimin, A., Puiu, D., Crepeau, M., Cardeno, C., Paul, R., Gonzalez-Ibeas, D., Koriabine, M., Holtz-Morris, A.E., et al. (2016). Sequence of the sugar pine megagenome. Genetics **204**:1613-1626. https://doi.org/10.1534/genetics.116.193227.

Sun, P., Jiao, B., Yang, Y., Shan, L., Li, T., Li, X., Xi, Z., Wang, X., and Liu, J. (2022). WGDI: A user-friendly toolkit for evolutionary analyses of whole-genome duplications and ancestral karyotypes. Mol. Plant **15**:1841-1851. https://doi.org/10.1016/j.molp.2022.10.018.

Tan, S., Cardoso-Moreira, M., Shi, W., Zhang, D., Huang, J., Mao, Y., Jia, H., Zhang, Y., Chen, C., Shao, Y., et al. (2016). LTR-mediated retroposition as a mechanism of RNA-based duplication in metazoans. Genome Res. **26**:1663-1675. 10.1101/gr.204925.116.

Tan, S., Ma, H., Wang, J., Wang, M., Wang, M., Yin, H., Zhang, Y., Zhang, X., Shen, J., Wang, D., et al. (2021). DNA transposons mediate duplications via transposition-independent and -dependent mechanisms in metazoans. Nat. Commun. **12**:4280. https://doi.org/10.1038/s41467-021-24585-9.

Tang, H., Bowers, J.E., Wang, X., Ming, R., Alam, M., and Paterson, A.H. (2008). Synteny and collinearity in plant genomes. Science **320**:486-488. https://doi.org/10.1126/science.1153917.

Thomas, J., Phillips, C.D., Baker, R.J., and Pritham, E.J. (2014). Rolling-circle transposons catalyze genomic innovation in a mammalian lineage. Genome Biol Evol **6**:2595-2610. 10.1093/gbe/evu204.

Thompson, P.J., Macfarlan, T.S., and Lorincz, M.C. (2016). Long Terminal Repeats: From Parasitic Elements to Building Blocks of the Transcriptional Regulatory Repertoire. Mol Cell **62**:766-776. 10.1016/j.molcel.2016.03.029.

Vinckenbosch, N., Dupanloup, I., and Kaessmann, H. (2006). Evolutionary fate of retroposed gene copies in the human genome. Proc. Natl. Acad. Sci. U. S. A. **103**:3220-3225. 10.1073/pnas.0511307103.

Vitte, C., and Panaud, O. (2003). Formation of solo-LTRs through unequal homologous recombination counterbalances amplifications of LTR retrotransposons in rice Oryza sativa L. Mol. Biol. Evol. **20**:528-540. 10.1093/molbev/msg055.

Vitte, C., and Bennetzen, J.L. (2006). Analysis of retrotransposon structural diversity uncovers properties and propensities in angiosperm genome evolution. Proc. Natl. Acad. Sci. U. S. A. **103**:17638-17643. 10.1073/pnas.0605618103.

Wan, T., Liu, Z., Leitch, I.J., Xin, H., Maggs-Kolling, G., Gong, Y., Li, Z., Marais, E., Liao, Y., Dai, C., et al. (2021). The *Welwitschia* genome reveals a unique biology underpinning extreme longevity in deserts. Nat. Commun. **12**:4247. https://doi.org/10.1038/s41467-021-24528-4.

Wan, T., Liu, Z.M., Li, L.F., Leitch, A.R., Leitch, I.J., Lohaus, R., Liu, Z.J., Xin, H.P., Gong, Y.B., Liu, Y., et al. (2018). A genome for gnetophytes and early evolution of seed plants. Nat. Plants **4**:82-89. https://doi.org/10.1038/s41477-017-0097-2.

Wang, D., Zhang, Y., Zhang, Z., Zhu, J., and Yu, J. (2010). KaKs_Calculator 2.0: a toolkit incorporating gamma-series methods and sliding window strategies. Genomics Proteomics Bioinformatics **8**:77-80. https://doi.org/10.1016/S1672-0229(10)60008-3.

Wang, R., van Dorp, L., Shaw, L.P., Bradley, P., Wang, Q., Wang, X., Jin, L., Zhang, Q., Liu, Y., Rieux, A., et al. (2018). The global distribution and spread of the mobilized colistin resistance gene mcr-1. Nat. Commun. **9**:1179. 10.1038/s41467-018-03205-z.

Wang, W., Brunet, F.G., Nevo, E., and Long, M. (2002). Origin of sphinx, a young chimeric RNA gene in Drosophila melanogaster. Proc. Natl. Acad. Sci. U. S. A. **99**:4448-4453. 10.1073/pnas.072066399.

Wang, Y., Tang, H., Debarry, J.D., Tan, X., Li, J., Wang, X., Lee, T.H., Jin, H., Marler, B., Guo, H., et al. (2012). MCScanX: a toolkit for detection and evolutionary analysis of gene synteny and collinearity. Nucleic Acids Res. **40**:e49. https://doi.org/10.1093/nar/gkr1293.

Wei, W., Gilbert, N., Ooi, S.L., Lawler, J.F., Ostertag, E.M., Kazazian, H.H., Boeke, J.D., and Moran, J.V. (2001). Human L1 retrotransposition: cis preference versus trans complementation. Mol Cell Biol **21**:1429-1439. 10.1128/MCB.21.4.1429-1439.2001.

Wicker, T., Buchmann, J.P., and Keller, B. (2010). Patching gaps in plant genomes results in gene movement and erosion of colinearity. Genome Res. **20**:1229-1237. https://doi.org/10.1101/gr.107284.110.

Wicker, T., Sabot, F., Hua-Van, A., Bennetzen, J.L., Capy, P., Chalhoub, B., Flavell, A., Leroy, P., Morgante, M., Panaud, O., et al. (2007). A unified classification system for eukaryotic transposable elements. Nat. Rev. Genet. **8**:973-982. https://doi.org/10.1038/nrg2165.

Xiao, H., Jiang, N., Schaffner, E., Stockinger, E.J., and van der Knaap, E. (2008). A retrotransposon-mediated gene duplication underlies morphological variation of tomato fruit. Science **319**:1527-1530. 10.1126/science.1153040.

Xie, J., Chen, Y., Cai, G., Cai, R., Hu, Z., and Wang, H. (2023). Tree Visualization By One Table (tvBOT): a web application for visualizing, modifying and annotating phylogenetic trees. Nucleic Acids Res. **51**:W587-W592. https://doi.org/10.1093/nar/gkad359.

Xing, J., Wang, H., Belancio, V.P., Cordaux, R., Deininger, P.L., and Batzer, M.A. (2006). Emergence of primate genes by retrotransposon-mediated sequence transduction. Proc. Natl. Acad. Sci. U. S. A. **103**:17608-17613. 10.1073/pnas.0603224103.

Xiong, X., Gou, J., Liao, Q., Li, Y., Zhou, Q., Bi, G., Li, C., Du, R., Wang, X., and Sun, T. (2021). The *Taxus* genome provides insights into paclitaxel biosynthesis. Nat. Plants **7**:1026-1036. https://doi.org/10.1038/s41477-021-00963-5.

Yang, Z. (2007). PAML 4: phylogenetic analysis by maximum likelihood. Mol. Biol. Evol. **24**:1586-1591. https://doi.org/10.1093/molbev/msm088.

Zhang, F., Carvalho, C.M., and Lupski, J.R. (2009a). Complex human chromosomal and

genomic rearrangements. Trends Genet **25**:298-307. 10.1016/j.tig.2009.05.005.

Zhang, J., Zhang, F., and Peterson, T. (2006). Transposition of reversed Ac element ends generates novel chimeric genes in maize. PLoS Genet **2**:e164. 10.1371/journal.pgen.0020164.

Zhang, J., Yu, C., Pulletikurti, V., Lamb, J., Danilova, T., Weber, D.F., Birchler, J., and Peterson, T. (2009b). Alternative Ac/Ds transposition induces major chromosomal rearrangements in maize. Genes Dev **23**:755-765. 10.1101/gad.1776909.

Zhang, R.G., Li, G.Y., Wang, X.L., Dainat, J., Wang, Z.X., Ou, S., and Ma, Y. (2022). TEsorter: an accurate and fast method to classify LTR-retrotransposons in plant genomes. Hortic. Res. **9**:uhac017. https://doi.org/10.1093/hr/uhac017.

Zhang, Z., Xiao, J., Wu, J., Zhang, H., Liu, G., Wang, X., and Dai, L. (2012). ParaAT: a parallel tool for constructing multiple protein-coding DNA alignments. Biochem. Biophys. Res. Commun. **419**:779-781. https://doi.org/10.1016/j.bbrc.2012.02.101.

Zhao, D., Hamilton, J.P., Vaillancourt, B., Zhang, W., Eizenga, G.C., Cui, Y., Jiang, J., Buell, C.R., and Jiang, N. (2018). The unique epigenetic features of Pack-MULEs and their impact on chromosomal base composition and expression spectrum. Nucleic Acids Res **46**:2380-2397. 10.1093/nar/gky025.

Zhou, B., Qi, F., Wu, F., Nie, H., Song, Y., Shao, L., Han, J., Wu, Z., Saiyin, H., Wei, G., et al. (2019). Endogenous Retrovirus-Derived Long Noncoding RNA Enhances Innate Immune Responses via Derepressing RELA Expression. mBio **10**:10.1128/mbio. 00937-00919. 10.1128/mBio.00937-19.
